# Design, synthesis and activity evaluation of tetrahydroisoquinoline‐based programmed cell death ligand 1 inhibitors

**DOI:** 10.1002/smo2.70075

**Published:** 2026-07-28

**Authors:** Menglin Yu, Sen Cai, Yanyan Pan, Heshuang Wang, Fengwu Zhang, Aiyu Ma, Shixuan Lv, Xiuhan Guo, Shisheng Wang, Qiling Song, Chunyan Ma, Shuai Wang, Li Zhang, Jian Wang, Qingwei Meng, Yueqing Li

**Affiliations:** ^1^ State Key Laboratory of Fine Chemicals Department of Pharmaceutical Engineering School of Chemical Engineering Dalian University of Technology Dalian Liaoning China; ^2^ Department of Central Laboratory Dalian Municipal Central Hospital Dalian Liaoning China; ^3^ Ningbo Institute of Dalian University of Technology Ningbo China; ^4^ Viwit Pharmaceuticals Co., Ltd Zaozhuang Shandong China

**Keywords:** docking calculations, HTRF, PD‐L1 inhibitors, pharmacophore modeling, SPR, tetrahydroisoquinoline

## Abstract

Small molecule blockade of the programmed death receptor 1 (PD‐1)/programmed cell death ligand 1 (PD‐L1) pathway represents a promising approach for tumor immunotherapy. Based on the previously developed 3D‐quantitative structure‐activity relationship pharmacophore model for PD‐L1 inhibitors, virtual screening followed by homogeneous time‐resolved fluorescence (HTRF) activity testing identified the compound {1‐[(biphenyl‐4‐yl)methyl]‐7‐methoxy‐1,2,3,4‐tetrahydroisoquinolin‐6‐yl}acetic acid methyl ester (Compound **Y6**) as a hit. Analysis of computational docking results of compound **Y6** with PD‐L1 suggested targeting the methyl acetate substituent at position 6 for optimization. Guided by this, 20 new derivatives were designed and synthesized. The synthesized compounds were subjected to HTRF test and 6 compounds with significant protein blocking effects were screened for subsequent surface plasmon resonance (SPR) test. Subsequent SPR analysis confirmed strong binding of these 6 compounds to hPD‐L1 protein with *K*_D_ values ranging from 0.24 to 21.31 μM. Several derivatives displayed improved or comparable PD‐L1 binding affinity relative to the lead compound **Y6** (*K*_D_ = 11.3 μM). A co‐incubation system (PD‐1^+^ Jurkat T/PD‐L1^+^ HepG2) was established to evaluate functional immune restoration. This evaluation revealed that compound **Y7f** effectively promoted HepG2 cells death by restoring T cell immune function. MD simulations identify **(1R,16R)‐Y7f** as the most potent PD‐L1 dimerization inducer within the compound **Y7f** stereoisomer series. The results indicated that the biphenyl‐tetrahydroisoquinoline scaffold is a promising structural framework for developing novel PD‐1/PD‐L1 inhibitors and deserves further investigation.

## Graphical abstract

Based on a 3D‐quantitative structure‐activity relationship pharmacophore model, structural optimization of **Y6** yielded **Y7f**. Mechanistic studies revealed that **Y7f** acted as a programmed death receptor 1/programmed cell death ligand 1 (PD‐L1) interaction inhibitor by inducing PD‐L1 dimerization. **Y7f** restored T‐cell function. These findings highlight the biphenyl‐tetrahydroisoquinoline scaffold as a promising framework for novel immunotherapeutic agents.

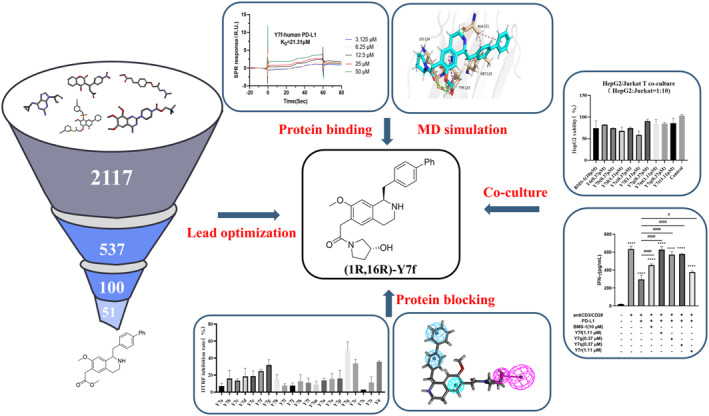

## 1 INTRODUCTION

International Agency for Research on Cancer of the World Health Organization released the latest global cancer data for 2022, revealing that the number of new cancer cases worldwide has reached 20 million, with 9.7 million deaths.^[1]^ Cancer has become one of the most prevalent diseases globally, posing a significant threat not only to human health and even life but also causing severe economic burdens. Current cancer treatment modalities include radiotherapy, surgery, biological therapy, chemotherapy, and traditional Chinese medicine treatment.^[2]^

Immune checkpoint inhibitor therapy refers to enhancing the immune activity against tumor cells by inhibiting negative immune regulation. It has been widely used in clinical practice, ushering in a new era of immunotherapy for tumor treatment and winning the 2018 Nobel Prize in Physiology or Medicine.[^3^, ^4^, ^5^]

PD‐1 (Programmed Death Receptor 1 expressed by T cells) is a crucial immunoinhibitory protein, while PD‐L1 (Programmed Death‐Ligand 1) serves as the ligand protein for PD‐1. Under normal circumstances, the binding of PD‐1 and PD‐L1 helps prevent the occurrence of autoimmune diseases.^[6]^ However, in the tumor microenvironment (TME), when tumor cells overexpress PD‐L1 and bind to PD‐1, it blocks the T‐cell receptor and downstream signaling pathways, thereby inhibiting the activation and proliferation of T cells. This disruption further impairs the function of effector T cells, enabling tumor cells to evade the immune system.[^7^, ^8^, ^9^, ^10^] Blocking the PD‐1/PD‐L1 binding can rejuvenate T cells, allowing them to kill tumor cells.

Monoclonal antibodies (mAbs) that block the PD‐1/PD‐L1 pathway have been widely used in clinical immunotherapy and have shown promising efficacy against various types of cancers. To date, the Food and Drug Administration has approved a total of nine mAbs for clinical use,[^11^, ^12^, ^13^] including seven PD‐1 mAbs (pembrolizumab, nivolumab, cemiplimab, dostarlimab, retifanlimab, toripalimab, tislelizumab) and four PD‐L1 mAbs (atezolizumab, avelumab, durvalumab, cosibelimab). mAbs possess advantages such as high specificity, high affinity for antigens, and relatively easy development. However, they also present challenges such as high manufacturing costs, stringent transportation/storage conditions, inconvenience in injection administration, immune‐related adverse reactions, and drug resistance.[^14^, ^15^] To address the limitations of mAbs, small‐molecule PD‐L1 inhibitors have emerged as a research hotspot due to their advantages, including low molecular weight, high tissue penetration, controllable pharmacokinetics, and good oral bioavailability.[^16^, ^17^]

**FIGURE 1:**
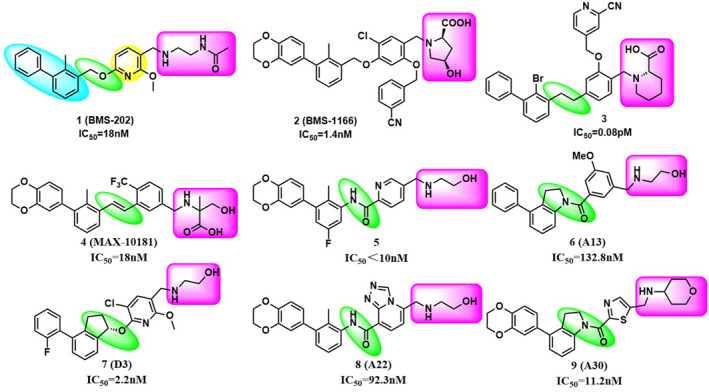
Reported small molecule inhibitors targeting the programmed death receptor 1/programmed cell death ligand 1 pathway (biaryl core, cyan; linker, green; aryl group, yellow; nitrogen‐containing hydrophilic group, pink).

In 2015, Zak et al.^[18]^ resolved the crystal structure of the hPD‐1/hPD‐L1 protein complex (PDB:4ZQK) using X‐ray diffraction, providing a structural basis for the development of small‐molecule drugs targeting PD‐1/PD‐L1. In recent years, significant progress has been made in the field of small‐molecule inhibitors of PD‐1/PD‐L1. For instance, Bristol‐Myers Squibb (BMS) has developed biphenyl derivatives, with representative compound **1** (**BMS‐202**) and compound **2** (**BMS‐1166**) exhibiting IC_50_ values of 18 and 1.4 nM, respectively (Figure 1).[^19^, ^20^] Holak^[21]^ and his colleagues elucidated the mechanism of action of biphenyl‐based small‐molecule compounds using **BMS‐202** as a model compound through X‐ray crystallographic techniques and biochemical experiments. The study revealed that biphenyl‐based small‐molecule compounds bind to the surface of PD‐L1 protein, inducing PD‐L1 dimerization, and **BMS‐202** embeds in the hydrophobic cavity formed by PD‐L1 dimerization. The interaction surface between dimerized PD‐L1/PD‐L1 is highly similar to that of PD‐1/PD‐L1, preventing the normal interaction between PD‐1 and PD‐L1 and ultimately blocking the signaling pathway. Many researchers in subsequent structural designs retained the biphenyl backbone (cyan), and innovatively designed the linking group connected to the biphenyl (green), the substituent aromatic ring (yellow), and the hydrophilic groups on the aromatic ring, taking **BMS‐202** as an example (Figure 1). Feng et al. replaced the linking group and the intermediate aromatic ring, resulting in the representative compound **3** (IC_50_ = 0.08 pM).^[22]^ Some novel, orally administrated aromatic ethylene derivatives by Guangzhou Maxinovel Pharmaceuticals have been tested in clinic trials (such as compound **4**).^[23]^ Some molecules developed by Incyte featuring a biphenyl and an aromatic ring linked by an amide also demonstrated high activity (compound **5**).^[24]^ Based on the ring fusion strategy to alter the amide linker, Qin et al. designed a representative compound **6** (**A13**, IC_50_ = 132.8 nM) containing a dihydroindole moiety^[25]^. Wang's team designed novel indane derivatives as PD‐1/PD‐L1 inhibitors. They found compound **7** (**D3**) showed the most potent activity in PD‐1/PD‐L1 TR‐FRET assay (IC_50_ = 2.2 nM).^[26]^ Qin et al. adopted pharmacophore models and docking‐based ring fusion strategies, yielding representative compounds such as [1,2,4]triazolo[4,3‐a]pyridine (compound **8**, **A22**) with IC_50_ values of 92.3 nM, respectively.^[27]^ Qin et al. further designed a series of biphenyl inhibitors containing a thiazole moiety, the most potent compound **9** (**A30**, IC_50_ = 11.2 nM).^[28]^ Notably, the recent elucidation of high‐resolution structures for clinical candidates with superior potency (e.g., PDB 9HRT, 9I0U, 9I0W) represents the current vanguard of PD‐L1 inhibitor design.^[29]^ Although our work originated from the classic BMS scaffold, these newly emerging structures exemplify the dynamic nature of this research area and validate the ongoing pursuit of structurally diverse inhibitors through scaffold optimization.

In this research, a small molecule {1‐[(biphenyl‐4‐yl)methyl]‐7‐methoxy‐1,2,3,4‐tetrahydroisoquinoline‐6‐yl}acetate (compound **Y6**), was screened out by a 3D‐quantitative structure‐activity relationship (QSAR) pharmacophore model (Figure S1).^[30]^ It was found to inhibit PD‐1/PD‐L1 binding by 58% at a concentration of 100 μM in homogeneous time‐resolved fluorescence (HTRF) screening. Tetrahydroisoquinoline, serving dual functions as both a linker and an aromatic moiety in PD‐L1 small molecule inhibitors, has not yet been reported in the literature. Thus, we conducted docking calculations for compound **Y6** with PD‐L1. Based on the results, we thought the modification of 6‐position acetate methyl ester could be useful to design derivatives and introduce nitrogen‐containing hydrophilic group. For the reported PD‐1/PD‐L1 small molecule inhibitors, the presence of hydrophilic nitrogen‐containing side chains exerts a considerable influence on the activity of molecules sharing the same core skeleton. Such side chains from the literatures were selected and used in the design of new derivatives of compound **Y6** (Figure S2). We conducted pharmacophore mapping analysis followed by molecular docking calculations. The inhibitory activity of small molecules against the PD‐1/PD‐L1 interaction was evaluated using HTRF assays, while surface plasmon resonance (SPR) measurements determined the binding affinity of active compounds to PD‐L1. Finally, in a HepG2/Jurkat co‐culture system, compound **Y7f** effectively restored T‐cell immune function and enhanced tumor cell death, demonstrating therapeutic potential.

## 2 RESULTS AND DISCUSSION

### 2.1 Discovery of lead compounds

Our group constructed a robust 3D‐QSAR pharmacophore model of PD‐L1 inhibitors based on the small molecule structure using Discovery Studio software (Figure S1).^[30]^ It was validated by internal methods (Cost validation, Fisher validation), an external method (maximum unbiased validation) and the performance of virtual screening. It also applied in this study.

The pre‐virtual screening of Pharmaceutical Substances of Thieme Chemistry and our laboratory's collection of small molecules totaled 2117, including 207 compounds from our lab. 51 promising molecules were chosen for the initial round of activity testing. According to the results of HTRF activity test, the laboratory compound **Y6**, {1‐[(biphenyl‐4‐yl)methyl]‐7‐methoxy‐1,2,3,4‐tetrahydroisoquinolin‐6‐yl}acetic acid methyl ester, displayed an inhibition rate of 58% at 100 μM, signifying a moderate blockage effect. This particular structure has not previously been reported in the literature as a small molecule inhibitor targeting PD‐L1. Unlike conventional PD‐L1 inhibitors, compound **Y6** incorporates a novel tetrahydroisoquinoline scaffold despite retaining the ubiquitous biphenyl pharmacophore.

**FIGURE 2:**
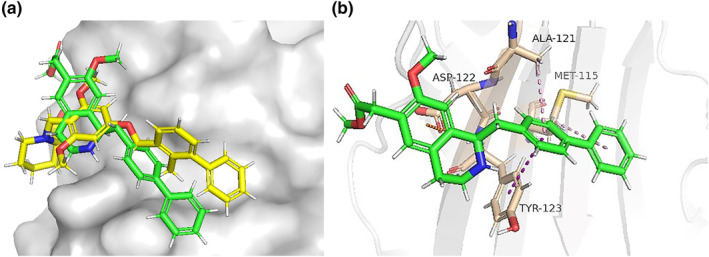
(a) Overlapping structure of compound **Y6** (green sticks) and **BMS‐1** (yellow sticks) structure at the binding interface of PD‐L1 (PDB ID: 4ZQK). (b) Molecular docking of compound **Y6** to the PD‐L1 binding mode. PD‐L1 was displayed as white ribbon and the interacting amino acids showed as wheat stick. All the π‐Alkyl interaction were showed as pink dash and π–π interaction was displayed as purple dash. The orange dash represents π‐anion interaction. PD‐L1, programmed cell death ligand 1.

In 2016, researchers resolved the crystal structure of PD‐L1 dimer in complex with small‐molecule inhibitor and small‐molecule inhibitors could induce the formation of PD‐L1 protein homodimers, thereby preventing PD‐L1 from binding to its receptor PD‐1.^[21]^ The crystal structures of PD‐L1 dimer with the small molecular inhibitors were deposited in PDB continuously, which provide rich information about the binding site, and important interactions with key amino acid residues. Based on the crystal structure analysis of 6R3K, the key amino acid residues of the PD‐L1 protein are identified as Met115, Ala121, Asp122, Tyr123, Lys124, and Arg125. After preprocessing and superposition of the 6R3K and 4ZQK crystal structures, the results (Figure 2) showed that the biphenyl portion of compound **Y6** exhibited a similar binding mode to **BMS‐1**. The acetic acid methyl ester of compound **Y6** could not form strong hydrogen bond interaction with PD‐L1 as the tetrahydropyridine‐2‐carboxylic acid group of **BMS‐1**. Therefore, the molecular design involves introducing a nitrogen‐containing hydrophilic side chain at position 6 of compound **Y6** to improve its binding affinity for PD‐L1.

### 2.2 Design of target compounds

The modifying groups in the literature[^25^, ^31^, ^32^, ^33^, ^34^, ^35^, ^36^, ^37^, ^38^, ^39^] that contribute more to the activity of PD‐L1 small molecule inhibitors were collected (Figure S2). A total of 20 compounds were designed. Firstly, they were mapped with the pharmacophore model (Figure S1). The majority of the designed compounds scored higher in their alignment with the pharmacophore model than did compound **Y6**. This suggested that the incorporation of a nitrogen‐containing side chain genuinely augmented the active pharmacophore features of the compounds. The higher Fitvalue scores indicated a better match between the molecules and the pharmacophores. The mapping results were shown in Table 1.

**TABLE 1:** FitValues of the designed molecules.

No.	FitValue	No.	Fitvalue	No.	FitValue	No.	FitValue
**Y6**	0.5979	**Y7e**	0.5585	**Y7k**	0.7184	**Y7p**	0.6498
**Y7**	0.5921	**Y7f**	0.6859	**Y7l**	0.7079	**Y7q**	0.5960
**Y7a**	0.5924	**Y7g**	0.6128	**Y7m**	0.6320	**Y7r**	0.7395
**Y7b**	0.7005	**Y7h**	0.6892	**Y7n**	0.6467	**Y7s**	0.7064
**Y7c**	0.7026	**Y7i**	0.6505	**Y7o**	0.6802	**Y7t**	0.5824
**Y7d**	0.5957	**Y7j**	0.7105				

The PD‐L1 protein (Chain A) found within the hPD‐1/hPD‐L1 crystal structure (PDB ID: 4ZQK) was utilized for further molecular docking studies. As indicated in Table S1, the CDocker interaction energy scores of our designed compounds outperformed that of compound **Y6**, suggesting a superior binding affinity of our molecules towards the PD‐L1 protein.

Thus, taking into account the alignment scores of the small molecules with the established pharmacophore models and the insights from molecular docking analyses, the rationale for the synthetic feasibility of the designed compounds was well‐supported.

### 2.3 Chemistry

**SCHEME 1:**
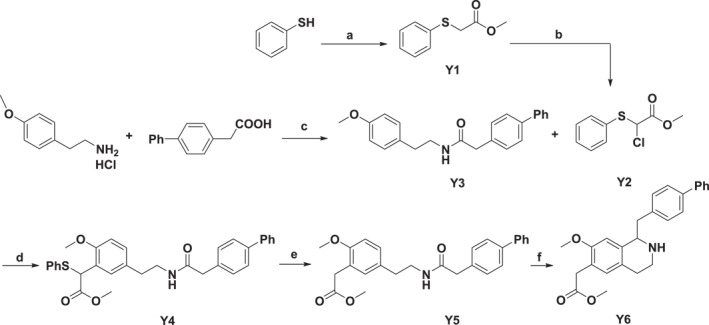
Synthetic route of compound **Y6**. Reagents and conditions: (a) i. Na, PhMe, N_2_, reflux, ii. ClCH_2_COOCH_3_; (b) SO_2_Cl_2_, DCM, −10°C; (c) HBTU, Et_3_N, MeCN, rt; (d) SnCl_4_, DCM, N_2_, rt; (e) Raney Ni, THF, rt; (f) i. POCl_3_, reflux, ii. NaBH_4_, MeOH/DCM = 1:1, 0°C.

The synthesis of compound **Y6** initiated with the reaction between phenylthiophenol and methyl chloroacetate to yield compound **Y1**. Subsequently, compound **Y1** was chlorinated using sulfonyl chloride to produce the alkylating agent **Y2**. The amidation of 2‐(4‐methoxyphenyl)ethylamine hydrochloride and 4‐biphenylacetic acid resulted in compound **Y3**. Under the catalysis of SnCl_4_, an alkylation of compound **Y3** with **Y2** afforded compound **Y4**. Following a dephenylthiophenol reaction to obtain compound **Y5**, the final step involved a Bischler‐Napieralski reaction to synthesize compound **Y6** (Scheme 1).

**SCHEME 2:**
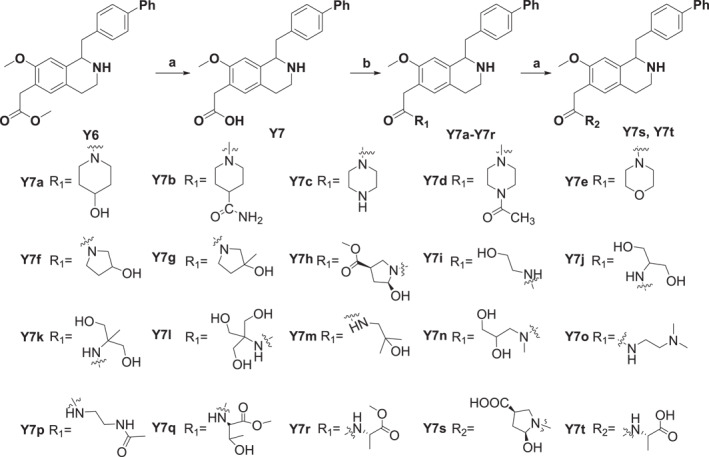
Synthetic route of compounds **Y7a‐Y7t**. Reagents and conditions: (a) 1M NaOH(aq), MeOH and (b) amine compounds, HBTU, DCM, rt.

After a thorough literature review, the substituent with better activity was summarized. Compound **Y6** was hydrolyzed to obtain **Y7**, and the target product was achieved through a condensation reaction between compound **Y7** and the amine compound (Scheme 2).

### 2.4 In vitro PD‐1/PD‐L1 binding (HTRF) assay

**FIGURE 3:**
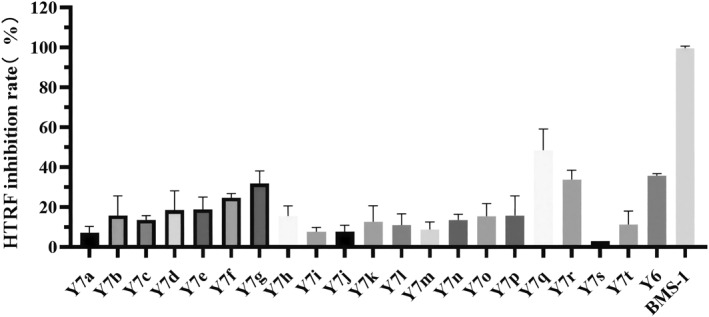
Results of homogeneous time‐resolved fluorescence for blocking programmed death receptor 1/programmed cell death ligand 1 interaction by 22 compounds at a concentration of 50 μM.

HTRF assay was used to evaluate the newly synthesized compounds' inhibition of the PD‐1/PD‐L1 protein interaction. The reference compound **BMS‐1** have proved to be a valuable tool for validating the HTRF assay and establishing a baseline for the initial structure‐activity relationship (SAR) studies.[^25^, ^27^] This allowed us to effectively benchmark the relative improvements of our preferred compounds. As shown in Figure 3, we first investigated the effects of 22 compounds **Y6**, **Y7** and **Y7a** ∼ **Y7t** on the PD‐1/PD‐L1 interaction at a concentration of 50 μM. The presence of a methyl group in the nitro‐containing sidechain could increase the blocking activity of the compound. For example, the 3‐methyl‐pyrrolidin‐3‐ol group on compound **Y7g** has one more methyl group than the pyrrolidin‐3‐ol on compound **Y7f**. The blocking activity of compound **Y7g** was better than that of compound **Y7f**. The similar situation occurred between compounds **Y7m** and **Y7i**, compounds **Y7k** and **Y7j**. The blocking activities of esters (compounds **Y7h** and **Y7q**) were better than those of the corresponding acids (compounds **Y7s** and **Y7t**), suggesting that maintaining a certain lipophilic group in the side chain plays a crucial role in binding to the target.

### 2.5 Pharmacophore matching analysis

**FIGURE 4:**
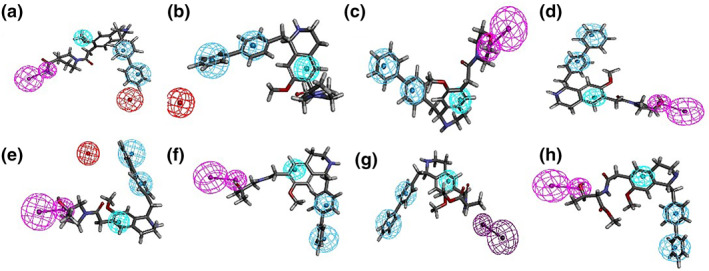
Pharmacophore matching analysis for compounds with better blocking activity. (a) Compound **Y7b**; (b) compound **Y7d**; (c) compound **Y7e**; (d) compound **Y7f**; (e) compound **Y7g**; (f) compound **Y7m**; (g) compound **Y7q**; (h) compound **Y7r**.

Based on the results of the HTRF experiments, we further analyzed the matches of these eight small molecule compounds with the established pharmacophore models. The analysis results showed that all these eight compounds had high FitValues in the pharmacophore model analysis, indicating that they had a good match with the pharmacophore model. More importantly, these compounds all matched multiple key pharmacophore feature elements. As shown in Figure 4, compounds **Y7b**, **Y7e**, **Y7f**, **Y7g**, **Y7m** and **Y7r** matched with three hydrophobic elements and one hydrogen bond donor element. Compounds **Y7d** and **Y7q** matched with three hydrophobic elements. In summary, the selected representative compounds matched multiple hydrophobic pharmacophore features and showed appreciable PD‐1/PD‐L1 blocking effects in the HTRF assay.

### 2.6 SPR analysis

SPR experiments represent an experimental method enabling the direct and quantitative measurement of intermolecular interactions. To further verify whether the preferred compounds bind to hPD‐L1 proteins, the binding affinity of six compounds for hPD‐L1 was evaluated using SPR. First, the affinity of the positive control **BMS‐1** for hPD‐L1 was determined to ensure the activity of the protein on the surface of the chip.

The reference compound **BMS‐1** yielded a *K*_D_ of 27.3 μM in our SPR assay, which was higher than the value of 3.16 μM reported by Wang et al.^[40]^ This discrepancy is not uncommon in biophysical assays and likely stems from differences in experimental conditions, such as the specific recombinant protein constructs and buffer compositions employed. These factors are known to influence absolute kinetic measurements. Importantly, the primary role of **BMS‐1** in this study was to serve as a consistent internal benchmark. Its value lies in enabling the direct and reliable comparison of the relative binding affinities of all novel compounds tested within our unified experimental system. Subsequently, the binding affinity of compound **Y6** as well as the preferred compounds (**Y7b**, **Y7d**, **Y7e**, **Y7f**, **Y7g**, **Y7q**) to the hPD‐L1 protein was determined, and the results were shown in Table 2.

**TABLE 2:** Binding affinity of compounds to hPD‐L1.

Compds	*K*_D_ (μM)	Compds	*K*_D_ (μM)	Compds	*K*_D_ (μM)
**Y7b**	6.16	**Y7f**	21.31	**Y6**	11.30
**Y7d**	4.01	**Y7g**	12.25	**BMS‐1**	27.30
**Y7e**	10.96	**Y7q**	0.24		

The SPR results showed that these derivatives exhibited measurable binding to hPD‐L1, with *K*_D_ values ranging from 0.24 to 21.31 μM. Among them, compound **Y7q** showed the strongest binding affinity with a *K*_D_ value of 0.24 μM. Compounds **Y7d** and **Y7b** also displayed improved binding affinity compared with the lead compound **Y6** (*K*_D_ = 11.30 μM), whereas **Y7e** and **Y7g** showed comparable binding affinity. Compound **Y7f** exhibited relatively weak SPR affinity (*K*_D_ = 21.31 μM).

### 2.7 Docking studies

**FIGURE 5:**
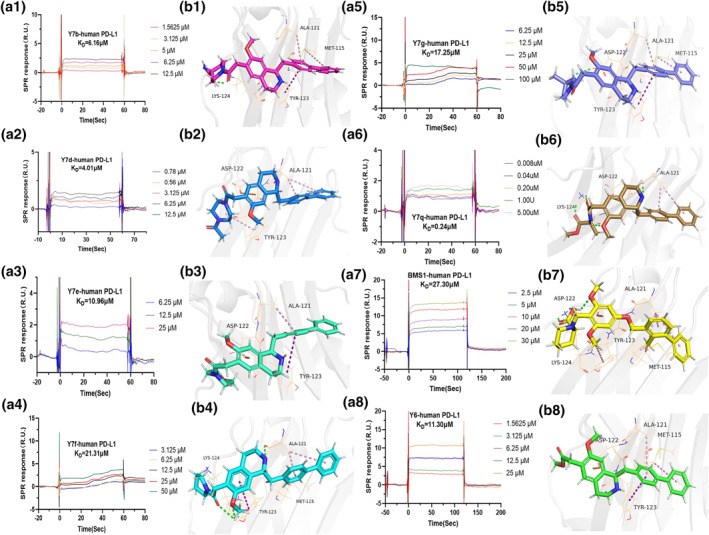
(a1–a8) Binding affinity of compounds to hPD‐L1. (b1–b8) The binding interactions of compound with PD‐L1. PD‐L1 was displayed as white gray ribbon and the interacting amino acids showed as wheat stick. All the hydrogen bonds were showed as green dash and π–π interaction was displayed as purple dash. The pink dash represents π‐alkyl interaction and π‐anion interaction was displayed as orange dash. Compound **Y7b**, **Y7d**, **Y7e**, **Y7f, Y7g**, **Y7q**, **BMS1** and **Y6** shown in magenta, marine, green cyan, cyan, slate, sand, yellow and green, respectively. PD‐L1, programmed cell death ligand 1.

DS CDocker was used to explore the binding pattern between small molecules and hPD‐L1. The binding interactions of eight compounds (**BMS‐1**, **Y6**, **Y7b**, **Y7d**, **Y7e**, **Y7f**, **Y7g**, **Y7q**) with PD‐L1 were modeled. Since SPR results reflected the binding affinity between small molecules and the targeting protein, the docking results were subsequently analyzed and discussed in combination with the SPR experimental test results. Notably, six of the prioritized compounds exhibited a binding pattern similar to **BMS‐1**. The biphenyl moiety penetrated deeply into the hydrophobic cavity, while the polar tail extended towards the solvent‐exposed region, as depicted in Supplementary Figure S3. All designed derivatives have shown higher docking scores than Y6 (Table S1). However, the docking scores do not align precisely with the SPR experimental binding affinities. This observed discrepancy—where improved scoring does not uniformly translate into stronger binding—may arise from inherent methodological differences, such as the static model's inability to fully capture protein flexibility, or minor experimental variations intrinsic to SPR measurements. A detailed analysis of the interactions with key amino acid residues revealed that both compounds **Y7f** displayed π‐alkyl interactions between their biphenyl moieties and Met115, Ala121. Compound **Y7f** further demonstrated a hydrogen bond between the secondary amine on its tetrahydroisoquinoline moiety and Ala121, while its polar tail formed additional hydrogen bonds with Tyr123 and Lys124. Therefore, the compounds preferred by HTRF could form additional hydrogen bonds with critical amino acid residues in docking simulation (Figure 5). This was related with the calculated higher interaction energies and might contribute to their favorable binding affinities as demonstrated in the SPR experiments.

### 2.8 Cytotoxicity assays

Before testing the blockade of PD‐1/PD‐L1 interactions by compounds at the cellular level, the CCK‐8 assay was first used to test the maximum non‐toxic concentrations of the compounds for HepG2 and Jurkat cells. Ensure that during the co‐incubation of HepG2 and Jurkat cells, the killing effect of T cells leads to the reduction of cancer cells. The smaller value of the maximum non‐toxic concentrations of each compound for the two cell types was used for the subsequent cell co‐incubation (Table 3).

**TABLE 3:** Maximum non‐toxic concentration by CCK‐8 method.

Entry	Compds	Maximum non‐toxic concentration^a^	
		HepG2 (μM)	Jurkat (μM)
1	**BMS‐1**	10.00	10.00
2	**Y6**	0.37	0.37
3	**Y7b**	3.33	0.37
4	**Y7d**	3.33	1.11
5	**Y7e**	1.11	0.37
6	**Y7f**	3.33	1.11
7	**Y7g**	3.33	0.37
8	**Y7m**	1.11	1.11
9	**Y7q**	1.11	0.37
10	**Y7r**	3.33	1.11

^a^ Cells were incubated for 48 h and analyzed using the CCK‐8 assay. Each experiment was performed in triplicate (*n* = 3).

### 2.9 In vitro immunoregulatory potency in a HepG2/Jurkat T cell co‐culture model

**FIGURE 6:**
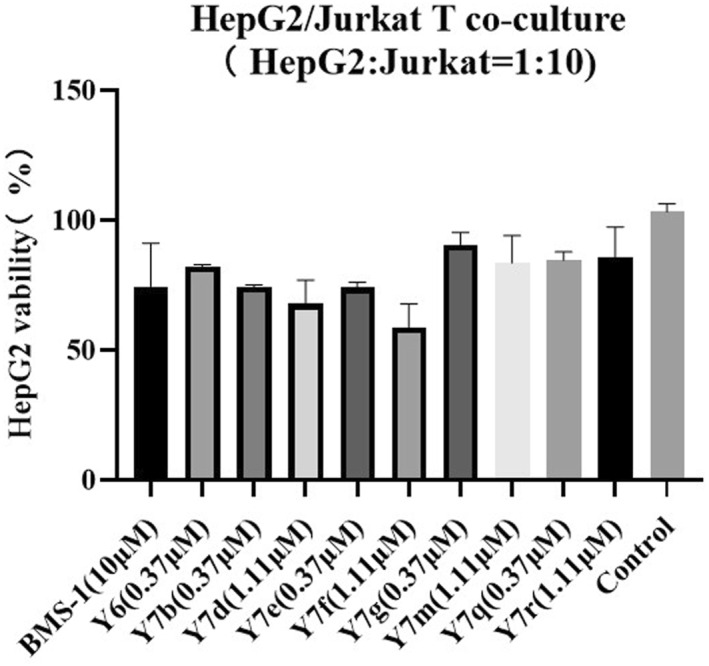
Comparison of 10 compounds on the viability of HepG2 cells in cell co‐incubation.

To assess the efficacy of the compounds in inducing anti‐tumor immunity, the killing effect of the compounds were monitored within a co‐culture system comprised of PD‐1^+^ Jurkat cells and PD‐L1^+^ HepG2 cells^[30]^. HepG2 cells pre‐stimulated with 10 ng/mL interferon‐γ (IFN‐γ) and Jurkat cells activated with 2 μg/mL PHA were cultured together at a 10:1 ratio. Non‐toxic concentrations of compounds were selected for PD‐1/PD‐L1 signaling blockade assays. PD‐1^+^ Jurkat T cells and PD‐L1^+^ HepG2 cultured together without any compound were used as control group. HepG2 cell survival was significantly reduced in the co‐culture system with different small inhibitors (Figure 6). Therefore, the addition of the compounds was effective in blocking the PD‐1/PD‐L1 signaling pathway and activating Jurkat T cells to kill tumor cells. Among them, compound **Y7f** achieved 42% inhibition at a concentration of 1.11 μM and performed better than **BMS‐1** at concentration of 10 μM.

It is worth noting that although no precipitation was observed for any compound at the tested concentrations, minor differences in kinetic solubility may still affect the active concentration over time, potentially influencing the cellular potency. It should be noted that while the HepG2/Jurkat co‐culture system is a well‐established in vitro model for PD‐1/PD‐L1 blockade, it represents a simplified version of the complex TME, lacking key components such as diverse immune subsets, stromal cells, and the extracellular matrix. Therefore, the promising results obtained for compound **Y7f** warrant further validation in more physiologically relevant models, including primary immune cell‐based systems and in vivo tumor models.

### 2.10 T‐cell recovery activity assay in Jurkat cells

**FIGURE 7:**
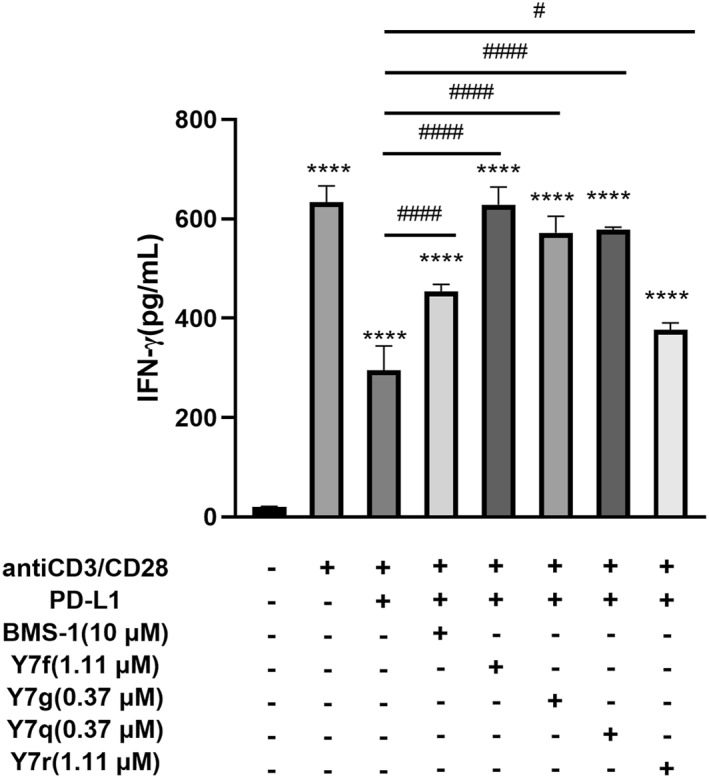
Effects of compounds on IFN‐γ release from Jurkat cells. All data are presented as the mean ± SD, *n* = 3. ****p < 0.0001, ^####^ *p* < 0.0001. IFN‐γ, interferon‐γ.

To further demonstrate that these tetrahydroisoquinoline derivatives block the PD‐1/PD‐L1 cellular axis, the abilities of compounds **Y7f**, **Y7g**, **Y7q**, and **Y7r** to restore T‐cell function in Jurkat cells was evaluated (Figure 7, Figure S4). The PD‐L1 protein inhibits the release of IFN‐γ from Jurkat cells. However, PD‐L1 inhibitors, such as the positive control **BMS‐1**, block the PD‐1/PD‐L1 interaction, thereby rescuing T‐cell function and enhancing IFN‐γ secretion. As depicted in Figure 7, compound **Y7f** at a concentration of 1.11 μM significantly reversed the immunosuppressive effect imposed by the PD‐L1 protein. In contrast, Figure S4 demonstrated that these small molecules alone did not stimulate the release of IFN‐γ from Jurkat cells, indicating the specificity of their mechanism of action. These data confirmed that compound **Y7f**, **Y7g**, and **Y7q** and **Y7r** were PD‐L1 inhibitors. Especially, compound **Y7f** was the most promising. One possible explanation is that compounds **Y7q** and **Y7r** contain ester bonds that are susceptible to hydrolysis by esterases present in the cell culture medium or within cells. This could lead to their degradation over the 48‐h co‐culture period, diminishing their effective concentrations. In contrast, compound **Y7f**, lacking such labile bonds, is likely more stable under the assay conditions.

### 2.11 ALogP

The PD‐1/PD‐L1 signaling pathway belongs to extracellular interactions. Consequently, PD‐1/PD‐L1 small molecule inhibitors can exert a better inhibitory effect when remaining outside the cells. When the ALogP (lipophilicity distribution coefficient) value of a molecule is relatively small, the molecule crosses the cell membrane at a slower rate, and thus can stay outside the cell membrane to exert its therapeutic effects. Therefore, the ALogP of these compounds were calculated in DS (Table 4) and analyzed in combination with their activity.

**TABLE 4:** ALogP calculated in DS.

Compds	ALogP	Compds	ALogP	Compds	ALogP
**Y7b**	3.8	**Y7f**	3.696	**Y7q**	4.311
**Y7d**	3.543	**Y7g**	3.901	**Y7r**	3.801
**Y7e**	4.014	**Y7m**	4.163	**Y6**	4.768

Compound **Y7f** has high blocking activity in cell co‐incubation because of its smaller ALogP and strong ability to block PD‐1/PD‐L1 protein at the molecular level, so more small molecules stay outside the cell membrane and effectively block PD‐1/PD‐L1 protein interactions, which results in better immune efficacy.

The development history of PD‐1/PD‐L1 small‐molecule inhibitors itself serves as an instructive case. Over a decade ago, the first potent series disclosed by Bristol‐Myers Squibb exhibited outstanding activity in biochemical (e.g., HTRF) assays, yet none have progressed to market approval, highlighting the formidable translational gap from in vitro binding to in vivo efficacy. This reinforces the principle that successful lead optimization must extend beyond maximizing affinity to holistically optimize permeability, stability, and selectivity. Therefore, the standout performance of compound **Y7f** in cellular models is particularly encouraging.

### 2.12 Molecular dynamics simulation of compound Y7f with PD‐L1

**FIGURE 8:**
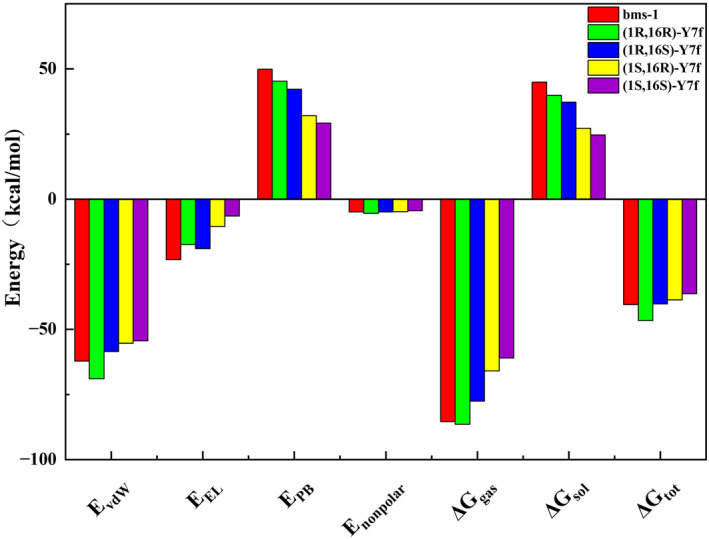
MM/PBSA free energy components for programmed cell death ligand 1 complexes with **(1R, 16R)‐Y7f**, **(1R, 16S)‐Y7f**, **(1S, 16R)‐Y7f**, **(1S, 16S)‐Y7f**, and **BMS‐1**. MM/PBSA, molecular mechanics/Poisson‐Boltzmann surface area.

Molecular dynamics simulations were employed to further investigate the interactions of compound **Y7f** with PD‐L1. Due to the lack of stereoselectivity in the synthesis method for the tetrahydroisoquinoline ring, compound **Y6** is a racemate. When preparing compound **Y7f**, the modifying group employed also contains a single chiral center. Consequently, compound **Y7f** sample tested for biological activity is a mixture of four stereoisomers: **(1R, 16R)‐Y7f, (1R, 16S)‐Y7f, (1S, 16R)‐Y7f** and **(1S, 16S)‐Y7f**. In molecular simulations, all four configurations were evaluated (SI 3 Molecular dynamics simulation, Table S2, Figures S5–S7). The results indicated that the interactions of compound **Y7f** with PD‐L1 were similar to those of **BMS‐1** with PD‐L1 (Figure 8, Table S2). The binding of the small molecule to the protein was primarily driven by hydrophobic interactions (van der Waals, vdW), supplemented by specific electrostatic interactions (*E*_EL_) and hydrophobic effect (*E*_nonpolar_). However, polar desolvation (*E*_PB_) posed a major energetic penalty (Figure S7). Despite this penalty, the total binding free energy (△*G*_tot_) suggested that the binding was strongly spontaneous for four stereoisomers. The results revealed that the R‐configuration at position 1 of the tetrahydroisoquinoline ring was critical for binding energy, while the R‐configuration of the substituent (C16) was energetically favored over the S‐configuration (Figure 8, Table S2).

Analysis of △*G* gas reveals that **(1R, 16R)‐Y7f** exhibits stronger binding to PD‐L1 than **BMS‐1**, while **(1R, 16S)‐Y7f** demonstrates comparable binding affinity to **BMS‐1**. Through per‐residue decomposition of the binding free energy (Figure S6), key residues contributing substantially to ligand binding were identified across all systems. These include ILE54, VAL55, TYR56, GLN66, VAL68, VAL76, CYS114, MET115, GLY120, and TYR123. Critically, this study reveals that MET115 and ALA121 engage in strong hydrophobic interactions with the aromatic system of the compounds.^[41]^ These interactions are robustly reflected in their respective binding free energy contributions, particularly for the 1R‐configured stereoisomer of compound **Y7f**.

The insights gained from MD simulations provide important guidance for future research on tetrahydroisoquinoline‐based PD‐L1 small molecules. First, the stereoselective cyclization conditions for tetrahydroisoquinoline core synthesis should be optimized to control the stereochemistry at the key chiral centers, thereby reducing the formation of unwanted stereoisomers and improving the yield of the potentially active (1R)‐stereoisomer. Second, chiral building blocks should be employed during the introduction of amino acid side chains to achieve stereochemically pure single isomers. Subsequent studies will focus on the isolation or stereoselective synthesis of individual **Y7f** stereoisomers, followed by separate biological validation to confirm the computational prediction that **(1R, 16R)‐Y7f** is the most active stereoisomer. Such efforts will not only clarify the structure–activity relationship of **Y7f** stereoisomers but also lay a solid foundation for the further optimization and development of highly potent and selective tetrahydroisoquinoline‐based PD‐L1 inhibitors.

## 3 CONCLUSIONS

In this paper, the structure of the molecule {1‐[(biphenyl‐4‐yl)methyl]‐7‐methoxy‐1,2,3,4‐tetrahydroisoquinolin‐6‐yl}methyl acetate (**Y6**) was used as a basis for structural modification. Derivatized molecules were designed for the methyl 6‐acetate group based on the docking of compound **Y6** with PD‐L1. The modification moieties in the literature that contribute significantly to the activity of PD‐L1 small molecule inhibitors were collected and a total of 20 compounds were designed and synthesized.

The synthesized compounds were subjected to HTRF test. Among them, the top eight molecules with obvious protein blocking effects were screened for subsequent tests. The reasons for the selection of these molecules were also analyzed with the help of the pharmacophore model matching results. The results of SPR experiments showed that the 6 compounds showed obvious binding to hPD‐L1 protein, with the *K*_D_ values in the range of 0.24–21.31 μM (Figure 5). In the HepG2/Jurkat T cell Co‐culture assay, compound **Y6** and all eight derivatives could activate immune system (Figure 6). To further prove that such kind of compounds promoted immune activation through blockade of PD‐1/PD‐L1, a T‐cell recovery activity assay in Jurkat cells was done. Four tested compounds (**Y7f**, **Y7g**, **Y7q**, **Y7r**) counteracted the PD‐L1‐mediated inhibition of IFN‐γ secretion by T cells. (Figure 7). The superior performance of compound **Y7f** compared to other analogs (e.g., **Y7q** and **Y7r**) may be attributed to two key structural advantages. First, unlike compounds **Y7q** and **Y7r**, which contain readily hydrolysable ester bonds, compound **Y7f** lacks such hydrolytically labile groups, thereby maintaining its bioactivity without intracellular degradation via ester bond hydrolysis. Second, compound **Y7f** possesses a lower ALogP value, resulting in poorer membrane permeability. Consequently, a higher proportion of **Y7f** molecules remain extracellular where they efficiently block PD‐1/PD‐L1 protein interactions. MD simulations identify compound **(1R, 16R)‐Y7f** as the most potent PD‐L1 dimerization inducer within the compound **Y7f** stereoisomer series. This dual mechanism significantly enhances Jurkat T cell‐mediated cytotoxicity against PD‐L1‐overexpressing HepG2 cells, ultimately yielding superior immunotherapeutic efficacy. These findings further confirm that the novel biphenyl‐tetrahydroisoquinoline scaffold represents a promising structural framework for developing PD‐1/PD‐L1 inhibitors and warrants in‐depth exploration.

## 4 MATERIAL AND METHODS

### 4.1 Chemistry

All chemicals and organic reagents were purchased from regular manufacturers. Dichloromethane (DCM) was treated anhydrous and oxygen free and Raney nickel was purified without further treatment of chemicals and solvents except water: the synthesis reaction was carried out under specific temperature conditions and the products were separated by column chromatography to obtain pure compounds. ^1^H NMR and ^13^C NMR spectra were obtained on nuclear magnetic resonance spectrometer Vaian DLG400, Bruker Avance II 400, Bruker Avance NEO 600 and Bruker Avance III 500, with TMS as internal standard, chemical shifts (*δ*) in ppm, coupling constants (*J*) in hertz (Hz), and high‐resolution mass spectrometry (HRMS) data were measured on an Agilent G6230B instrument. Thin layer chromatography (TLC) separation was performed on pre‐coated silica gel plates.

Methyl 2‐(phenylthio)acetate (**Y1**): To a solution of thiophenol (21 mL, 0.205 mol, 1.0 equiv.) in toluene (180 mL) was added sodium metal (5.83 g, 0.254 mol, 1.25 equiv.) under a nitrogen atmosphere. The mixture was refluxed at 110°C with vigorous stirring until the reaction was confirmed to be complete by TLC analysis. Methyl chloroacetate (25 mL, 0.29 mol, 1.4 equiv.) was then added dropwise over 30 min, and the reaction was stirred for an additional 1 h at reflux temperature. After cooling to room temperature, the reaction mixture was partitioned between ethyl acetate and deionized water. The organic layer was separated, dried over anhydrous Na_2_SO_4_, filtered, and concentrated under reduced pressure to yield 40.17 g of a yellow oily liquid as the crude product. Purification by column chromatography (eluent: ethyl acetate/petroleum ether = 1:40, v/v) afforded 31.67 g of a pale yellow liquid, representing an 85% isolated yield. ^1^H NMR (600 MHz, Chloroform‐*d*) *δ* 7.39 (d, *J* = 7.8 Hz, 2H), 7.28 (t, *J* = 7.6 Hz, 2H), 7.21 (t, *J* = 7.6 Hz, 1H), 3.68 (s, 3H), 3.63 (s, 2H) (Figure S7).

Methyl 2‐chloro‐2‐(phenylthio)acetate (**Y2**): To a solution of compound **Y1** (31.67 g, 0.17 mol, 1.0 equiv.) in DCM (200 mL) at −10 to −20°C, sulfonyl chloride (17 mL, 0.204 mol, 1.2 equiv.) was added dropwise. The reaction mixture was stirred for 2 h after the addition, and the completion of the reaction was monitored by TLC. The reaction mixture was then poured into ice water and neutralized with 1 M NaOH solution. The aqueous layer was extracted with DCM, and the combined organic phases were dried over anhydrous Na_2_SO_4_, filtered, and concentrated under reduced pressure to afford 31.86 g of a brown oily liquid as the crude product. Purification by column chromatography (eluent: DCM/petroleum ether = 1:10, v/v) yielded 29.50 g of a pale yellow oily liquid, corresponding to a 78% isolated yield. ^1^H NMR (400 MHz, Chloroform‐*d*) *δ* 7.44 (dd, *J* = 7.8, 1.8 Hz, 2H), 7.28–7.22 (m, 3H), 5.41 (s, 1H), 3.65 (s, 3H) (Figure S8).

2‐([1,1′‐biphenyl]‐4‐yl)‐N‐(4‐methoxyphenethyl)acetamide (**Y3**): To a solution of 2‐(4‐methoxyphenyl)ethylamine hydrochloride (1.91 g, 10.18 mmol, 1.0 equiv.) and 4‐biphenylacetic acid (2.37 g, 11.21 mmol, 1.1 equiv.) in acetonitrile (120 mL) at room temperature was added triethylamine (5.09 mL, 36.65 mmol, 3.5 equiv.). The mixture was stirred for 15 min, followed by the addition of HBTU (5.0 g, 13.18 mmol, 1.3 equiv.). The reaction was allowed to proceed for 1.5 h, and completion was confirmed by TLC analysis. The solvent was removed under reduced pressure to afford the crude product, which was dissolved in DCM (100 mL) and sequentially washed with deionized water, saturated NaHCO_3_ solution, and saturated NaCl solution. The organic phase was dried over anhydrous Na_2_SO_4_, filtered, and concentrated under reduced pressure to yield 2.9 g of a white solid, representing a 95% isolated yield. ^1^H NMR (600 MHz, Chloroform‐*d*) *δ* 7.60 (d, *J* = 6.7 Hz, 2H), 7.55 (d, *J* = 8.1 Hz, 2H), 7.46 (t, *J* = 7.7 Hz, 2H), 7.36 (t, *J* = 7.4 Hz, 1H), 7.24 (d, *J* = 7.9 Hz, 2H), 6.94 (d, *J* = 8.5 Hz, 2H), 6.74 (d, *J* = 8.5 Hz, 2H), 5.38 (s, 1H), 3.69 (s, 3H), 3.57 (s, 2H), 3.45 (q, *J* = 6.5 Hz, 2H), 2.68 (t, *J* = 6.8 Hz, 2H) (Figure S9).

Methyl 2‐(5‐(2‐(2‐([1,1′‐biphenyl]‐4‐yl)acetamido)ethyl)‐2‐methoxyphenyl)‐2‐(phenylthio)acetate (**Y4**): To a solution of compound **Y3** (2.21 g, 6.41 mmol, 1.0 equiv.) in DCM (60 mL) was added a solution of anhydrous SnCl_4_ (1.3 mL, 6.41 mmol, 1.0 equiv.) in DCM dropwise. After stirring for 15 min, a solution of compound **Y2** in DCM was added dropwise to the reaction mixture, maintaining the temperature between 20 and 25°C. The reaction was stirred for an additional 15 min, and completion was confirmed by TLC analysis. The reaction mixture was then slowly poured into ice water (100 mL) and extracted with DCM. The organic phase was sequentially washed with deionized water, saturated NaHCO_3_ solution, and saturated NaCl solution, dried over anhydrous Na_2_SO_4_, filtered, and concentrated under reduced pressure to afford 4.00 g of the crude product. Purification by column chromatography (eluent: acetone/petroleum ether = 3.5:1, v/v) yielded 1.89 g of a clear oily compound, representing a 57% isolated yield. ^1^H NMR (400 MHz, Chloroform‐*d*) *δ* 7.58 (d, *J* = 7.3 Hz, 2H), 7.55 (d, *J* = 7.7 Hz, 2H), 7.44 (t, *J* = 7.5 Hz, 2H), 7.38–7.32 (m, 4H), 7.25–7.20 (m, 5H), 6.90 (d, *J* = 8.3 Hz, 1H), 6.67 (d, *J* = 8.2 Hz, 1H), 5.43 (s, 1H), 5.36 (s, 1H), 3.68 (s, 3H), 3.65 (s, 3H), 3.62 (q, 2H), 3.41 (s, 2H), 2.67 (t, *J* = 6.5 Hz, 2H) (Figure S10).

Methyl 2‐(5‐(2‐(2‐([1,1′‐biphenyl]‐4‐yl)acetamido)ethyl)‐2‐methoxyphenyl)acetate (**Y5**): To a solution of compound **Y4** (2.21 g, 4.2 mmol, 1.0 equiv.) in tetrahydrofuran (THF, 80 mL) was added Raney nickel (2.2 g). The reaction mixture was stirred at room temperature for 30 min, and completion was confirmed by TLC analysis. The reaction mixture was then filtered through a pad of Celite, and the filter cake was washed with DCM. The filtrate was concentrated under reduced pressure to afford 1.69 g of a white solid, representing a 96% isolated yield. ^1^H NMR (400 MHz, Chloroform‐*d*) *δ* 7.59 (d, *J* = 7.7 Hz, 2H), 7.55 (d, *J* = 7.9 Hz, 2H), 7.45 (t, *J* = 7.5 Hz, 2H), 7.39–7.33 (m, 1H), 7.27 (d, *J* = 6.8 Hz, 2H), 6.90 (d, *J* = 7.5 Hz, 2H), 6.68 (d, *J* = 8.1 Hz, 1H), 5.52 (s, 1H), 3.70 (s, 3H), 3.67 (s, 3H), 3.56 (d, *J* = 6.9 Hz, 4H), 3.48–3.41 (m, 2H), 2.68 (t, *J* = 6.9 Hz, 2H) (Figure S11).

Methyl 2‐(1‐([1,1′‐biphenyl]‐4‐ylmethyl)‐7‐methoxy‐1,2,3,4‐tetrahydroisoquinolin‐6‐yl)acetate (**Y6**): To a mixture of compound **Y5** (1.74 g, 4.17 mmol, 1.0 equiv.), ZnCl_2_ (1.69 g, 12.44 mmol, 3.0 equiv.), and POCl_3_ (31.1 mL, 340 mmol) was stirred and refluxed at 110°C. The reaction was complete after 30 min, as monitored by TLC. The POCl_3_ was removed under reduced pressure, and the residue was dissolved in DCM. Ice‐cold water was added, and the pH was adjusted to weakly basic using 1 M NaOH solution. The mixture was extracted, and the organic phase was sequentially washed with NaHCO_3_ solution and saturated NaCl solution, dried over anhydrous Na_2_SO_4_, filtered, and concentrated under reduced pressure to yield 1.74 g of a purple‐red solid.

The solid was then dissolved in a mixture of DCM (10 mL) and methanol (10 mL). Sodium borohydride (0.14 g, 3.87 mmol, 1.5 equiv.) was added at 0°C, and the reaction was stirred for 30 min. Completion was confirmed by TLC. The pH of the reaction mixture was adjusted to weakly acidic using 2.0 M HCl solution, and the organic solvents were removed under reduced pressure. The residue was dissolved in DCM and sequentially washed with saturated NaHCO_3_ solution and saturated NaCl solution. The organic phase was dried over anhydrous Na_2_SO_4_, filtered, and concentrated under reduced pressure to afford 1.00 g of a yellow solid. Purification by column chromatography (eluent: petroleum ether/ethyl acetate/triethylamine = 2:1:1%, v/v/v) yielded 0.82 g of a pale yellow solid, representing a 40% isolated yield. ^1^H NMR (400 MHz, Chloroform‐*d*) *δ* 7.58 (t, *J* = 8.0 Hz, 4H), 7.44 (t, *J* = 7.5 Hz, 2H), 7.37–7.34 (m, 3H), 6.93 (s, 1H), 6.61 (s, 1H), 4.28 (dd, 1H), 3.73 (s, 3H), 3.70 (s, 3H), 3.60 (d, *J* = 2.3 Hz, 2H), 3.28 (d, *J* = 4.6 Hz, 1H), 3.26–3.23 (m, 1H), 3.05 (d, *J* = 4.3 Hz, 1H), 3.00–2.95 (m, 1H), 2.77 (q, *J* = 6.3 Hz, 2H) (Figure S12).

2‐(1‐([1,1′‐biphenyl]‐4‐ylmethyl)‐7‐methoxy‐1,2,3,4‐tetrahydroisoquinolin‐6‐yl)acetic acid (**Y7**): To a solution of compound **Y6** (60 mg, 0.14 mmol, 1.0 equiv.) in methanol was added 1 M aqueous NaOH solution to adjust the pH to 12. After stirring for 3 h, the reaction mixture was acidified to pH 2–3 using 1 M HCl solution, resulting in the formation of a white solid. The solid was collected by filtration to afford 30 mg of the product as a white solid, representing a 55% isolated yield. ^1^H NMR (600 MHz, DMSO‐*d*_6_) *δ* 7.66 (d, *J* = 7.7 Hz, 2H), 7.61 (d, *J* = 7.8 Hz, 2H), 7.49–7.40 (m, 4H), 7.35 (t, *J* = 7.4 Hz, 1H), 6.87 (s, 1H), 6.78 (s, 1H), 4.14 (dd, *J* = 9.5, 4.4 Hz, 1H), 3.68 (s, 3H), 3.43 (s, 2H), 3.22 (dd, *J* = 13.6, 4.4 Hz, 1H), 3.12 (dt, *J* = 12.1, 5.9 Hz, 1H), 2.90 (dd, *J* = 13.6, 9.4 Hz, 1H), 2.82 (dt, *J* = 11.8, 5.7 Hz, 1H), 2.62 (ddt, *J* = 19.4, 13.1, 5.8 Hz, 2H) (Figure S13). ^13^C NMR (101 MHz, DMSO) *δ* 172.27, 155.36, 139.88, 138.75, 136.19, 132.60, 130.46, 128.96, 126.77, 126.53, 123.79, 123.17, 108.85, 55.41, 55.25, 40.15, 38.78, 35.08, 24.55 (Figure S14). HRMS (ESI Positive) m/z: calcd for C_25_H_26_NO_3_ [M + H]^+^: 388.1834; found: 388.1907.

Synthesis process of compounds **Y7a–Y7g**, **Y7i–Y7p**: To a solution of compound **Y7** (0.2 mmol, 1.0 equiv.), the amine compound (0.3 mmol, 1.5 equiv.), and HBTU (0.3 mmol, 1.5 equiv.) in DCM was stirred at room temperature for 12 h. The completion of the reaction was confirmed by TLC analysis. The reaction mixture was sequentially washed with deionized water, saturated NaHCO_3_ solution, and saturated NaCl solution. The organic phase was dried over anhydrous Na_2_SO_4_, filtered, and concentrated under reduced pressure to afford the crude product. Purification by silica gel column chromatography yielded the pure compounds **Y7a**–**Y7g** and **Y7i**–**Y7p**.

2‐(1‐([1,1′‐biphenyl]‐4‐ylmethyl)‐7‐methoxy‐1,2,3,4‐tetrahydroisoquinolin‐6‐yl)‐1‐(4‐hydroxypiperidin‐1‐yl)ethan‐1‐one (**Y7a**): Yield 58%, white solid. ^1^H NMR (400 MHz, DMSO‐*d*_6_) *δ* 7.66 (d, *J* = 7.2 Hz, 2H), 7.62 (d, *J* = 8.1 Hz, 2H), 7.44 (dt, *J* = 13.1, 7.0 Hz, 4H), 7.38–7.32 (m, 1H), 6.81 (s, 1H), 6.75 (s, 1H), 4.74 (s, 1H), 4.21 (dd, *J* = 9.2, 4.6 Hz, 1H), 3.92 (dt, *J* = 10.4, 4.7 Hz, 1H), 3.67 (s, 5H), 3.52 (dd, *J* = 8.0, 3.4 Hz, 2H), 3.24 (dd, *J* = 13.6, 4.7 Hz, 1H), 3.14 (dq, *J* = 12.4, 4.8, 4.2 Hz, 2H), 2.97 (tt, *J* = 13.5, 6.1 Hz, 2H), 2.86 (dt, *J* = 11.9, 5.6 Hz, 1H), 2.63 (q, *J* = 6.5 Hz, 2H), 1.74–1.61 (m, 2H), 1.30–1.17 (m, 2H) (Figure S15). ^13^C NMR (101 MHz, DMSO) *δ* 168.59, 154.60, 140.10, 138.62, 138.02, 137.19, 130.45, 130.18, 128.91, 127.21, 126.50, 126.47, 122.47, 108.77, 65.63, 56.49, 55.35, 42.95, 41.21, 40.15, 34.59, 33.97, 33.50, 27.75 (Figure S16). HRMS (ESI Positive) m/z: calcd for C_30_H_35_N_2_O_3_ [M + H]^+^: 471.2569; found: 471.2646.

1‐(2‐(1‐([1,1′‐biphenyl]‐4‐ylmethyl)‐7‐methoxy‐1,2,3,4‐tetrahydroisoquinolin‐6‐yl)acetyl)piperidine‐4‐carboxamide (**Y7b**): Yield 68%, white solid. ^1^H NMR (400 MHz, DMSO‐*d*_6_) *δ* 7.66 (d, *J* = 7.4 Hz, 2H), 7.62 (d, *J* = 8.1 Hz, 2H), 7.47 (d, *J* = 7.4 Hz, 2H), 7.45–7.40 (m, 2H), 7.35 (t, *J* = 7.3 Hz, 1H), 7.28 (s, 1H), 6.82 (s, 1H), 6.78 (s, 1H), 6.74 (d, *J* = 12.0 Hz, 1H), 4.34 (d, *J* = 13.1 Hz, 1H), 4.21 (dd, *J* = 9.2, 4.7 Hz, 1H), 3.91 (d, *J* = 13.5 Hz, 1H), 3.67 (d, *J* = 5.6 Hz, 3H), 3.57 (dd, *J* = 15.5, 5.8 Hz, 1H), 3.48 (dd, *J* = 15.4, 4.9 Hz, 1H), 3.24 (dd, *J* = 13.6, 4.6 Hz, 1H), 3.15 (dt, *J* = 12.2, 6.0 Hz, 1H), 3.05–2.91 (m, 2H), 2.86 (dt, *J* = 11.8, 5.5 Hz, 1H), 2.70–2.55 (m, 3H), 2.38–2.28 (m, 1H), 1.68 (d, *J* = 12.9 Hz, 2H), 1.46–1.29 (m, 2H) (Figure S17). ^13^C NMR (101 MHz, DMSO) *δ* 175.97, 168.64, 154.67, 140.10, 138.53, 138.06, 136.99, 130.57, 130.21, 128.93, 127.22, 126.51, 125.94, 122.53, 108.77, 56.44, 55.32, 44.99, 41.39, 41.13, 40.90, 40.15, 33.51, 28.77, 28.19, 27.60 (Figure S18). HRMS (ESI Positive) m/z: calcd for C_31_H_35_N_3_O_3_Na [M + Na]^+^: 520.2678; found: 520.2605.

2‐(1‐([1,1′‐biphenyl]‐4‐ylmethyl)‐7‐methoxy‐1,2,3,4‐tetrahydroisoquinolin‐6‐yl)‐1‐(piperazin‐1‐yl) ethan‐1‐one (**Y7c**): Yield 74%, white solid. ^1^H NMR (400 MHz, DMSO‐*d*_6_) *δ* 7.66 (d, *J* = 7.3 Hz, 2H), 7.60 (d, *J* = 7.9 Hz, 2H), 7.49–7.38 (m, 4H), 7.35 (t, *J* = 7.3 Hz, 1H), 6.78 (d, *J* = 2.0 Hz, 2H), 4.09 (dd, *J* = 9.5, 4.2 Hz, 1H), 3.69 (s, 3H), 3.50 (s, 2H), 3.36 (s, 4H), 3.20 (dd, *J* = 13.5, 4.3 Hz, 1H), 3.10 (dt, *J* = 12.0, 5.9 Hz, 1H), 2.87 (dd, *J* = 13.5, 9.5 Hz, 1H), 2.78 (dt, *J* = 11.9, 5.6 Hz, 1H), 2.58 (p, *J* = 7.2, 6.8 Hz, 6H) (Figure S19). ^13^C NMR (101 MHz, DMSO) *δ* 168.83, 154.52, 140.16, 139.28, 138.45, 137.82, 130.45, 130.12, 128.90, 126.65, 126.49, 126.38, 121.97, 108.74, 56.75, 55.37, 46.76, 45.87, 45.48, 42.45, 41.71, 33.47, 28.55 (Figure S20). HRMS (ESI Positive) m/z: calcd for C_29_H_33_N_3_O_2_Na [M + Na]^+^: 478.2573; found: 478.2492.

2‐(1‐([1,1′‐biphenyl]‐4‐ylmethyl)‐7‐methoxy‐1,2,3,4‐tetrahydroisoquinolin‐6‐yl)‐1‐(4‐acetylpiperazin‐1‐yl)ethan‐1‐one (**Y7d**): Yield 61%, white solid. ^1^H NMR (400 MHz, DMSO‐*d*_6_) *δ* 7.66 (d, *J* = 7.5 Hz, 2H), 7.62 (d, *J* = 7.9 Hz, 2H), 7.50–7.38 (m, 4H), 7.36 (d, *J* = 7.4 Hz, 1H), 6.83 (s, 1H), 6.77 (s, 1H), 4.19 (dd, *J* = 9.2, 4.5 Hz, 1H), 3.68 (s, 3H), 3.57 (s, 2H), 3.50 (s, 4H), 3.34 (s, 4H), 3.24 (dd, *J* = 13.6, 4.5 Hz, 1H), 3.15 (dt, *J* = 12.1, 5.9 Hz, 1H), 2.93 (dd, *J* = 13.6, 9.3 Hz, 1H), 2.88–2.79 (m, 1H), 2.63 (q, *J* = 7.0, 6.4 Hz, 2H), 2.01 (s, 3H) (Figure S21).^13^C NMR (101 MHz, DMSO) *δ* 169.57, 168.95, 155.21, 140.54, 138.63, 131.13, 130.69, 129.40, 127.72, 126.99, 122.94, 109.30, 56.82, 55.84, 55.39, 45.88, 41.39, 40.65, 33.96, 27.76, 21.73 (Figure S22). HRMS (ESI Positive) m/z: calcd for C_31_H_36_N_3_O_3_ [M + H]^+^: 498.2678; found: 498.2753.

2‐(1‐([1,1′‐biphenyl]‐4‐ylmethyl)‐7‐methoxy‐1,2,3,4‐tetrahydroisoquinolin‐6‐yl)‐1‐morpholinoethan‐1‐one (**Y7e**): Yield 65%, white solid. ^1^H NMR (400 MHz, DMSO‐*d*_6_) *δ* 7.66 (d, *J* = 7.4 Hz, 2H), 7.61 (d, *J* = 8.0 Hz, 2H), 7.49–7.39 (m, 4H), 7.35 (t, *J* = 7.3 Hz, 1H), 6.82 (s, 1H), 6.77 (s, 1H), 4.18 (dd, *J* = 9.4, 4.4 Hz, 1H), 3.68 (s, 3H), 3.53 (s, 6H), 3.46 (dd, *J* = 10.2, 4.5 Hz, 4H), 3.23 (dd, *J* = 13.6, 4.5 Hz, 1H), 3.18–3.11 (m, 1H), 2.92 (dd, *J* = 13.5, 9.4 Hz, 1H), 2.84 (dt, *J* = 11.9, 5.6 Hz, 1H), 2.62 (q, *J* = 6.4 Hz, 2H) (Figure S23). ^13^C NMR (101 MHz, DMSO) *δ* 169.12, 154.65, 140.12, 138.86, 137.96, 137.73, 130.66, 130.16, 128.91, 127.19, 126.50, 126.31, 108.76, 66.14, 56.59, 55.36, 45.87, 41.69, 41.37, 33.30, 28.03 (Figure S24). HRMS (ESI Positive) m/z: calcd for C_29_H_33_N_2_O_3_ [M + H]^+^: 457.2413; found: 457.2490.

2‐(1‐([1,1′‐biphenyl]‐4‐ylmethyl)‐7‐methoxy‐1,2,3,4‐tetrahydroisoquinolin‐6‐yl)‐1‐(3‐hydroxypyrro‐lidin‐1‐yl)ethan‐1‐one (**Y7f**): Yield 83%, white solid. ^1^H NMR (400 MHz, DMSO‐*d*_6_) *δ* 7.66 (d, *J* = 7.4 Hz, 2H), 7.62 (d, *J* = 8.2 Hz, 2H), 7.47 (d, *J* = 7.5 Hz, 2H), 7.44–7.39 (m, 2H), 7.35 (t, *J* = 7.3 Hz, 1H), 6.83 (s, 1H), 6.76 (s, 1H), 4.96 (d, *J* = 39.5 Hz, 1H), 4.28 (d, *J* = 28.6 Hz, 1H), 4.17 (dd, *J* = 9.4, 4.4 Hz, 1H), 3.67 (s, 3H), 3.55 (pd, *J* = 5.3, 4.9, 1.7 Hz, 2H), 3.47 (s, 1H), 3.43 (s, 1H), 3.40 (q, *J* = 4.8, 3.9 Hz, 1H), 3.30–3.27 (m, 1H), 3.23 (dd, *J* = 13.6, 4.4 Hz, 1H), 3.14 (ddd, *J* = 12.0, 6.8, 5.1 Hz, 1H), 2.91 (dd, *J* = 13.6, 9.4 Hz, 1H), 2.87–2.79 (m, 1H), 2.63 (dq, *J* = 13.3, 6.8, 6.0 Hz, 2H), 2.00–1.68 (m, 2H) (Figure S25).^13^C NMR (101 MHz, DMSO) *δ* 168.76, 154.98, 140.13, 138.89, 137.95, 137.63, 131.00, 130.15, 128.92, 127.19, 126.50, 126.19, 108.69, 69.60, 56.62, 55.37, 54.54, 53.84, 43.52, 41.41, 34.03, 32.48, 28.08 (Figure S26). HRMS (ESI Positive) m/z: calcd for C_29_H_33_N_2_O_3_ [M + H]^+^: 457.2413; found: 457.2487.

2‐(1‐([1,1′‐biphenyl]‐4‐ylmethyl)‐7‐methoxy‐1,2,3,4‐tetrahydroisoquinolin‐6‐yl)‐1‐(3‐hydroxy‐3‐met‐hylpyrrolidin‐1‐yl)ethan‐1‐one (**Y7g**): Yield 80%, white solid. ^1^H NMR (400 MHz, DMSO‐*d*_6_) *δ* 7.66 (d, *J* = 7.1 Hz, 2H), 7.61 (d, *J* = 8.0 Hz, 2H), 7.49–7.40 (m, 4H), 7.35 (t, *J* = 7.4 Hz, 1H), 6.82 (s, 1H), 6.77 (s, 1H), 4.79 (dd, *J* = 34.4, 2.2 Hz, 1H), 4.15 (dd, *J* = 9.5, 4.3 Hz, 1H), 3.68 (s, 3H), 3.58 (dd, *J* = 8.7, 5.5 Hz, 1H), 3.45 (d, *J* = 7.6 Hz, 1H), 3.40 (s, 2H), 3.38–3.33 (m, 2H), 3.22 (dd, *J* = 13.6, 4.4 Hz, 1H), 3.16–3.08 (m, 1H), 2.94–2.86 (m, 1H), 2.82 (dt, *J* = 11.9, 5.6 Hz, 1H), 2.60 (q, *J* = 6.8, 6.2 Hz, 2H), 1.88–1.67 (m, 2H), 1.28 (d, *J* = 3.5 Hz, 3H) (Figure S27). ^13^C NMR (101 MHz, DMSO) *δ* 168.61, 154.95, 154.92, 140.14, 139.00, 137.92, 137.80, 130.15, 128.91, 127.19, 126.50, 126.27, 108.67, 75.70, 58.21, 56.66, 55.38, 45.38, 41.48, 37.75, 34.91, 28.20, 25.39 (Figure S28). HRMS (ESI Positive) m/z: calcd for C_30_H_34_N_2_O_3_Na [M + Na]^+^: 493.2569; found: 493.2476.

2‐(1‐([1,1′‐biphenyl]‐4‐ylmethyl)‐7‐methoxy‐1,2,3,4‐tetrahydroisoquinolin‐6‐yl)‐N‐(2‐hydroxyethyl) acetamide (**Y7i**): Yield 65%, white solid. ^1^H NMR (400 MHz, DMSO‐*d*_6_) *δ* 7.74 (t, *J* = 5.6 Hz, 1H), 7.66 (d, *J* = 7.1 Hz, 2H), 7.62 (d, *J* = 7.9 Hz, 2H), 7.49–7.39 (m, 4H), 7.35 (t, *J* = 7.3 Hz, 1H), 6.88 (s, 1H), 6.74 (s, 1H), 4.66 (s, 1H), 4.21 (dd, *J* = 9.3, 4.6 Hz, 1H), 3.66 (s, 3H), 3.39 (d, *J* = 7.1 Hz, 4H), 3.24 (dd, *J* = 13.6, 4.6 Hz, 1H), 3.14 (dq, *J* = 12.1, 6.2 Hz, 3H), 2.94 (dd, *J* = 13.6, 9.3 Hz, 1H), 2.86 (dd, *J* = 12.2, 6.0 Hz, 1H), 2.64 (q, *J* = 6.6 Hz, 2H) (Figure S31). ^13^C NMR (101 MHz, DMSO) *δ* 170.23, 155.04, 140.11, 138.70, 138.01, 137.28, 130.97, 130.18, 128.91, 127.20, 126.50, 126.47, 126.04, 122.72, 108.81, 59.92, 56.53, 55.35, 41.59, 41.27, 39.61, 36.40, 27.86 (Figure S32). HRMS (ESI Positive) m/z: calcd for C_27_H_31_N_2_O_3_ [M + H]^+^: 431.2256; found: 431.2333.

2‐(1‐([1,1′‐biphenyl]‐4‐ylmethyl)‐7‐methoxy‐1,2,3,4‐tetrahydroisoquinolin‐6‐yl)‐N‐(1,3‐dihydroxypro‐pan‐2‐yl)acetamide (**Y7j**): Yield 73%, white solid. ^1^H NMR (400 MHz, DMSO‐*d*_6_) *δ* 7.66 (d, *J* = 7.1 Hz, 2H), 7.62 (d, *J* = 7.9 Hz, 2H), 7.50–7.39 (m, 5H), 7.35 (t, *J* = 7.3 Hz, 1H), 6.89 (s, 1H), 6.72 (s, 1H), 4.64 (s, 2H), 4.21 (dd, *J* = 8.9, 4.6 Hz, 1H), 3.75–3.68 (m, 1H), 3.66 (s, 3H), 3.40 (h, *J* = 5.9 Hz, 6H), 3.23 (dd, *J* = 13.6, 4.6 Hz, 1H), 3.15 (dt, *J* = 12.2, 5.9 Hz, 1H), 2.95 (dd, *J* = 13.6, 9.1 Hz, 1H), 2.86 (dt, *J* = 11.9, 5.6 Hz, 1H), 2.72–2.55 (m, 2H) (Figure S33).^13^C NMR (101 MHz, DMSO) *δ* 170.10, 155.04, 140.11, 138.59, 138.04, 136.99, 130.91, 130.22, 128.93, 127.22, 126.51, 125.95, 122.93, 108.81, 59.95, 56.46, 55.30, 52.81, 41.17, 40.15, 36.63, 27.70 (Figure S34). HRMS (ESI Positive) m/z: calcd for C_28_H_33_N_2_O_4_ [M + H]^+^: 461.2362; found: 461.2436.

2‐(1‐([1,1′‐biphenyl]‐4‐ylmethyl)‐7‐methoxy‐1,2,3,4‐tetrahydroisoquinolin‐6‐yl)‐N‐(1,3‐dihydroxy‐2‐methylpropan‐2‐yl)acetamide (**Y7k**): Yield 66%, white solid. ^1^H NMR (400 MHz, DMSO‐*d*_6_) *δ* 7.66 (d, *J* = 7.7 Hz, 2H), 7.60 (d, *J* = 7.7 Hz, 2H), 7.46 (t, *J* = 7.5 Hz, 2H), 7.41 (d, *J* = 7.8 Hz, 2H), 7.34 (t, *J* = 7.3 Hz, 1H), 7.15 (s, 1H), 6.86 (s, 1H), 6.77 (s, 1H), 4.80 (t, *J* = 5.7 Hz, 2H), 4.09 (dd, *J* = 9.7, 4.1 Hz, 1H), 3.69 (s, 3H), 3.47 (dd, *J* = 10.8, 5.6 Hz, 2H), 3.39 (dd, *J* = 10.8, 5.8 Hz, 2H), 3.32 (s, 2H), 3.19 (dd, *J* = 13.6, 4.2 Hz, 1H), 3.10 (dt, *J* = 12.1, 6.0 Hz, 1H), 2.87 (dd, *J* = 13.5, 9.5 Hz, 1H), 2.78 (dt, *J* = 11.9, 5.6 Hz, 1H), 2.58 (q, *J* = 6.3, 5.9 Hz, 2H), 1.23 (s, 1H), 1.13 (s, 3H) (Figure S35). ^13^C NMR (101 MHz, DMSO) *δ* 170.81, 155.01, 140.10, 138.52, 138.06, 137.04, 130.97, 130.18, 128.92, 127.22, 126.50, 125.95, 122.94, 108.83, 63.94, 58.25, 56.47, 55.30, 41.17, 40.15, 37.48, 27.69, 18.60 (Figure S36). HRMS (ESI Positive) m/z: calcd for C_29_H_34_N_2_O_4_Na [M + Na]^+^: 497.2519; found: 497.2413.

2‐(1‐([1,1′‐biphenyl]‐4‐ylmethyl)‐7‐methoxy‐1,2,3,4‐tetrahydroisoquinolin‐6‐yl)‐N‐(1,3‐dihydroxy‐2‐(hydroxymethyl)propan‐2‐yl)acetamide (**Y7l**): Yield 58%, white solid. ^1^H NMR (400 MHz, DMSO‐*d*_6_) *δ* 7.66 (d, *J* = 7.7 Hz, 2H), 7.60 (d, *J* = 7.7 Hz, 2H), 7.46 (t, *J* = 7.5 Hz, 2H), 7.41 (d, *J* = 7.8 Hz, 2H), 7.35 (t, *J* = 7.5 Hz, 1H), 7.12 (s, 1H), 6.88 (s, 1H), 6.78 (s, 1H), 4.77 (t, *J* = 5.8 Hz, 3H), 4.09 (dd, *J* = 9.9, 4.0 Hz, 1H), 3.70 (d, *J* = 1.5 Hz, 3H), 3.51 (d, *J* = 5.4 Hz, 6H), 3.38 (s, 2H), 3.19 (dd, *J* = 13.5, 4.1 Hz, 1H), 3.14–3.04 (m, 1H), 2.87 (dd, *J* = 13.4, 9.6 Hz, 1H), 2.78 (dt, *J* = 11.8, 5.7 Hz, 1H), 2.58 (d, *J* = 5.8 Hz, 2H), 1.23 (s, 1H) (Figure S37). ^13^C NMR (126 MHz, DMSO) *δ* 171.70, 154.85, 140.13, 139.22, 138.66, 137.78, 130.99, 130.04, 128.82, 127.07, 126.75, 126.42, 126.32, 122.15, 108.85, 61.99, 60.60, 56.70, 55.30, 41.74, 40.04, 37.53, 28.55 (Figure S38). HRMS (ESI Positive) m/z: calcd for C_29_H_34_N_2_O_5_Na [M + Na]^+^: 513.2468; found: 513.2371.

2‐(1‐([1,1′‐biphenyl]‐4‐ylmethyl)‐7‐methoxy‐1,2,3,4‐tetrahydroisoquinolin‐6‐yl)‐N‐(2‐hydroxy‐2‐methylpropyl)acetamide (**Y7m**): Yield 50%, white solid. ^1^H NMR (400 MHz, DMSO‐*d*_6_) *δ* 7.66 (d, *J* = 7.1 Hz, 2H), 7.63 (d, *J* = 8.2 Hz, 2H), 7.55–7.50 (m, 1H), 7.46 (t, *J* = 7.7 Hz, 2H), 7.42 (d, *J* = 8.1 Hz, 2H), 7.35 (t, *J* = 7.3 Hz, 1H), 6.90 (s, 1H), 6.73 (s, 1H), 4.44 (s, 1H), 4.24 (dd, *J* = 9.4, 4.8 Hz, 1H), 3.66 (s, 3H), 3.38 (s, 2H), 3.25 (dd, *J* = 13.7, 4.6 Hz, 1H), 3.21–3.14 (m, 1H), 3.02 (d, *J* = 6.0 Hz, 2H), 2.98–2.93 (m, 1H), 2.90 (dd, *J* = 11.9, 5.7 Hz, 1H), 2.66 (t, *J* = 5.7 Hz, 2H), 1.23 (s, 1H), 1.04 (s, 6H) (Figure S39). ^13^C NMR (101 MHz, DMSO) *δ* 170.34, 155.01, 140.11, 138.63, 138.03, 137.29, 131.13, 130.18, 128.92, 127.22, 126.50, 126.00, 122.79, 108.71, 69.38, 56.53, 55.30, 49.75, 41.26, 40.15, 36.74, 27.83, 27.17 (Figure S40). HRMS (ESI Positive) m/z: calcd for C_29_H_35_N_2_O_3_ [M + H]^+^: 459.2569; found: 459.2649.

2‐(1‐([1,1′‐biphenyl]‐4‐ylmethyl)‐7‐methoxy‐1,2,3,4‐tetrahydroisoquinolin‐6‐yl)‐N‐(2,3‐dihydroxypro‐pyl)‐N‐methylacetamide (**Y7n**): Yield 72%, white solid. ^1^H NMR (500 MHz, Methanol‐*d*_4_) *δ* 7.60 (d, *J* = 7.7 Hz, 4H), 7.42 (t, *J* = 7.6 Hz, 2H), 7.35 (dd, *J* = 13.9, 7.6 Hz, 3H), 6.93 (d, *J* = 8.4 Hz, 1H), 6.58 (s, 1H), 4.34 (t, *J* = 7.2 Hz, 1H), 3.85 (dd, *J* = 6.9, 4.6 Hz, 2H), 3.65 (s, 3H), 3.60–3.55 (m, 1H), 3.52 (d, *J* = 5.1 Hz, 1H), 3.49–3.46 (m, 1H), 3.43–3.37 (m, 1H), 3.34–3.24 (m, 3H), 3.14 (s, 2H), 3.10–3.05 (m, 1H), 3.01–2.94 (m, 2H), 2.80 (q, *J* = 7.1, 6.5 Hz, 2H) (Figure S41). ^13^C NMR (126 MHz, MeOD) *δ* 174.88, 156.62, 142.04, 141.01, 138.64, 137.42, 132.06, 131.23, 129.90, 128.32, 127.83, 126.98, 123.95, 109.83, 71.62, 64.93, 58.18, 55.84, 52.28, 42.58, 40.93, 38.27, 35.54, 28.44 (Figure S42). HRMS (ESI Positive) m/z: calcd for C_29_H_35_N_2_O_4_ [M + H]^+^: 475.2519; found: 475.2597.

2‐(1‐([1,1′‐biphenyl]‐4‐ylmethyl)‐7‐methoxy‐1,2,3,4‐tetrahydroisoquinolin‐6‐yl)‐N‐(2‐(dimethylamino) ethyl)acetamide (**Y7o**): Yield 61%, white solid. ^1^H NMR (400 MHz, DMSO‐*d*_6_) *δ* 7.70–7.64 (m, 3H), 7.62 (d, *J* = 8.2 Hz, 2H), 7.49–7.41 (m, 4H), 7.35 (t, *J* = 7.3 Hz, 1H), 6.89 (s, 1H), 6.74 (s, 1H), 4.21 (dd, *J* = 9.3, 4.7 Hz, 1H), 3.66 (s, 3H), 3.31 (s, 2H), 3.24 (dd, *J* = 13.6, 4.7 Hz, 1H), 3.15 (p, *J* = 6.4 Hz, 3H), 2.98–2.92 (m, 1H), 2.87 (td, *J* = 11.9, 11.2, 5.3 Hz, 1H), 2.64 (q, *J* = 7.2, 6.6 Hz, 2H), 2.30 (t, *J* = 6.6 Hz, 2H), 2.16 (s, 6H) (Figure S43). ^13^C NMR (101 MHz, DMSO) *δ* 170.04, 154.99, 140.11, 138.66, 138.03, 137.24, 130.94, 130.19, 128.93, 127.22, 126.51, 126.49, 126.02, 122.72, 108.76, 58.09, 56.52, 55.31, 45.07, 41.27, 40.15, 36.71, 36.53, 27.84 (Figure S44). HRMS (ESI Positive) m/z: calcd for C_29_H_36_N_3_O_2_ [M + H]^+^: 458.2729; found: 458.2814.

2‐(1‐([1,1′‐biphenyl]‐4‐ylmethyl)‐7‐methoxy‐1,2,3,4‐tetrahydroisoquinolin‐6‐yl)‐N‐(2‐acetamidoethyl) acetamide (**Y7p**): Yield 72%, white solid. ^1^H NMR (400 MHz, DMSO‐*d*_6_) *δ* 7.88 (s, 1H), 7.83 (s, 1H), 7.66 (d, *J* = 6.9 Hz, 2H), 7.62 (d, *J* = 8.0 Hz, 2H), 7.49–7.39 (m, 4H), 7.35 (t, *J* = 7.3 Hz, 1H), 6.87 (s, 1H), 6.75 (s, 1H), 4.18 (dd, *J* = 9.3, 4.5 Hz, 1H), 3.67 (s, 3H), 3.32 (s, 2H), 3.23 (dd, *J* = 13.6, 4.5 Hz, 1H), 3.16 (dd, *J* = 6.9, 5.3 Hz, 1H), 3.09 (q, *J* = 2.9 Hz, 4H), 2.93 (dd, *J* = 13.6, 9.3 Hz, 1H), 2.88–2.80 (m, 1H), 2.63 (d, *J* = 7.4 Hz, 2H), 1.79 (s, 3H) (Figure S45).^13^C NMR (101 MHz, DMSO) *δ* 170.33, 169.32, 155.02, 140.13, 138.87, 137.96, 137.65, 130.99, 130.17, 128.92, 127.20, 126.51, 126.46, 126.21, 122.48, 108.79, 56.61, 55.35, 41.41, 40.15, 38.53, 38.43, 36.42, 28.08, 22.65 (Figure S46). HRMS (ESI Positive) m/z: calcd for C_29_H_34_N_3_O_3_ [M + H]^+^: 472.2522; found: 472.2600.

Synthesis process of compounds **Y7h**, **Y7q–Y7r**: To a solution of compound **Y7** (0.28 mmol, 1.0 equiv.), the amine hydrochloride (1.10 mmol, 4.0 equiv.), and HBTU (0.37 mmol, 1.3 equiv.) in DCM was stirred at room temperature for 12 h. The completion of the reaction was confirmed by TLC analysis. The reaction mixture was sequentially washed with deionized water, saturated NaHCO_3_ solution, and saturated NaCl solution. The organic phase was dried over anhydrous Na_2_SO_4_, filtered, and concentrated under reduced pressure to afford the crude product. Purification by silica gel column chromatography yielded the pure compounds **Y7h**, **Y7q**–**Y7r**.

Methyl (2R,4R)‐1‐(2‐(1‐([1,1′‐biphenyl]‐4‐ylmethyl)‐7‐methoxy‐1,2,3,4‐tetrahydroisoquinolin‐6‐yl) acetyl)‐4‐hydroxypyrrolidine‐2‐carboxylate (**Y7h**): Yield 61%, white solid. ^1^H NMR (400 MHz, DMSO‐*d*_6_) *δ* 7.66 (d, *J* = 7.3 Hz, 2H), 7.62 (d, *J* = 8.1 Hz, 2H), 7.49–7.40 (m, 4H), 7.35 (t, *J* = 7.3 Hz, 1H), 6.91 (s, 1H), 6.76 (s, 1H), 5.12 (s, 1H), 4.36 (dd, *J* = 8.8, 5.3 Hz, 1H), 4.30 (q, *J* = 4.8 Hz, 1H), 4.21 (d, *J* = 10.2 Hz, 1H), 3.79 (dd, *J* = 10.4, 5.7 Hz, 1H), 3.71–3.56 (m, 6H), 3.49 (dd, *J* = 7.1, 3.5 Hz, 2H), 3.28–3.21 (m, 2H), 3.16 (dt, *J* = 12.1, 6.0 Hz, 1H), 2.94 (dd, *J* = 13.5, 9.3 Hz, 1H), 2.87 (q, *J* = 6.4 Hz, 1H), 2.65 (t, *J* = 6.8 Hz, 2H), 2.39–2.17 (m, 1H), 1.81 (dt, *J* = 11.8, 5.2 Hz, 1H) (Figure S29). ^13^C NMR (101 MHz, DMSO) *δ* 172.00, 168.98, 155.06, 139.99, 138.39, 137.43, 135.00, 130.29, 128.93, 127.29, 126.63, 126.51, 124.96, 122.52, 108.78, 68.74, 56.98, 56.03, 55.38, 54.35, 51.63, 40.30, 40.15, 37.21, 34.65, 26.39 (Figure S30). HRMS (ESI Positive) m/z: calcd for C_31_H_35_N_2_O_5_ [M + H]^+^: 515.2468; found: 515.2537.

Methyl (2‐(1‐([1,1′‐biphenyl]‐4‐ylmethyl)‐7‐methoxy‐1,2,3,4‐tetrahydroisoquinolin‐6‐yl)acetyl)‐L‐alaninate (**Y7q**): Yield 59%, white solid. ^1^H NMR (400 MHz, DMSO‐*d*_6_) *δ* 8.30 (d, *J* = 7.0 Hz, 1H), 7.66 (d, *J* = 7.2 Hz, 2H), 7.61 (d, *J* = 7.9 Hz, 2H), 7.43 (dd, *J* = 13.3, 7.8 Hz, 4H), 7.35 (t, *J* = 7.4 Hz, 1H), 6.88 (s, 1H), 6.75 (s, 1H), 4.27 (p, *J* = 7.2 Hz, 1H), 4.16 (dd, *J* = 9.3, 4.4 Hz, 1H), 3.67 (s, 3H), 3.62 (s, 3H), 3.36 (s, 2H), 3.22 (dd, *J* = 13.6, 4.4 Hz, 1H), 3.13 (dt, *J* = 12.1, 5.9 Hz, 1H), 2.90 (dd, *J* = 13.5, 9.4 Hz, 1H), 2.86–2.80 (m, 1H), 2.61 (q, *J* = 6.7, 6.2 Hz, 2H), 1.28 (d, *J* = 7.3 Hz, 3H) (Figure S47). ^13^C NMR (101 MHz, DMSO) *δ* 173.18, 170.02, 155.07, 140.03, 138.21, 137.98, 136.03, 130.83, 130.21, 128.91, 127.24, 126.55, 126.49, 125.37, 122.84, 108.76, 56.26, 55.32, 51.78, 47.61, 40.77, 39.40, 35.78, 27.09, 17.04 (Figure S48). HRMS (ESI Positive) m/z: calcd for C_29_H_33_N_2_O_4_ [M + H]^+^: 473.2362; found: 473.2441.

Methyl (2‐(1‐([1,1′‐biphenyl]‐4‐ylmethyl)‐7‐methoxy‐1,2,3,4‐tetrahydroisoquinolin‐6‐yl)acetyl)‐L –threoninate (**Y7r**): Yield 61%, white solid. ^1^H NMR (400 MHz, DMSO‐*d*_6_) *δ* 7.79 (d, *J* = 6.1 Hz, 1H), 7.66 (d, *J* = 7.1 Hz, 2H), 7.62 (d, *J* = 7.8 Hz, 2H), 7.49–7.40 (m, 4H), 7.35 (t, *J* = 7.3 Hz, 1H), 6.91 (s, 1H), 6.75 (s, 1H), 5.00 (s, 1H), 4.29 (dd, *J* = 8.5, 3.3 Hz, 1H), 4.19 (dd, *J* = 9.1, 4.6 Hz, 1H), 4.11 (d, *J* = 7.2 Hz, 1H), 3.67 (s, 3H), 3.63 (s, 3H), 3.46 (d, *J* = 12.0 Hz, 2H), 3.23 (dd, *J* = 13.5, 4.4 Hz, 1H), 3.14 (q, *J* = 5.9 Hz, 1H), 2.94 (dd, *J* = 13.5, 9.2 Hz, 1H), 2.85 (dd, *J* = 12.2, 5.8 Hz, 1H), 2.63 (q, *J* = 7.1, 6.4 Hz, 2H), 1.23 (s, 1H), 1.06 (d, *J* = 6.3 Hz, 3H) (Figure S49).^13^C NMR (101 MHz, DMSO) *δ* 171.23, 170.71, 154.95, 140.12, 138.70, 137.99, 137.48, 131.01, 130.19, 128.91, 127.20, 126.50, 126.47, 126.11, 122.43, 108.76, 66.32, 57.84, 56.51, 55.32, 51.78, 41.28, 40.15, 36.38, 27.88, 20.07 (Figure S50). HRMS (ESI Positive) m/z: calcd for C_30_H_35_N_2_O_5_ [M + H]^+^: 503.2468; found: 503.2545.

Synthesis process of compounds **Y7s–Y7t**: Compounds **Y7h** or **Y7q** were dissolved in methanol, and the pH was adjusted to 12 with 1 M aqueous NaOH solution. The mixture was stirred at room temperature for 2 h. Reaction completion was confirmed by thin‐layer chromatography (TLC). The mixture was then concentrated under reduced pressure, and the pH was adjusted to 4‐5 with 1 M dilute hydrochloric acid, resulting in the precipitation of a white solid. The solid was isolated to afford the product **Y7s** or **Y7t**.

(2R,4R)‐1‐(2‐(1‐([1,1′‐biphenyl]‐4‐ylmethyl)‐7‐methoxy‐1,2,3,4‐tetrahydroisoquinolin‐6‐yl)acetyl)‐4‐hydroxypyrrolidine‐2‐carboxylic acid (**Y7s**): Yield 96%, white solid. ^1^H NMR (400 MHz, DMSO‐*d*_6_) *δ* 7.67 (d, *J* = 7.7 Hz, 4H), 7.55–7.43 (m, 4H), 7.37 (d, *J* = 7.4 Hz, 1H), 7.04 (d, *J* = 4.3 Hz, 1H), 6.50 (s, 1H), 4.70 (s, 1H), 4.33–4.13 (m, 2H), 3.76 (dd, *J* = 10.5, 5.7 Hz, 1H), 3.64–3.54 (m, 1H), 3.52 (s, 3H), 3.39 (q, *J* = 7.2 Hz, 3H), 3.26 (d, *J* = 7.0 Hz, 2H), 3.22–3.13 (m, 2H), 3.07–2.96 (m, 1H), 2.92–2.81 (m, 1H), 2.29 (ddt, *J* = 13.8, 10.0, 5.0 Hz, 1H), 1.84–1.73 (m, 1H), 1.22 (s, 1H) (Figure S51). ^13^C NMR (101 MHz, DMSO) *δ* 173.32, 168.66, 155.13, 139.88, 138.80, 136.05, 130.54, 128.99, 127.41, 126.81, 126.56, 123.48, 108.79, 68.85, 57.42, 55.26, 54.99, 54.60, 38.89, 38.69, 37.16, 34.65, 24.30 (Figure S52). HRMS (ESI Positive) m/z: calcd for C_30_H_33_N_2_O_5_ [M + H]^+^: 501.2311; found: 501.2386.

(2‐(1‐([1,1′‐biphenyl]‐4‐ylmethyl)‐7‐methoxy‐1,2,3,4‐tetrahydroisoquinolin‐6‐yl)acetyl)‐L‐alanine (**Y7t**): Yield 96%, white solid. ^1^H NMR (400 MHz, DMSO‐*d*_6_) *δ* 8.06 (d, *J* = 7.2 Hz, 1H), 7.65 (dd, *J* = 12.7, 7.8 Hz, 4H), 7.49–7.41 (m, 4H), 7.35 (t, *J* = 7.2 Hz, 1H), 6.94 (s, 1H), 6.70 (d, *J* = 3.8 Hz, 1H), 4.31 (t, *J* = 6.3 Hz, 1H), 4.21–4.11 (m, 1H), 3.64 (s, 3H), 3.36 (s, 2H), 3.29–3.24 (m, 2H), 3.03–2.89 (m, 2H), 2.70 (dd, *J* = 14.8, 8.6 Hz, 2H), 1.25 (t, *J* = 6.0 Hz, 4H) (Figure S53). ^13^C NMR (101 MHz, DMSO) *δ* 174.47, 169.51, 155.16, 139.92, 138.60, 136.67, 133.32, 130.85, 128.95, 127.34, 126.72, 126.52, 124.18, 123.77, 108.83, 55.58, 55.23, 47.95, 40.15, 39.31, 36.05, 25.34, 17.65 (Figure S54). HRMS (ESI Positive) m/z: calcd for C_28_H_31_N_2_O_4_ [M + H]^+^: 459.2206; found: 459.2279.

### 4.2 Pharmacophore mapping

Select Ligand Pharmacophore Mapping under the Pharmacophores module in the Discovery Studio 2019 software with the following screening parameters (Table 5).

**TABLE 5:** Parameters used for Ligand Pharmacophore Mapping.

Parameter name	Parameter value
Conformation generation	CAESAR
Discard existing conformations	True
Energy threshold	20.0
Best mapping only	True
Options	Fit most features
Maximum omitted features	−1
Fitting method	Flexible
Minimum interfeature distance	0.5
Map each conformation separately	True
Fit name	FitValue
Scale fit values	True

### 4.3 Molecular docking

The crystal structures of PD‐L1/BMS‐1166 complex (PDB ID:6R3K) and hPD‐1/hPD‐L1 (4ZQK) were obtained from the Protein Data Bank (https://www.rcsb.org/). Discovery Studio 2019 software was selected and the crystal structures were pre‐treated with Prepare Protein before docking to remove all water molecules, CHARMm pre‐treated small molecule junctions to minimize their energy, retaining the D‐chain in 6R3K and the small molecule with the A‐chain in 4ZQK, and then superimposed the D‐chain in 6R3K with the A‐chain in 4ZQK to de‐emphasize the D‐chain to redefine the binding site. Finally, the docking was completed using CDOCKER under the Dock Ligands module. The structure of the compound‐protein complex was visualized and analyzed using PYMOL.

### 4.4 In vitro PD‐1/PD‐L1 HTRF binding assay

The blocking effect of the compounds on the interaction between PD‐1 and PD‐L1 was assessed using the PD‐1/PD‐L1 binding assay kit (Cisbio, 64PD1PEG). The solid form of the compound to be tested was dissolved in DMSO to form a 10 mM stock solution, which was then diluted with purified water to 10 times the intended test concentration, ensuring that the DMSO content remained below 0.5%. In each well of a white 96‐well plate, 2 μL of the diluted compound was added, and 2 μL of purified water was added to the control wells. Subsequently, 4 μL of Tag1‐labeled PD‐L1 protein and 4 μL of Tag2‐labeled PD‐1 protein were introduced and incubated for 15 min at room temperature. Following that, 10 μL of a premixed solution containing anti‐Tag1‐Eu^3+^ and anti‐Tag2‐XL665 was added, and the plate was sealed for a 3‐h incubation at room temperature. Finally, the fluorescence signals excited at 330nm were measured using a Biotek Cytation 5 Multimode reader, with readings taken at 665 and 620 nm. The inhibition rate was calculated as the ratio of the HTRF signals, following the provided protocol.

(1) $$\begin{array}{c} \text{Emission}\;\text{Ratio}\left(\text{ER}\right)=\frac{{\text{Emisssion}\;\text{signal}}_{665\text{nm}}}{{\text{Emisssion}\;\text{signal}}_{620\text{nm}}}\times 10^{4}. \end{array}$$

(2) $$\begin{array}{c} \text{Inhibition}\;\text{rate}=\frac{{\text{ER}}_{\text{positive}}\;-\;{\text{ER}}_{\text{sample}}}{{\text{ER}}_{\text{positive}}\;-\;{\text{ER}}_{\text{negative}}}\times 100\%. \end{array}$$

### 4.5 SPR analysis

The binding affinities of compounds to hPD‐L1 protein were assessed by a SPR assay. Briefly, The PD‐L1 protein(Sino Biological Inc:10084‐H08H) was diluted to 200 μg/mL with sodium acetate buffer (pH 4.5), then immobilized on a CM5 sensor chip (Series S Sensor Chip, GE Healthcare) by the standard amine coupling reagent kit (GE Healthcare, PA), and the immobilization level reached approximately 10000 RU. The test compounds were serially diluted to appropriate concentrations with phosphate buffered saline buffer (0.1% Tween 20 + 1%DMSO) and injected at a flow through the chip following the preset program and the *K*_D_ value was calculated using Biacore T200 Evaluation software.

### 4.6 Cell culture

HepG2 and Jurkat cells were cultured in 1640 complete medium with 10% FBS and 1% penicillin‐streptomycin. The cells were incubated at 37°C in a 5% CO_2_ incubator.

### 4.7 Cytotoxicity assays

The cytotoxicity of representative compounds against HepG2 and Jurkat cell lines was tested using Cell Counting Kit‐8 (Glpbio), and the maximum non‐toxic concentration of compounds was selected. Densities of HepG2 cells (5000 cells/well) and Jurkat cells (10000 cells/well) were inoculated into 96‐well plates, respectively, and after stimulated incubation with specific concentrations of compounds for 48 h, 20 μL CCK8 was added and incubation was continued for 2 h, and the wavelength of 490 nm was measured with a microplate reader to detect the cell viability.

### 4.8 Cell co‐culture experiments

The activation of Jurkat cells to kill HepG2 cells by representative compounds was tested using Cell Counting Kit‐8 (Glpbio). The maximum non‐toxic concentration of the compounds was selected, and a density of 10 ng/mL INF‐γ (Invivogen)‐stimulated HepG2 cells (1000 cells/well) and 2 μg/mL PHA‐P (Invivogen)‐stimulated Jurkat cells (10,000 cells/well) were inoculated into a 96‐well plate with the specific concentration of the compound stimulation after 48 h of incubation, 20 ul CCK8 was added and incubation was continued for 2 h, and the wavelength of 490 nm was measured with a microplate reader to detect cell viability.

(3) $$\text{Cell}\;\text{viability}\left(\%\right)=\frac{A_{\text{drug}\;}\;-\;A_{\text{blank}}}{A_{\text{control}}\;-\;A_{\text{blank}}}\times 100\%.$$

### 4.9 Cell recovery activity assay in Jurkat cells^[26]^

Jurkat cells were seeded in a 96‐well plate in 50 μL, of RPMl‐1640 containing 10% FBS with a density of 10,000 cells/well for 6 h at 37°C. Next, Jurkat cells were stimulated by 25 μL/mL antiCD3/CD28 (ImmunoCult, catalog #10981) without or with 25 μL human PD‐L1 protein (MCE. catalog #HY‐P73361) with a density of 1.25 μg/well, followed by addition of blank culture medium, BMS‐1(10 μM), **Y7f**(1.1 μM), **Y7g**(0.37 μM), **Y7q**(0.37 μM) or **Y7r(**1.1 μM), 25 μL, respectively. Then, above mixtures were cultured at 37°C for 24 h. After that time, the supernatants were diluted 1:1 and harvested for detection of lFN‐γ levels according to the manufacturer's protocol by using a human IFN‐γ ELISA Kit (Multi Sciences, catalog #EK180‐90). The results were carried out by the GraphPad Prism 9 software.

### 4.10 Calculation of AlogP

Using Discover Studio 2019 software, small molecules were run by selecting the AlogP command of Molecular Properties.

## AUTHOR CONTRIBUTIONS

Yueqing Li was in charge of the entire research, designed experiments, and supervised manuscript revisions. Menglin Yu designed and synthesized derivatives based on compound **Y6**, carried out CADD research as well as HTRF and SPR evaluations, conducted cell experiments and wrote the first draft. Sen Cai synthesized some derivatives based on compound **Y6**. Yanyan Pan, Heshuang Wang and Li Zhang carried out flow cytometry studies. Fengwu Zhang was mainly responsible for the synthesis of compound **Y6** and the optimization of cyclization conditions. Aiyu Ma conducted the molecular dynamic simulation. Shixuan Lv did T‐Cell recovery activity assay in Jurkat Cells. Xiuhan Guo and Shisheng Wang participated in the experimental design and result analysis. Qiling Song contributed to the analysis of in vitro experimental results. Chunyan Ma took part in the analysis of flow cytometry data. Qingwei Meng supervised the experiment and provided experimental funds. Jian Wang and Shuai Wang were in charge of the experimental materials.

## CONFLICT OF INTEREST STATEMENT

The authors declare no conflicts of interest.

## ETHICS STATEMENT

No animal or human experiments were involved in this study.

## Supporting information

Supporting Information S1

## Data Availability

The data that support the findings of this study are available from the corresponding author upon reasonable request.
