# Supplementary material for: Design, synthesis and activity evaluation of tetrahydroisoquinoline‐based programmed cell death ligand 1 inhibitors
Source: Smart Mol. 2026 Jul 28:e70075. Online ahead of print. doi: 10.1002/smo2.70075 (PMC13410806; doi:10.1002/smo2.70075)
Supplement: Supplementary file 1 — Supporting Information S1 [file SMO2-9999-0-s001.docx]

**Supporting Information for Original article**

**Design, Synthesis and Activity Evaluation of Tetrahydroisoquinoline-based PD-L1 Inhibitors**

**Menglin Yu,^a^ Sen Cai,^a^ Yanyan Pan,^c^ Fengwu Zhang,^a^ Aiyu Ma,^a^ Shixuan Lv,^a^ Xiuhan Guo,^a,b^ Shisheng Wang,^a,b^ Shuai Wang,^a,b^ Qingwei Meng,^a,b^ Jian Wang,^d^ Yueqing Li^a,b,*^**

^a^ *State Key Laboratory of Fine Chemicals, Department of Pharmaceutical Sciences, School of Chemical Engineering, Dalian University of Technology, 2 Linggong Road, Dalian 116012 (China)*

^b^ *Ningbo Institute of Dalian University of Technology，No.26 Yucai Road, Jiangbei District, Ningbo, China, 315016*

^c^ *Department of Central Laboratory, Dalian Municipal Central Hospital, Dalian, Liaoning, China，116023*

*^d^ Viwit Pharmaceuticals Limited, 88 Weizhi Road, Tengzhou Biopharma Park, Shandong，China，277500*

*Corresponding author(s). Tel.: +86 13504248501;

E-mail address(es): yueqingli@dlut.edu.cn

Supplementary information

[1. Molecular stacking analysis 2](#_Toc8987)

[2. The impact of compounds on Jurkat cells secretion of IFN-γ 4](#_Toc13568)

[3. Molecular dynamics simulation 5](#_Toc20453)

[4.](#_Toc3181) ^[1](#_Toc3181)^[H NMR,](#_Toc3181) ^[1](#_Toc3181)^[C NMR spectra of compounds 9](#_Toc3181)

# Molecular stacking analysis


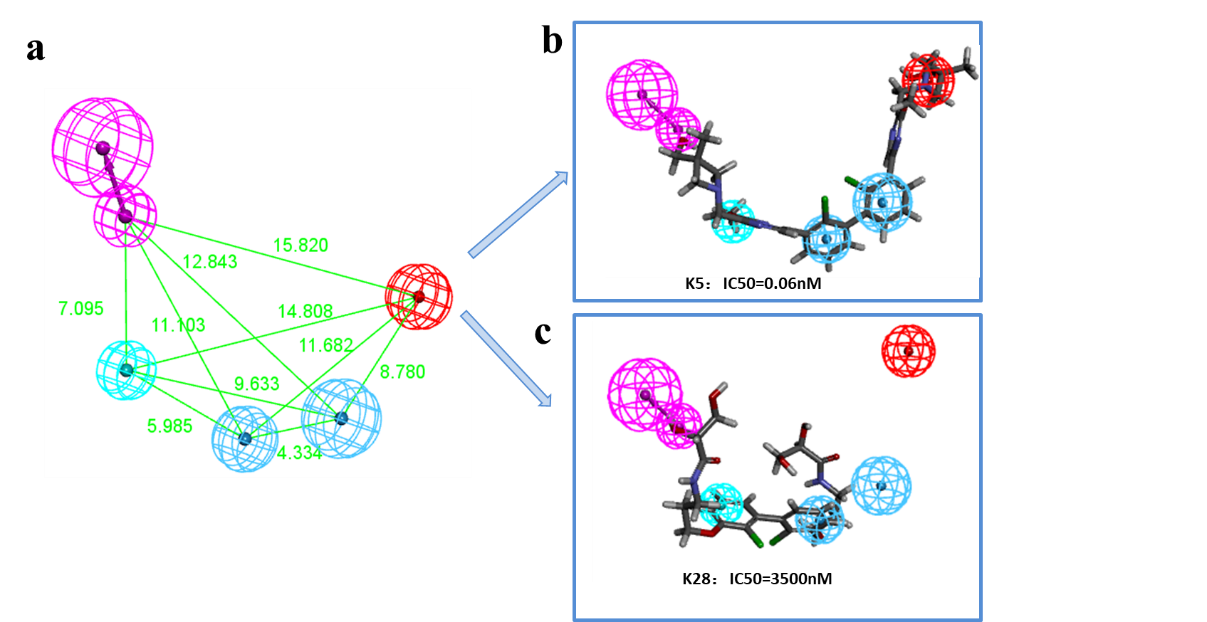


**Figure S1 Pharmacophore features and the distance between them in the pharmacophore model: HBD (magenta), HY (cyan), PC (red), and HYA (blue).**

**Figure S2 hydrophilic nitrogen-containing side chains used in the molecular design**


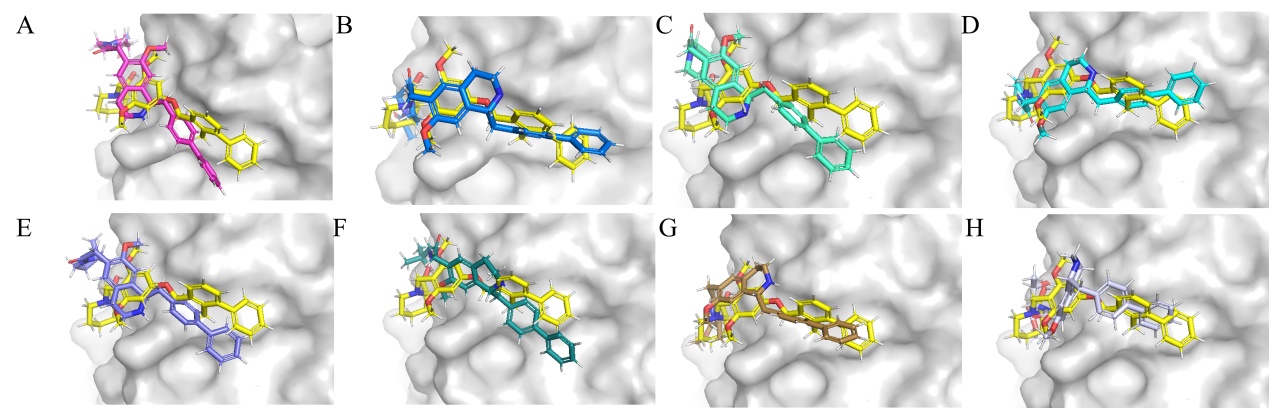


**Figure S3. Overlapping structure of compound and BMS-1(yellow)structure at the binding interface of PD-L1. (PDB:4ZQK).**

A)Compound Y7b; B)Compound Y7d; C)Compound Y7e; D)Compound Y7f; E)Compound Y7g; F)Compound Y7m; G) Compound Y7q; H)Compound Y7r

Table S1 -CDocker interaction energy scores

| **Compd.** | **-CDocker ineraction energy (Kcal/mol)** | **Compd.** | **-CDocker ineraction energy (Kcal/mol)** | **Compd.** | **-Cdocker ineraction energy(Kcal/mol)** |
| --- | --- | --- | --- | --- | --- |
| **Y6** | **23.3101** | **Y7g** | **25.9788** | **Y7o** | **30.1369** |
| **Y7** | **26.4151** | **Y7h** | **27.488** | **Y7p** | **33.0164** |
| **Y7a** | **28.5086** | **Y7i** | **29.2019** | **Y7q** | **30.9203** |
| **Y7b** | **25.5204** | **Y7j** | **31.8712** | **Y7r** | **33.6931** |
| **Y7c** | **29.3103** | **Y7k** | **30.7367** | **Y7s** | **29.0714** |
| **Y7d** | **29.952** | **Y7l** | **29.423** | **Y7t** | **29.1282** |
| **Y7e** | **27.3956** | **Y7m** | **30.3858** |  |  |
| **Y7f** | **31.4962** | **Y7n** | **29.2088** |  |  |

# The impact of compounds on Jurkat cells secretion of IFN-γ

**
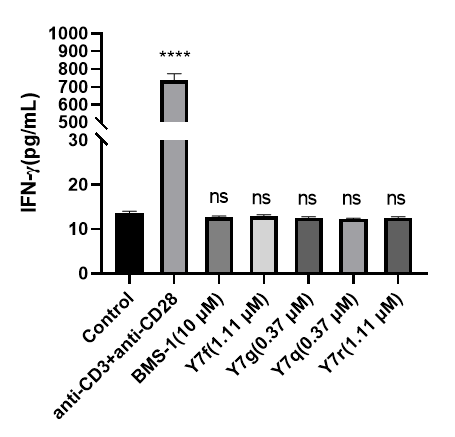
**

**Figure S4 IFN-γ secretion by Jurkat cells in response to compounds treatment**

# Molecular dynamics simulation

Molecular dynamics simulations were performed using GROMACS 2023.2 for the PD-L1 protein (PDB ID: 6R3K) complexed with **(1R,16R)-Y7f**, **(1R,16S)-Y7f**, **(1S, 16R)-Y7f**, **(1S,16S)-Y7f** and the reference compound **BMS-1**. The detailed setup was as following:

**Force Field Parameters**

Protein: AMBER14SB force field

Ligands: Parameters generated with the General Amber Force Field (GAFF)

**System Setup**

Explicit solvation: TIP3P water molecules

Neutralization: Na^+^/Cl^-^ ions added to achieve charge neutrality

Ionic concentration: 0.15 M (physiological saline)

**Energy Minimization**

Algorithm: Steepest descent

Convergence criterion: Energy tolerance ≤ 100 kJ·mol^-^¹·nm^-^¹

Short-range van der Waals: 1.0 nm

Coulomb: 1.0 nm

Cutoff scheme: Verlet

Long-range electrostatics: Particle Mesh Ewald (PME)

**System Equilibration**

NVT ensemble: 200 ps at 300 K

Temperature coupling: V-rescale thermostat

NPT ensemble: 200 ps at 1 bar

Pressure coupling: C-rescale barostat

**Shared settings:**

Time step: 2 fs

Bond constraints: LINCS (all hydrogens)

Neighbor searching: Verlet + grid

**Production Simulation**

Duration: 200 ns

Time step: 2 fs

Trajectory output frequency: Every 10 ps

Table S2 MM/PBSA Free Energy Components for PD-L1 Complexes with **(1R,16R)-Y7f**, **(1R,16S)-Y7f**, **(1S, 16R)-Y7f**, **(1S,16S)-Y7f** and **BMS-1**

| **Energy Term** | **Value (kcal/mol)** | | | | | **Interaction Type** | **Contribution to Binding** |
| --- | --- | --- | --- | --- | --- | --- | --- |
|  | **(1R,16R)-Y7f** | **(1R,16S)-Y7f** | **(1S,16R)-Y7f** | **(1S,16S)-Y7f** | **BMS-1** |  |  |
| **E _vdW_** | -69±0.16 | -58.55±0.21 | -55.37±0.28 | -54.46±0.17 | -62.26±0.19 | Hydrophobic/packing | Favorable |
| **E_EL_** | -17.47±0.32 | -19.01±0.34 | -10.57±0.43 | -6.54±0.24 | -23.23±0.36 | Electrostatic (H-bonds/salt bridges) | Favorable |
| **E****_PB_** | 45.27±0.32 | 42.2±0.32 | 32.03±0.34 | 29.17±0.27 | 49.88±0.35 | Electrostatic desolvation penalty | Unfavorable |
| **E_nonpolar_** | -5.51±0.01 | -4.92±0.01 | -4.85±0.02 | -4.52±0.01 | -4.96±0.01 | Hydrophobic effect | Favorable |
| **ΔG_gas_** | -86.47±0.36 | -77.56±0.48 | -65.95±0.44 | -61.01±0.30 | -85.49±0.47 | Gas-phase direct interactions | Strongly |
| **ΔG_sol_** | 39.76±0.31 | 37.27±0.31 | 27.18±0.33 | 24.65±0.26 | 44.92±0.35 | Solvation penalty | Unfavorable |
| **ΔG_tot_** | -46.71±0.19 | -40.29±0.26 | -38.76±0.22 | -36.35±0.19 | -40.57±0.22 | Total binding free energy | Spontaneous |


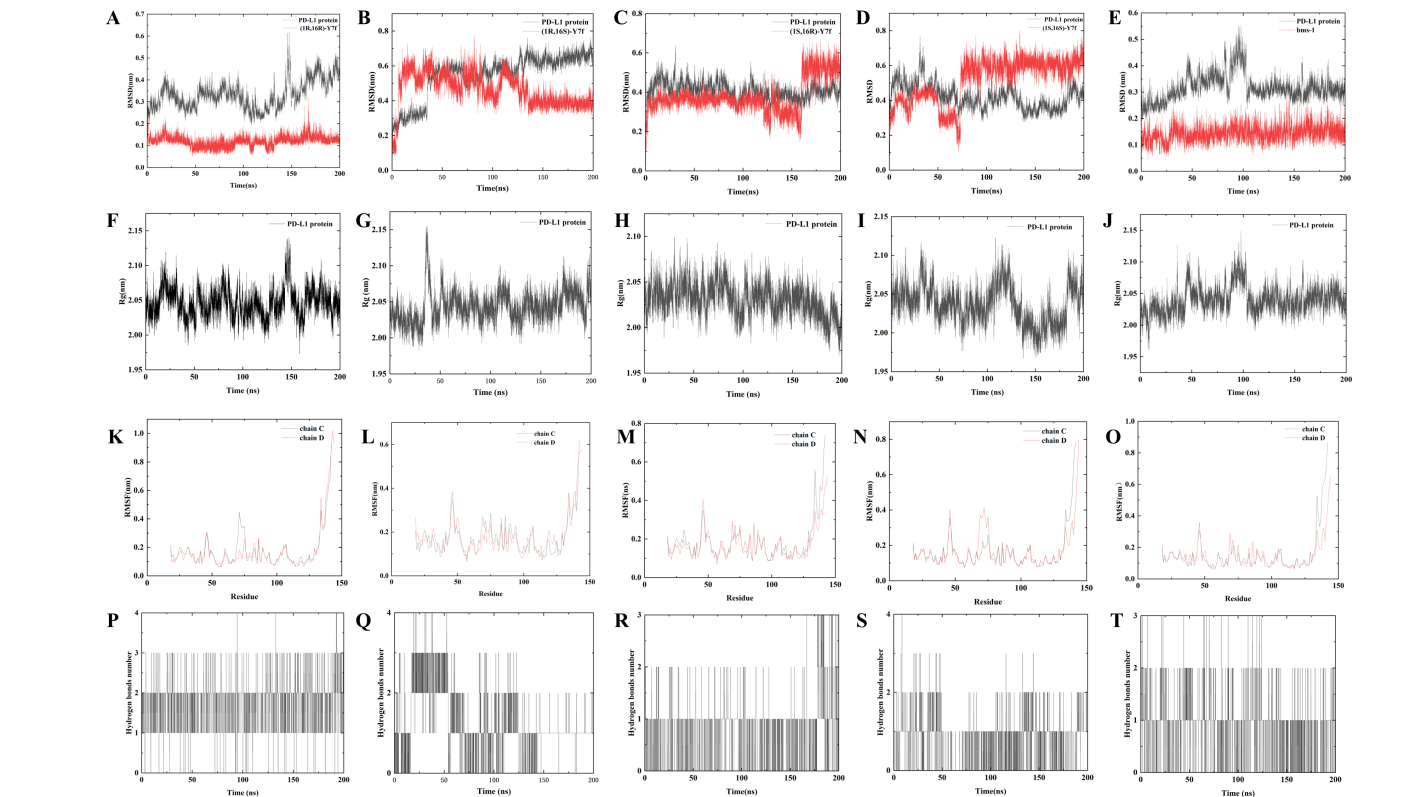


**Figure S5 Protein-Ligand interaction profiling of the 200 ns MD simulations.**

1. ~E) Root Mean Square Deviation (RMSD) of PD-L1 backbone and ligands: **(1R,16R)-Y7f** (A), **(1R,16S)-Y7f** (B), **(1S,16R)-Y7f** (C), **(1S,16S)-Y7f** (D), **BMS-1** (E); F)~J) Radius of Gyration (Rg) of PD-L1 during simulations with **(1R,16R)-Y7f** (F), **(1R,16S)-Y7f** (G), **(1S, 16R)-Y7f** (H), **(1S,16S)-Y7f** (I) or **BMS-1** (J); K)~O) Root Mean Square Fluctuations (RMSF) of PD-L1 backbone atoms (Chain C and D; PDB ID:6R3K) with **(1R,16R)-Y7f** (K), **(1R,16S)-Y7f** (L), **(1S, 16R)-Y7f** (M), **(1S,16S)-Y7f** (N) and **BMS-1** (O); P)~T) Hydrogen bonds formed between PD-L1 and the ligands during the simulations: **(1R,16R)-Y7f** (P), **(1R,16S)-Y7f** (Q), **(1S, 16R)-Y7f** (R), **(1S,16S)-Y7f** (S) and **BMS-1** (T)

​

**
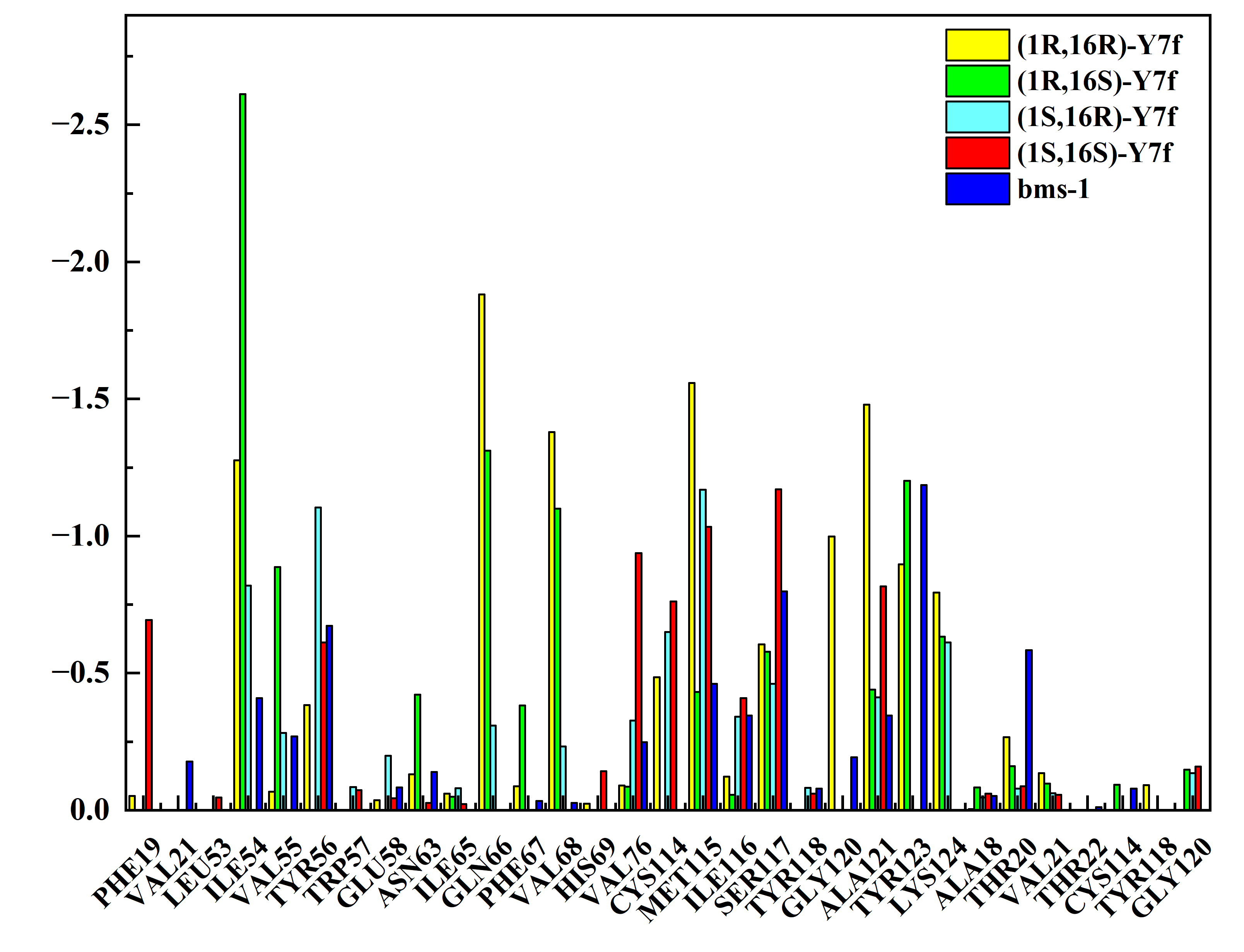
**

**Figure S6 Individual contribution of amino acids to PD-L1 and ligands binding free energy**

# ^1^H NMR, ^1^C NMR spectra of compounds


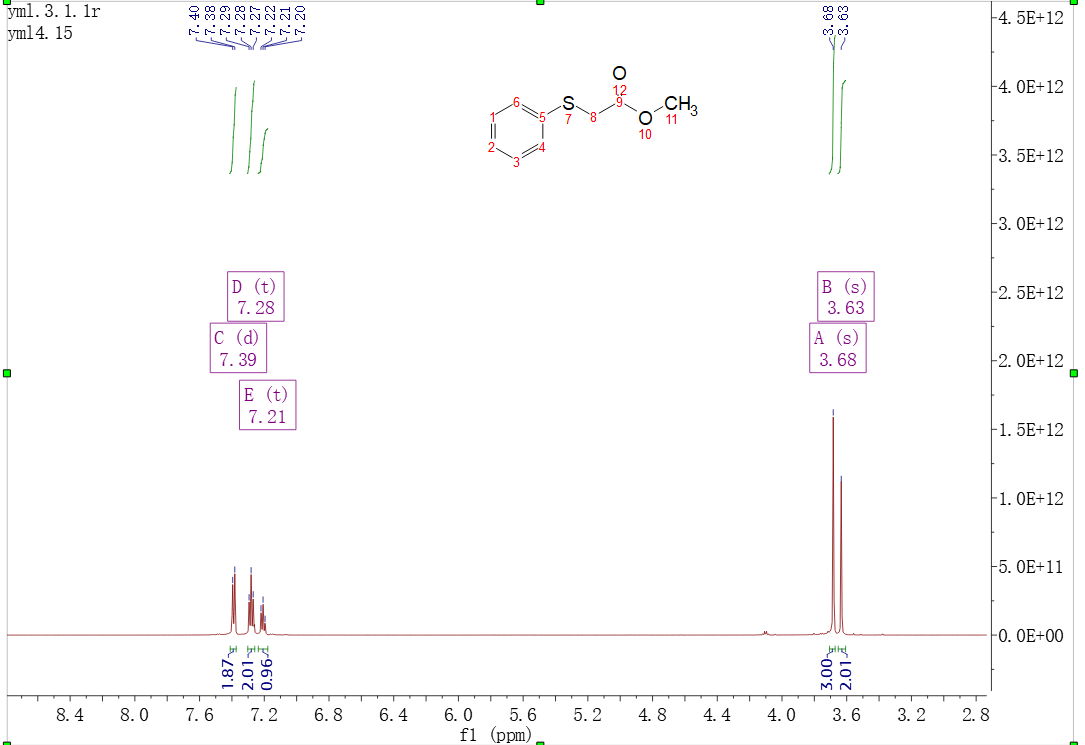


**Figure S7 ^1^H NMR spectrum of compound Y1**

**
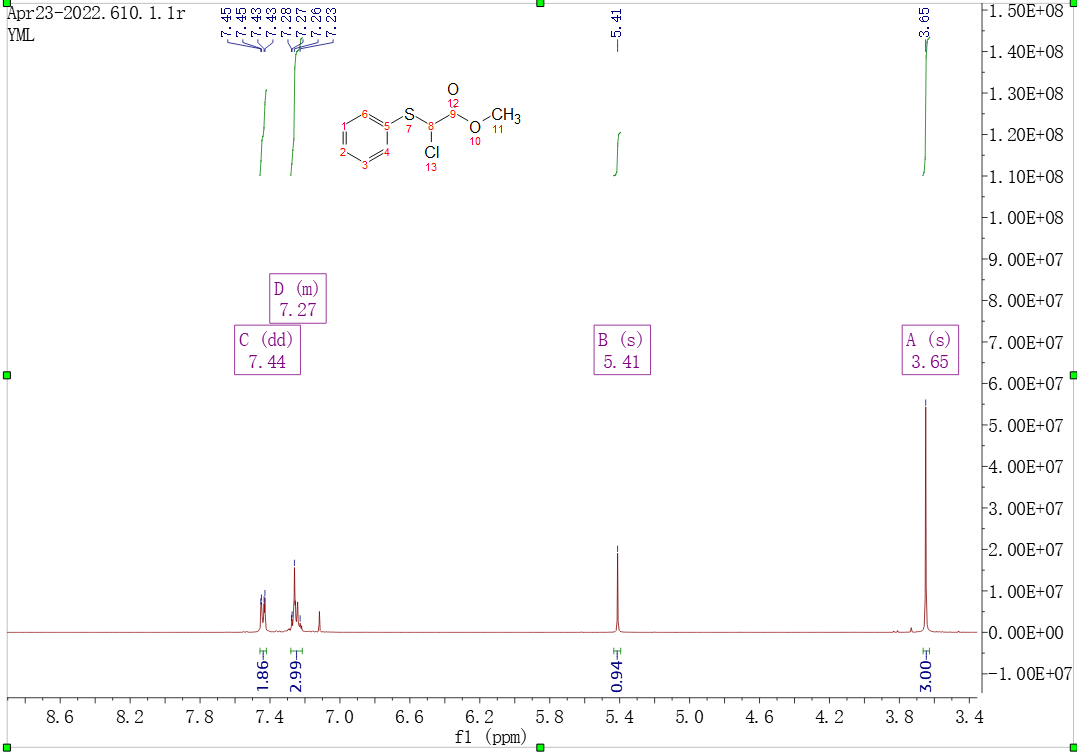
**

**Figure S8 ^1^H NMR spectrum of compound Y2**

**
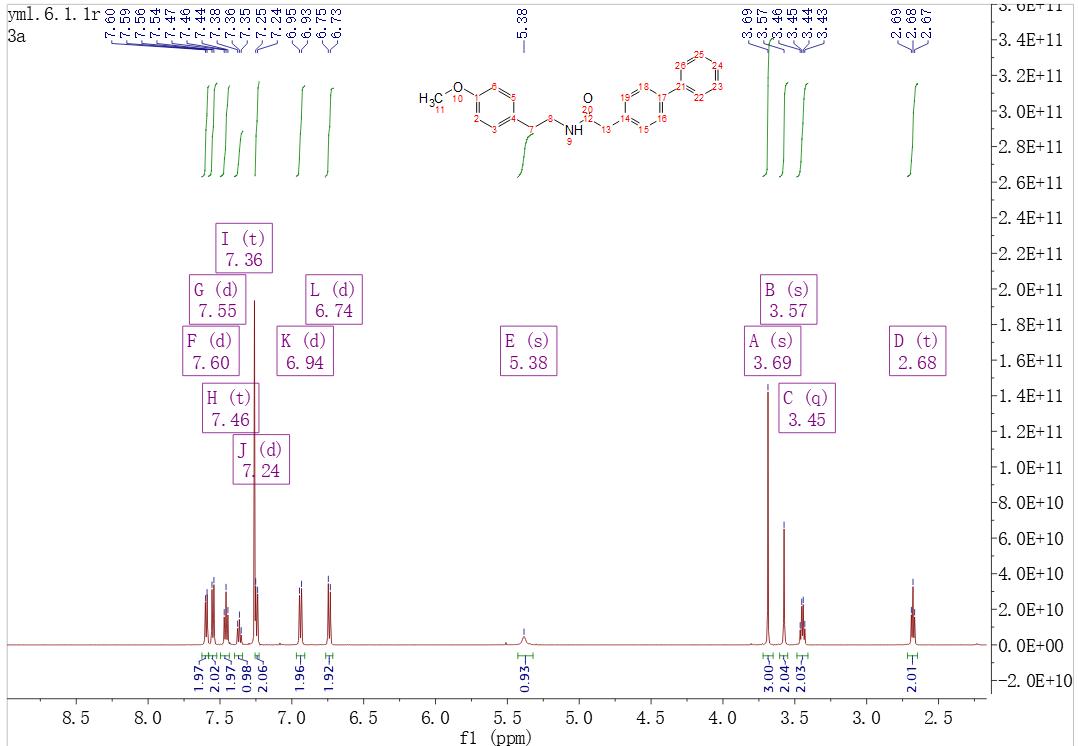
**

**Figure S9 ^1^H NMR spectrum of compound Y3**

**
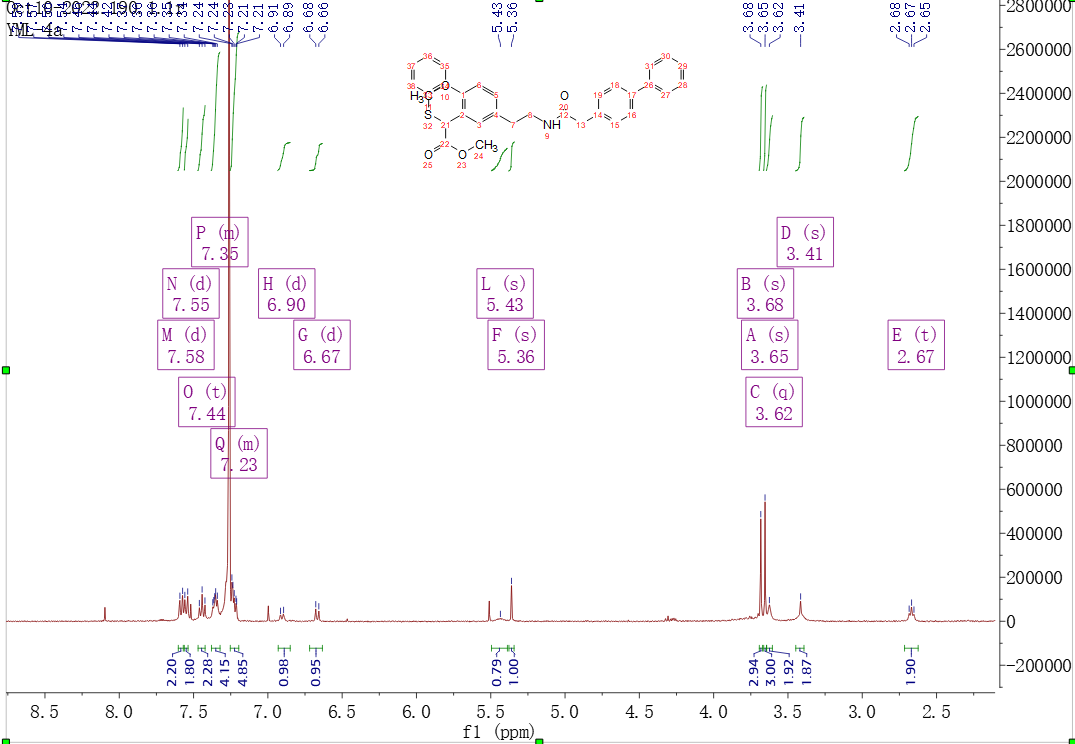
**

**Figure S10 ^1^H NMR spectrum of compound Y4**

**
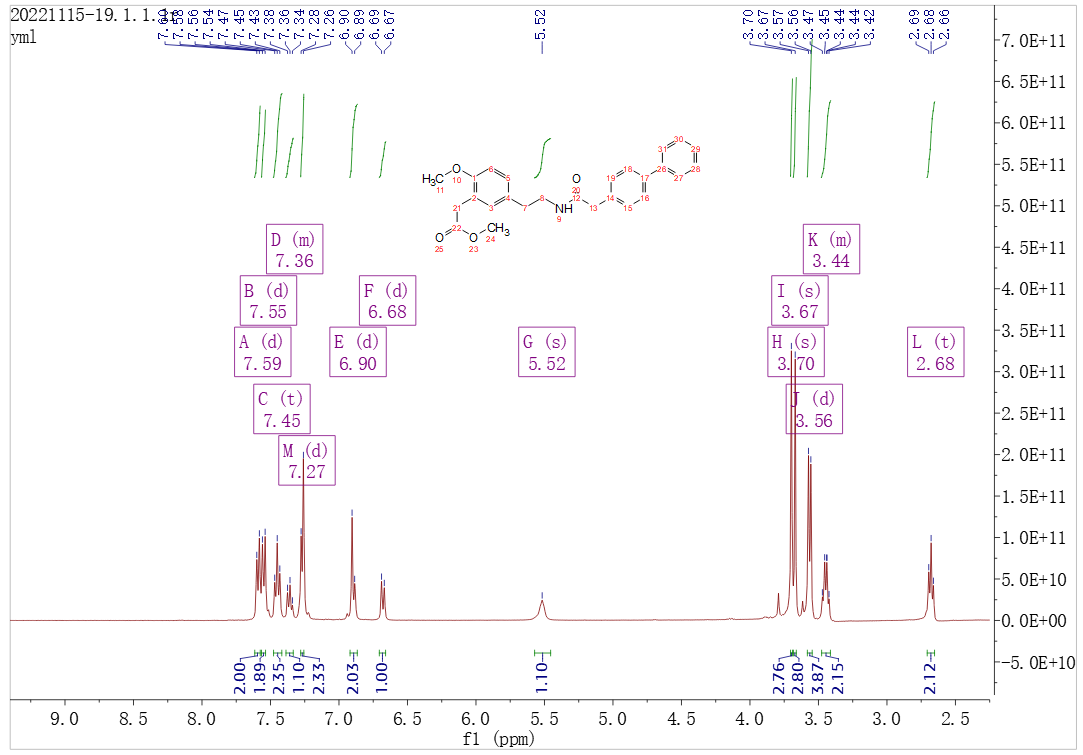
**

**Figure S11 ^1^H NMR spectrum of compound Y5**

**
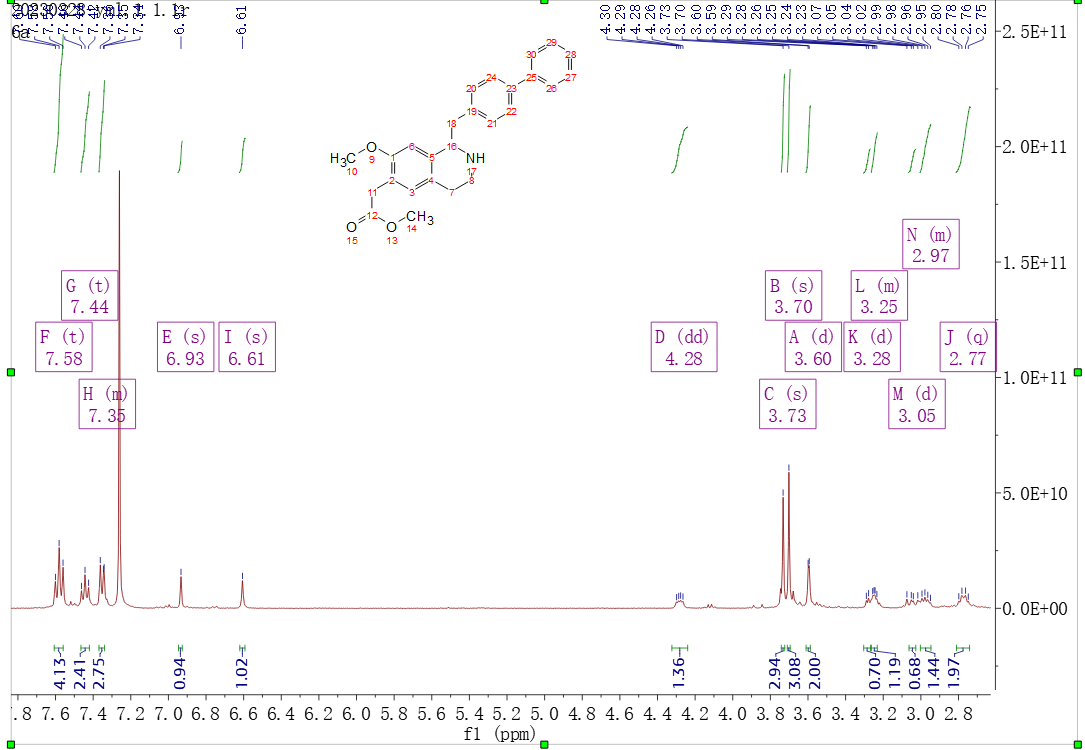
**

**Figure S12 ^1^H NMR spectrum of compound Y6**

**
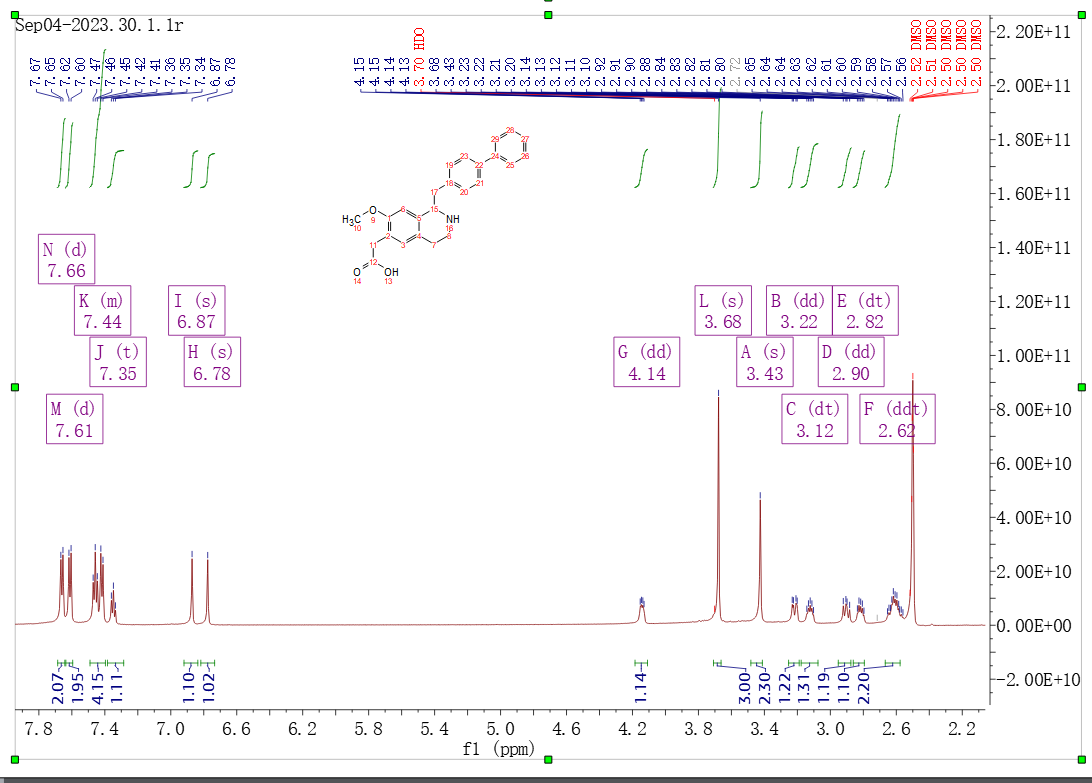
**

**Figure S13 ^1^H NMR spectrum of compound Y7**

**
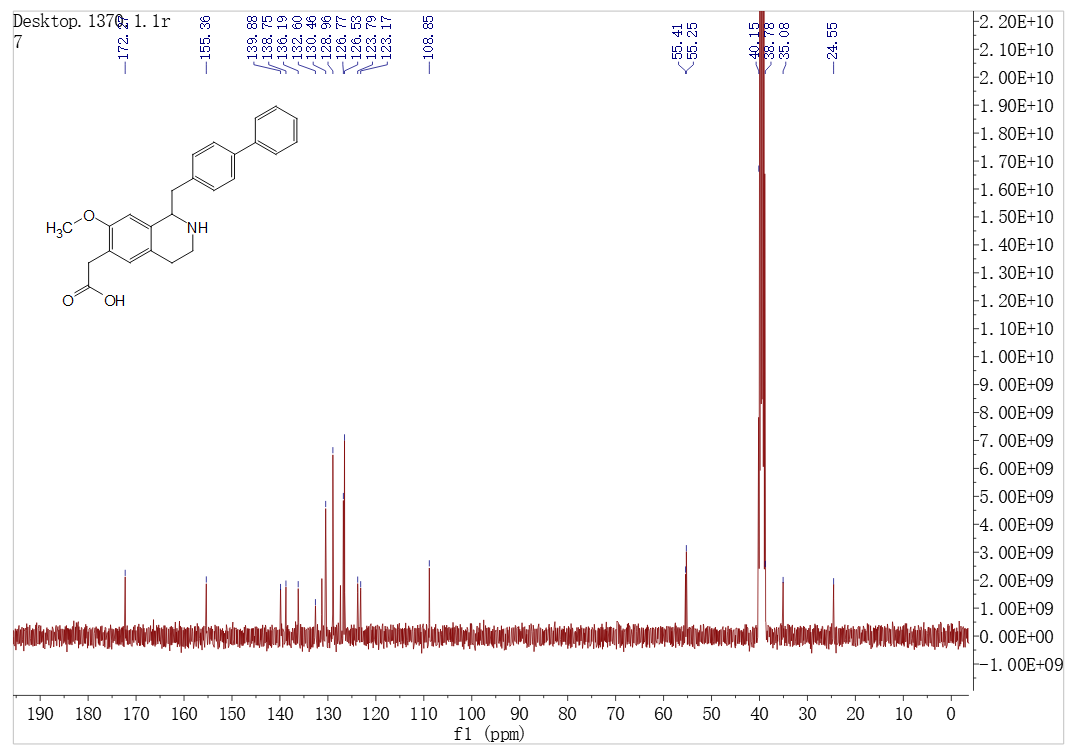
**

**Figure S14 ^13^C NMR spectrum of compound Y7**

**
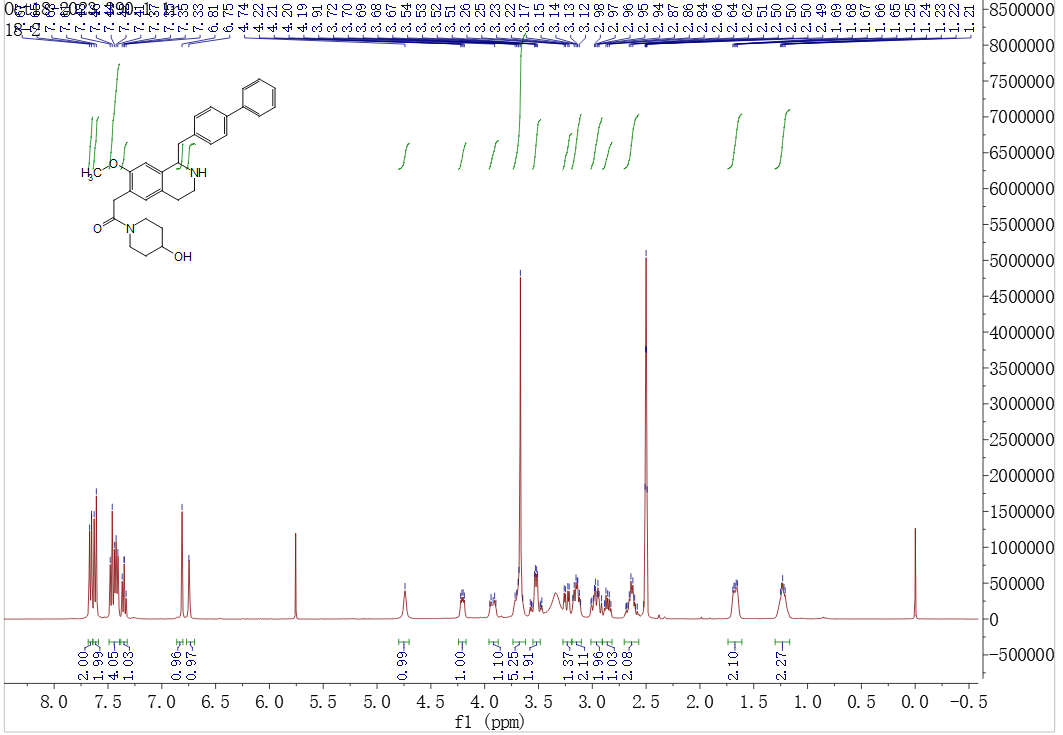
**

**Figure S15 ^1^H NMR spectrum of compound Y7a**

**
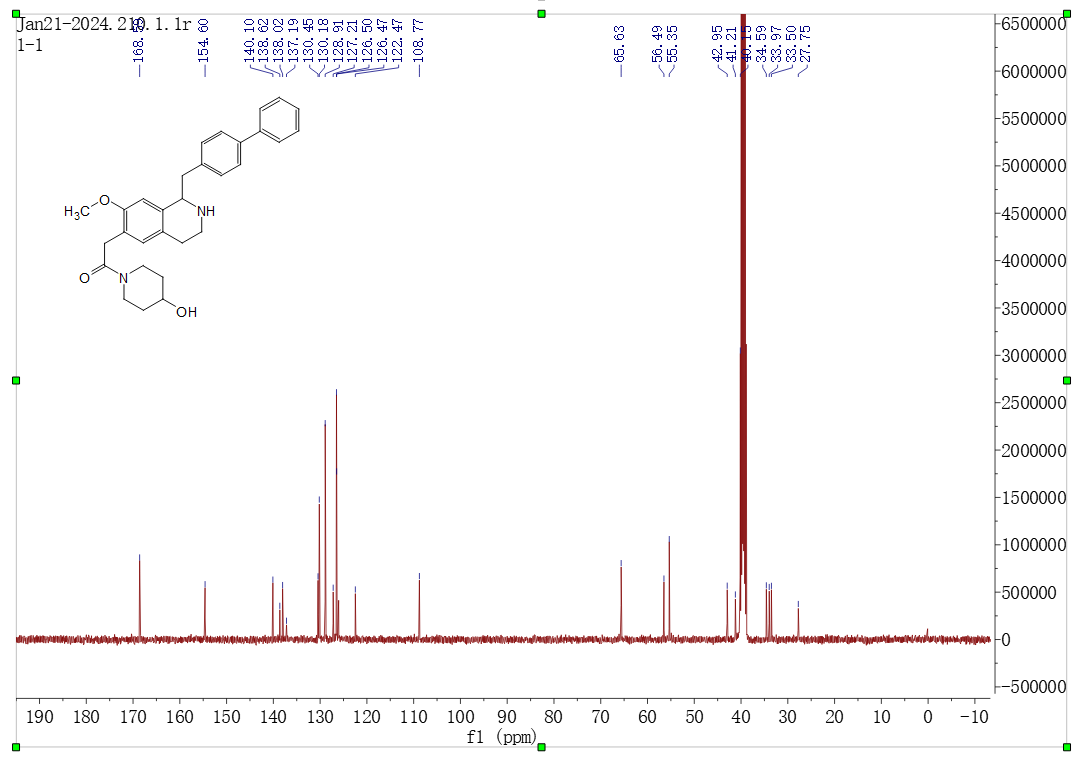
**

**Figure S16 ^13^C NMR spectrum of compound Y7a**

**
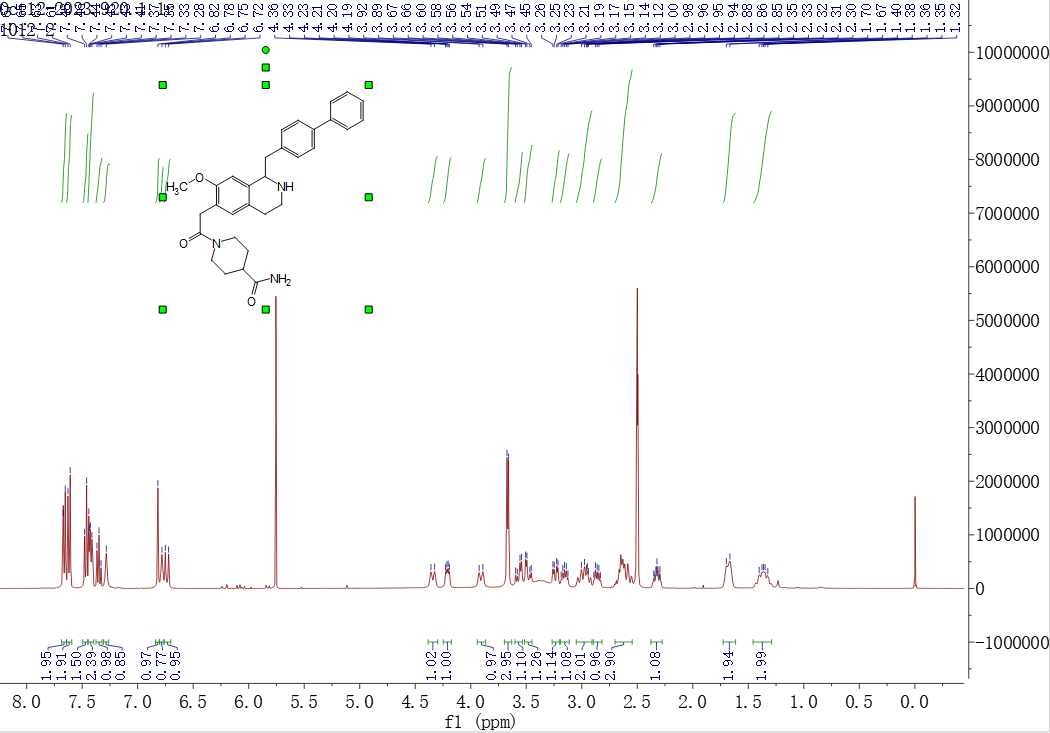
**

**Figure S17 ^1^H NMR spectrum of compound Y7b**

**
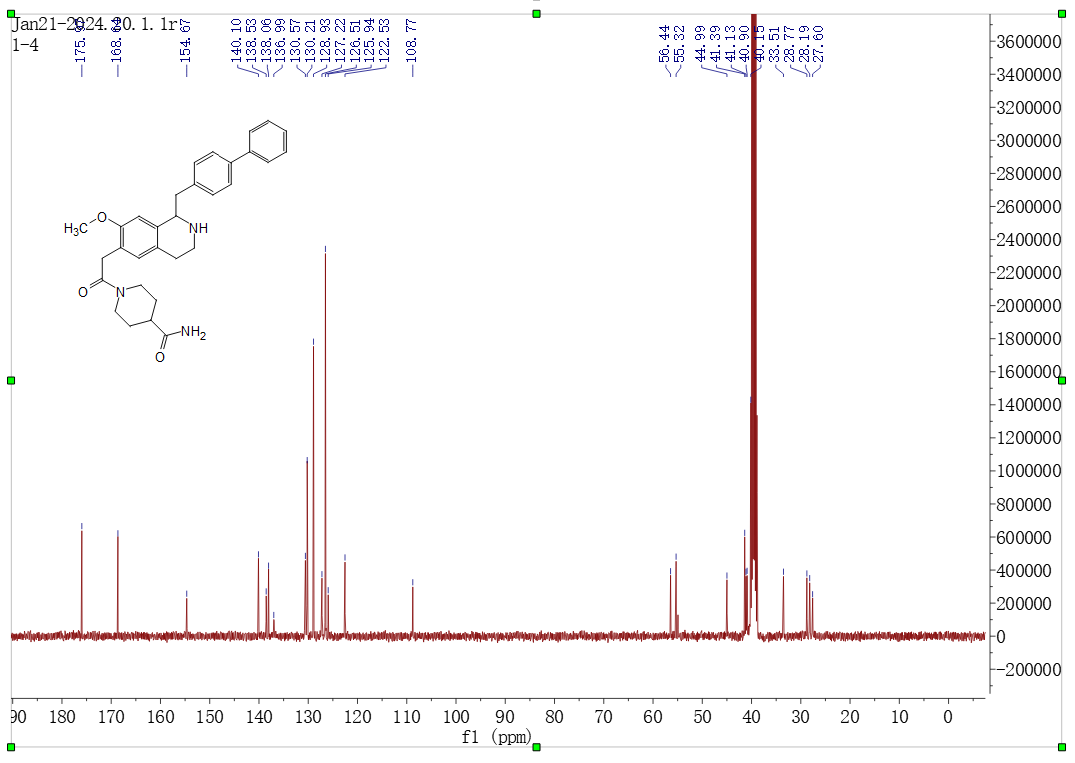
**

**Figure S18 ^13^C NMR spectrum of compound Y7b**

**
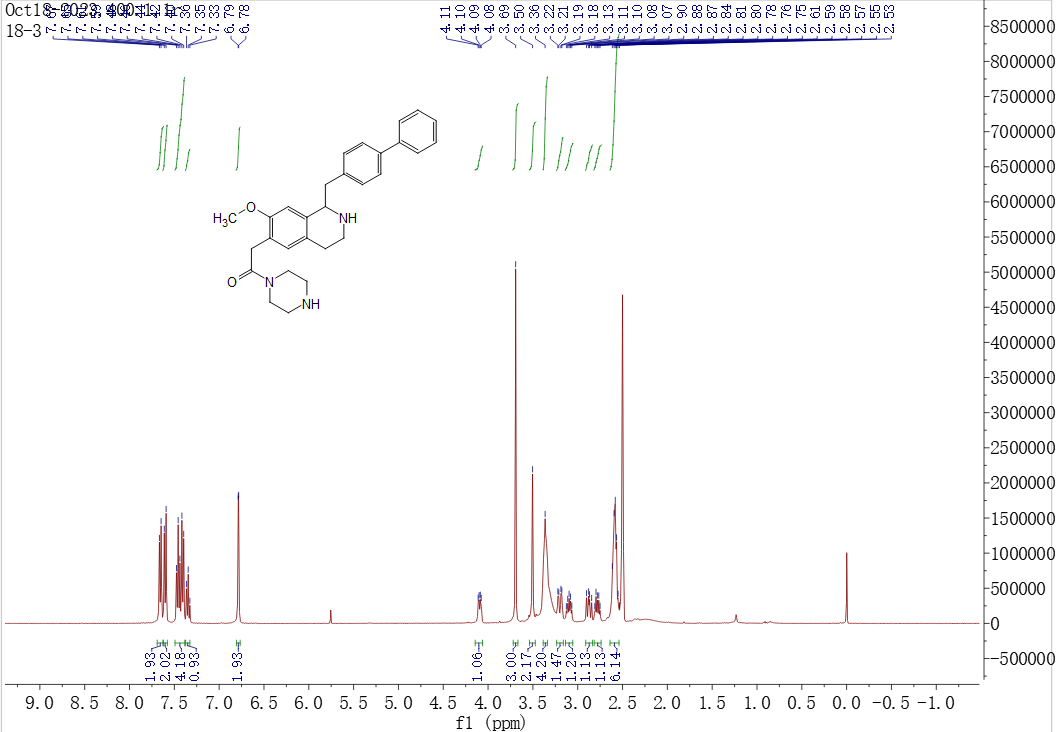
**

**Figure S19 ^1^H NMR spectrum of compound Y7c**

**
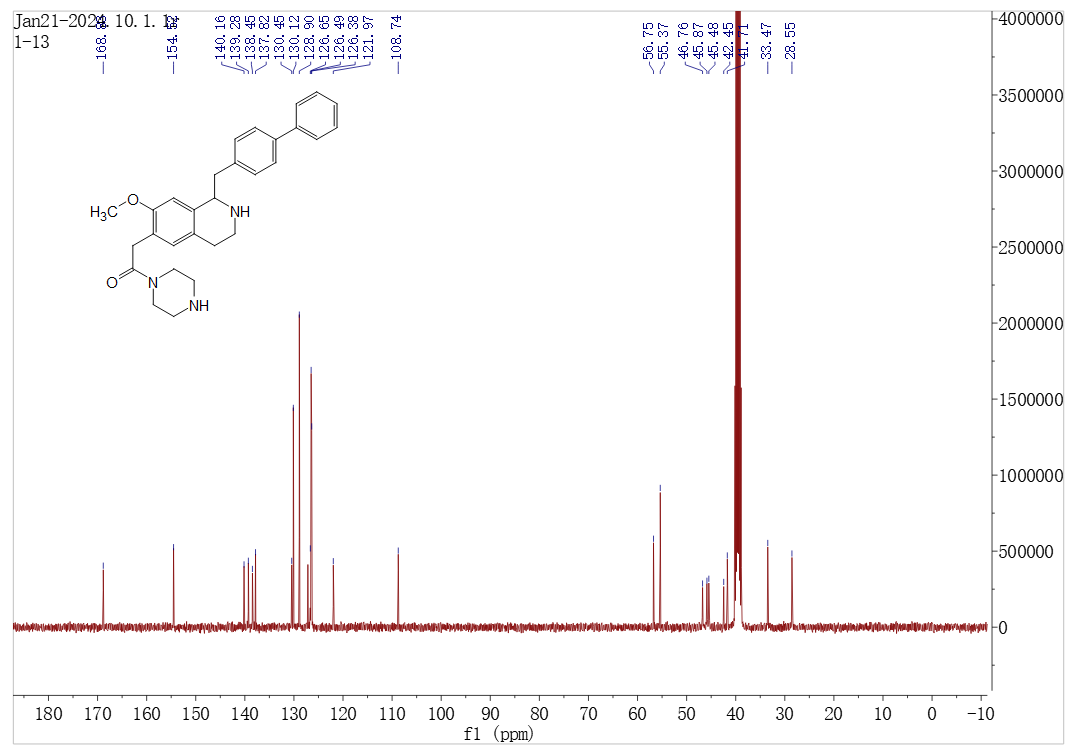
**

**Figure S20 ^13^C NMR spectrum of compound Y7c**

**
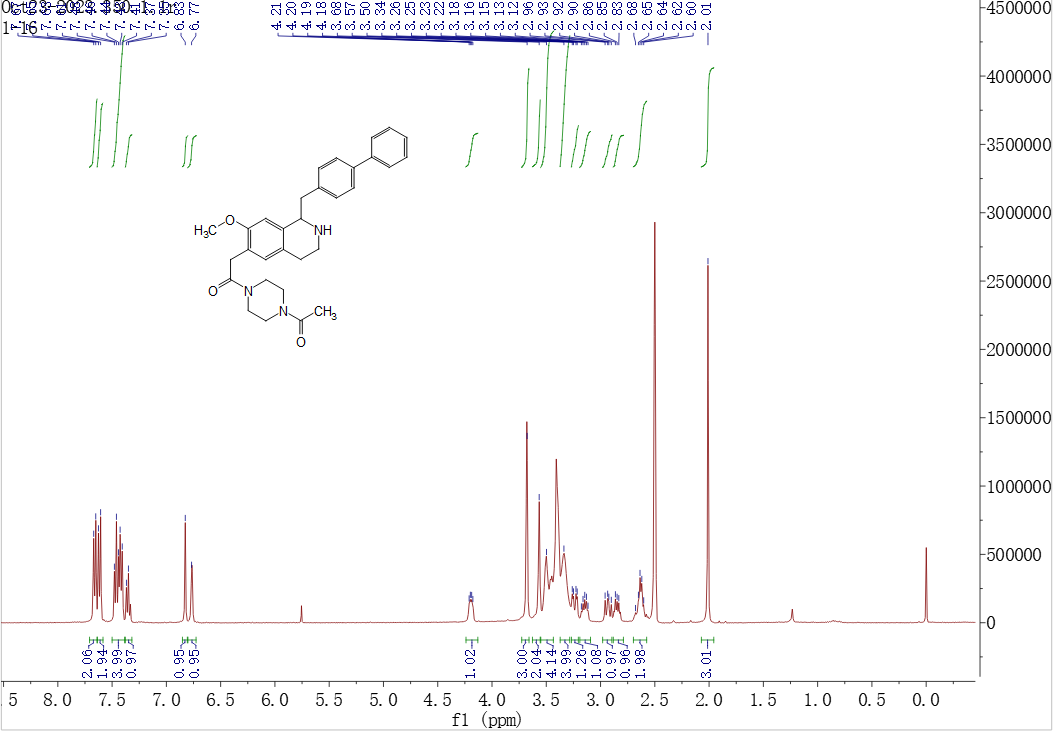
**

**Figure S21 ^1^H NMR spectrum of compound Y7d**

**
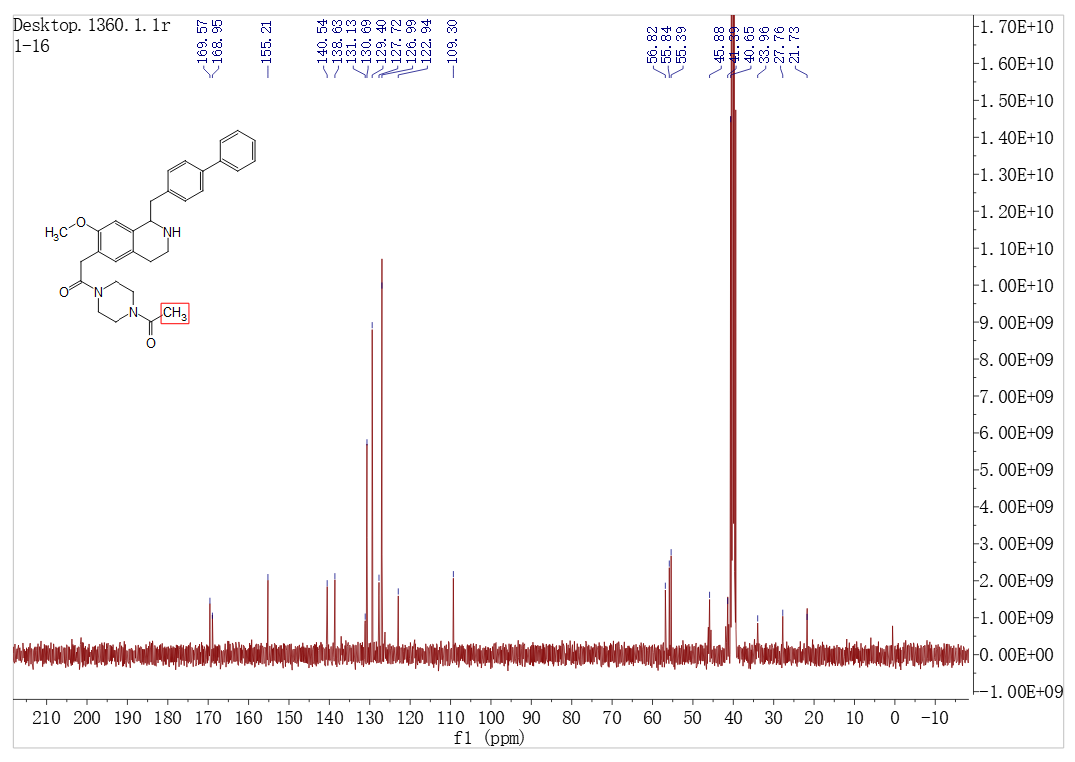
**

**Figure S22 ^13^C NMR spectrum of compound Y7d**

**
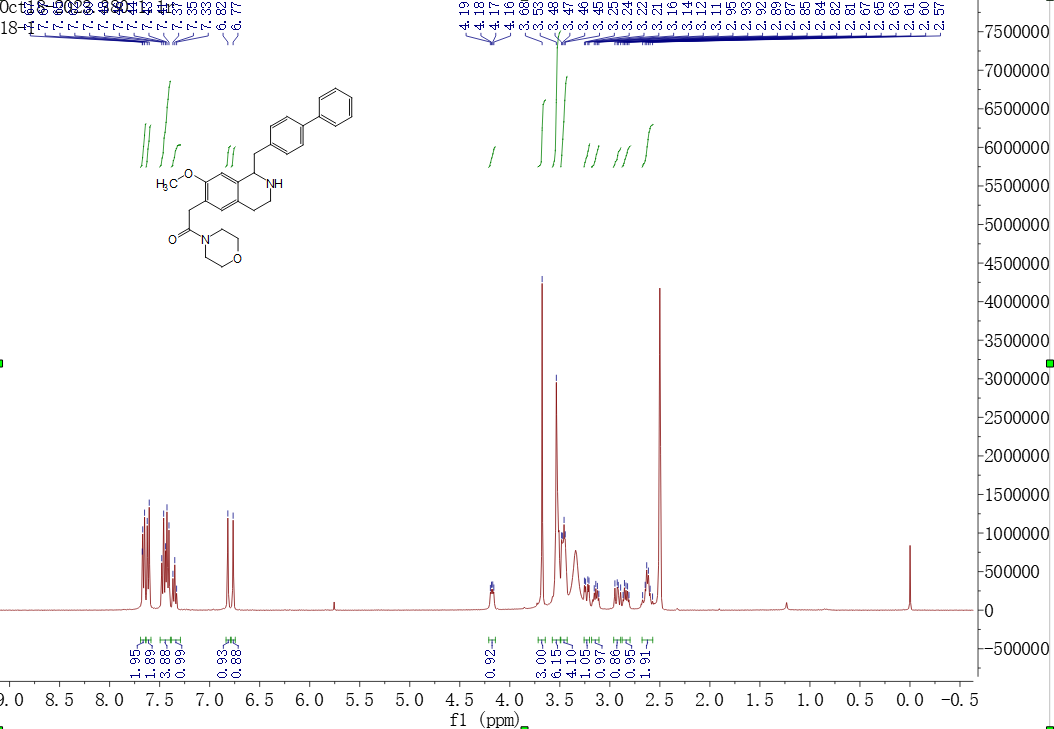
**

**Figure S23 ^1^H NMR spectrum of compound Y7e**

**
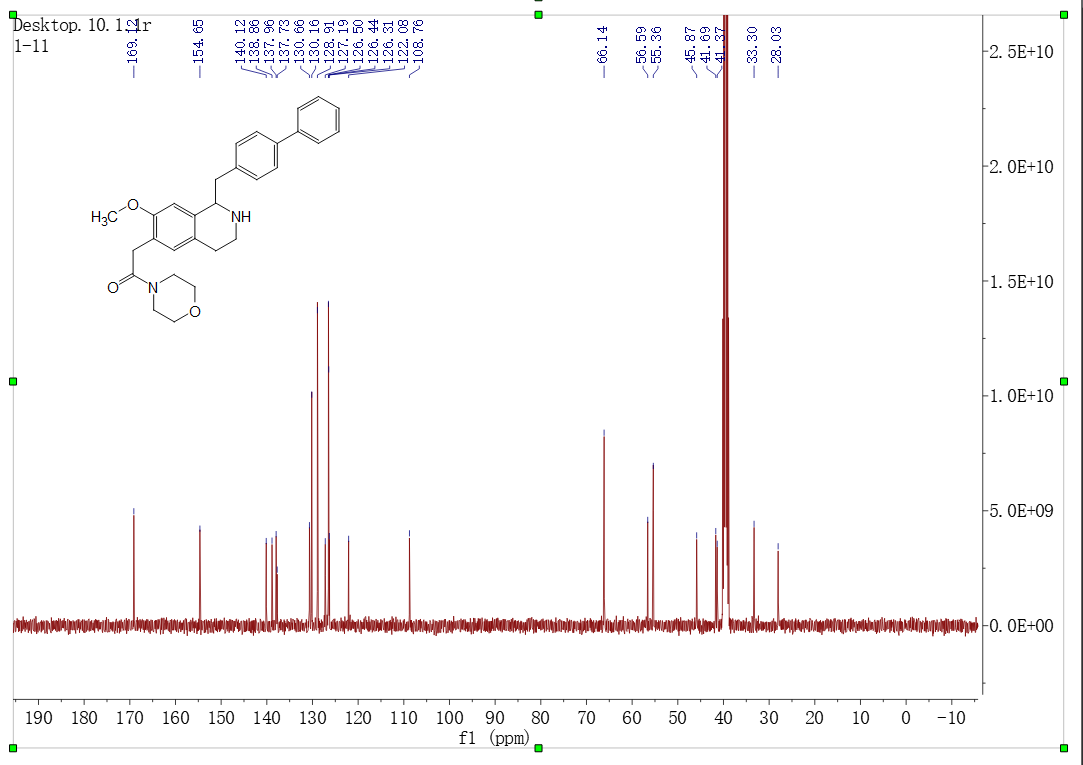
**

**Figure S24 ^13^C NMR spectrum of compound Y7e**

**
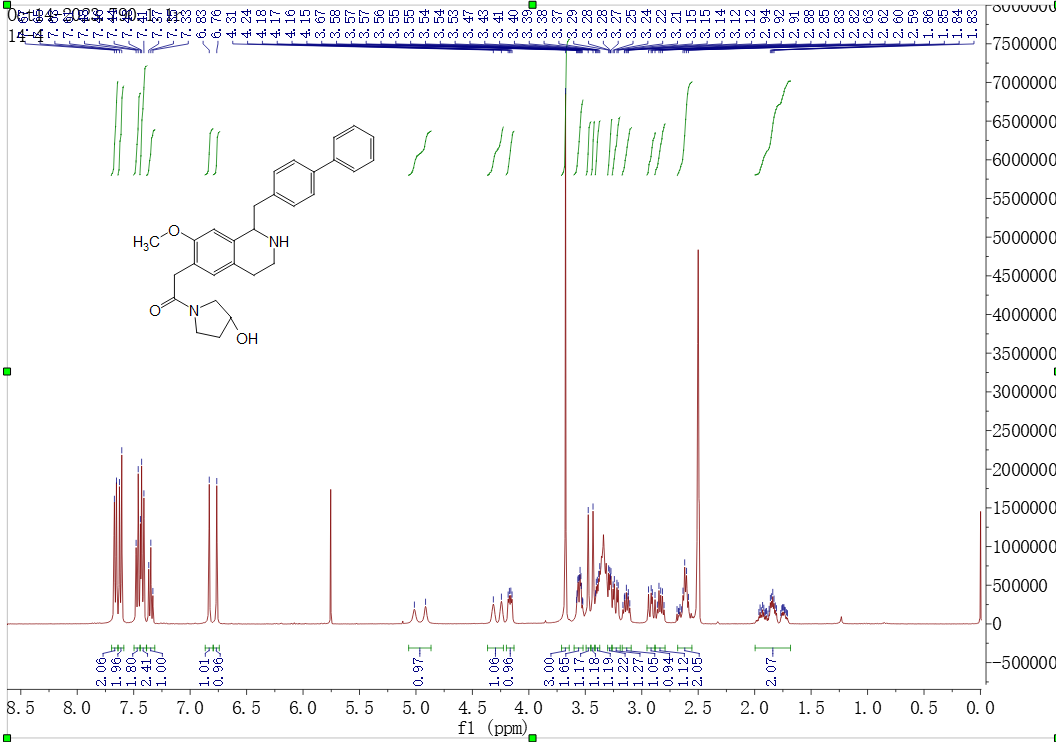
**

**Figure S25 ^1^H NMR spectrum of compound Y7f**

**
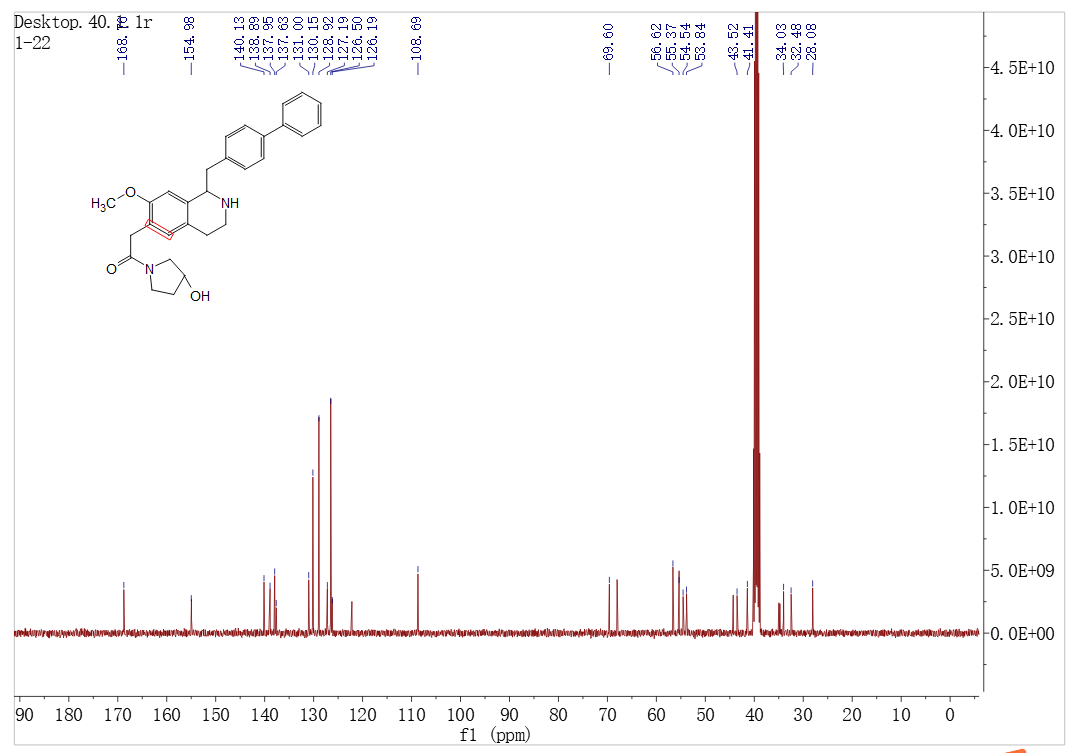
**

**Figure S26 ^13^C NMR spectrum of compound Y7f**

**
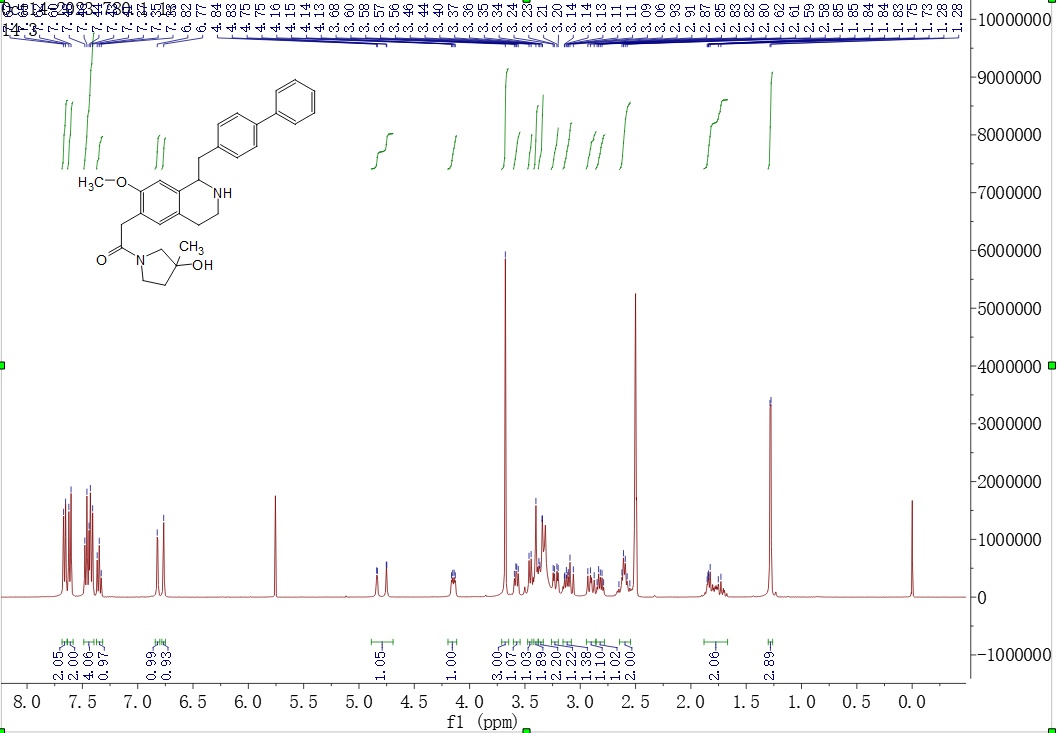
**

**Figure S27 ^1^H NMR spectrum of compound Y7g**

**
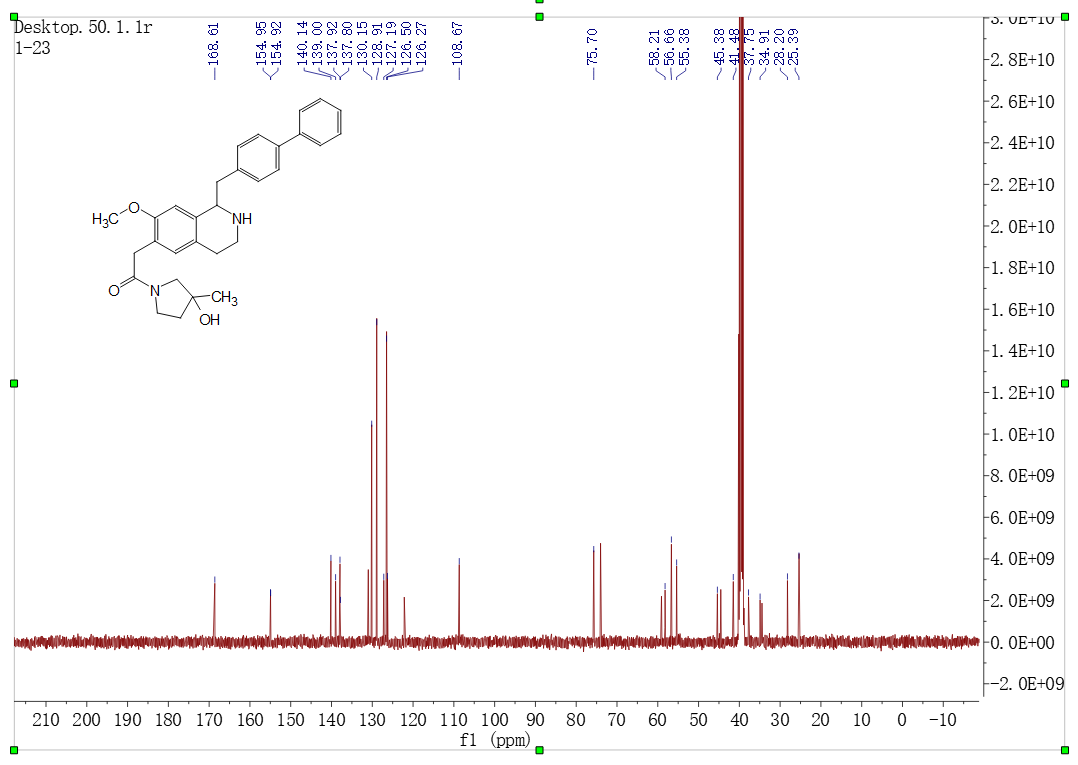
**

**Figure S28 ^13^C NMR spectrum of compound Y7g**

**
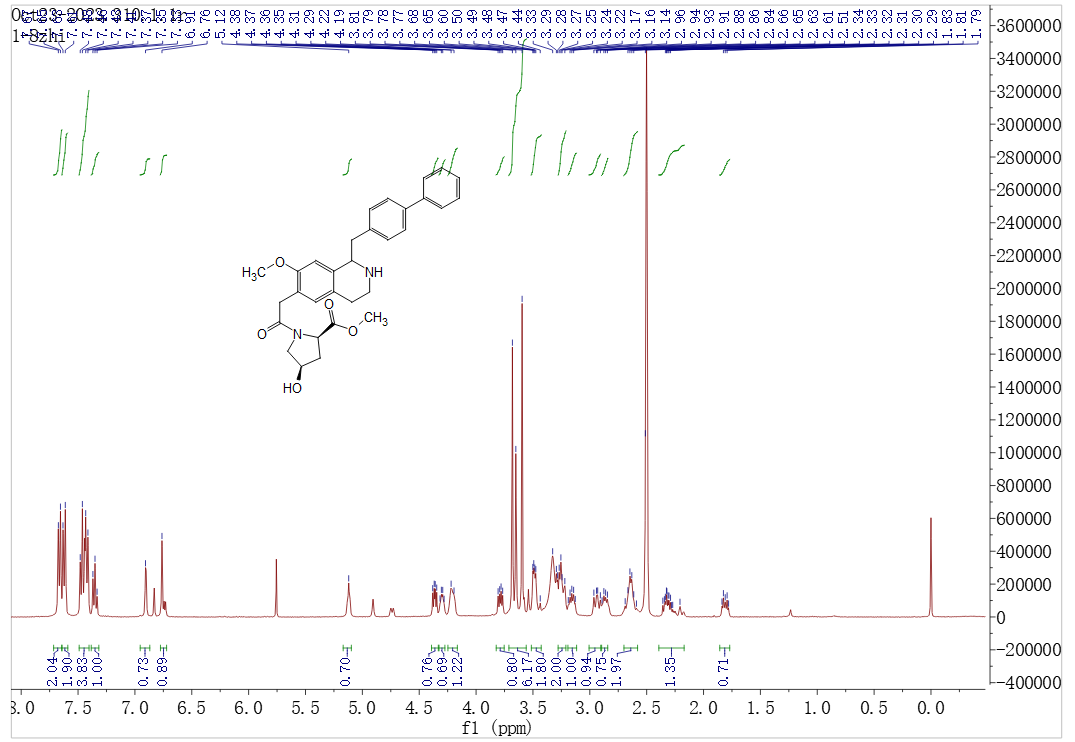
**

**Figure S29 ^1^H NMR spectrum of compound Y7h**

**
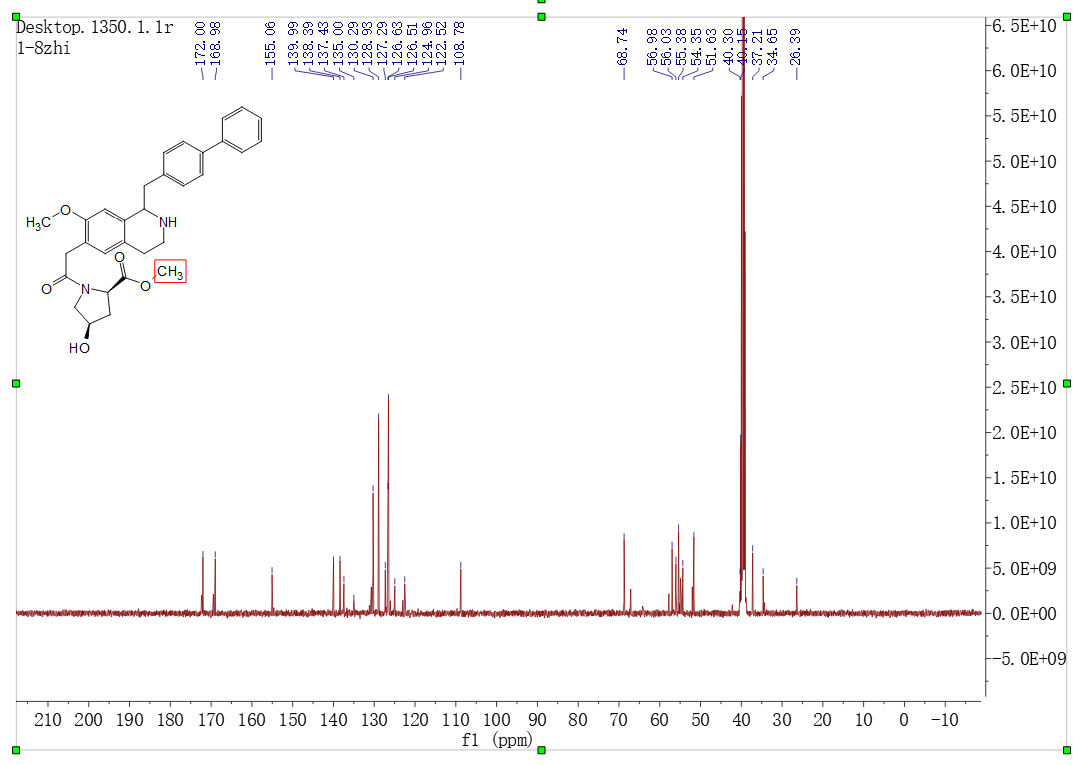
**

**Figure S30 ^13^C NMR spectrum of compound Y7h**

**
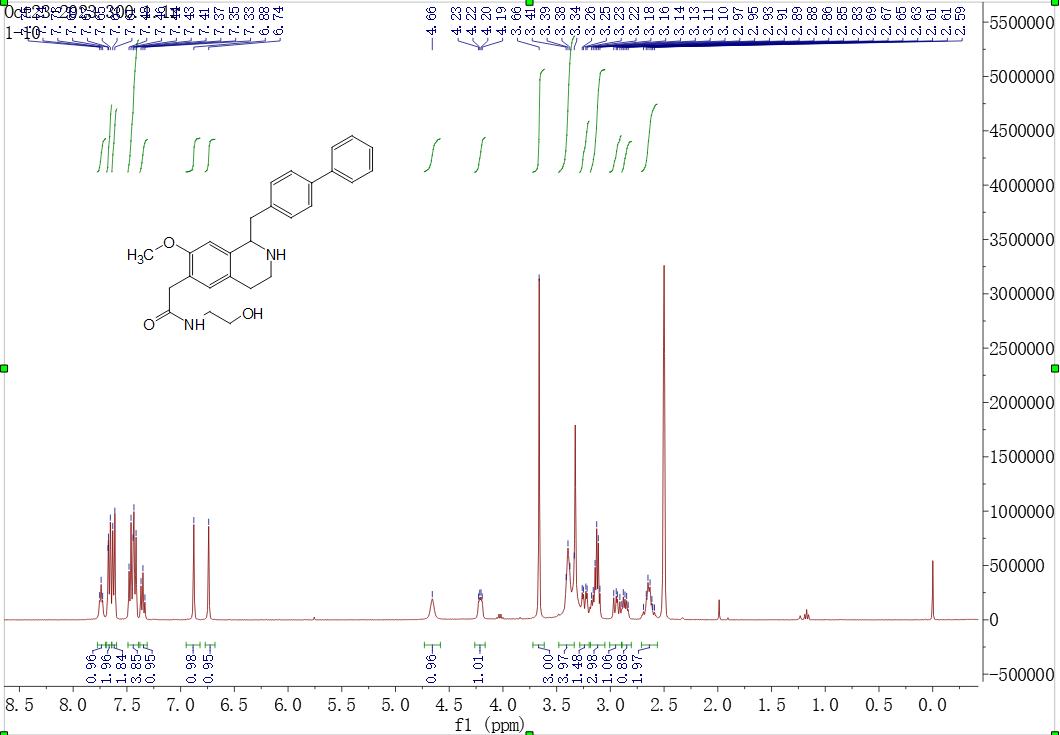
**

**Figure S317 ^1^H NMR spectrum of compound Y7i**

**
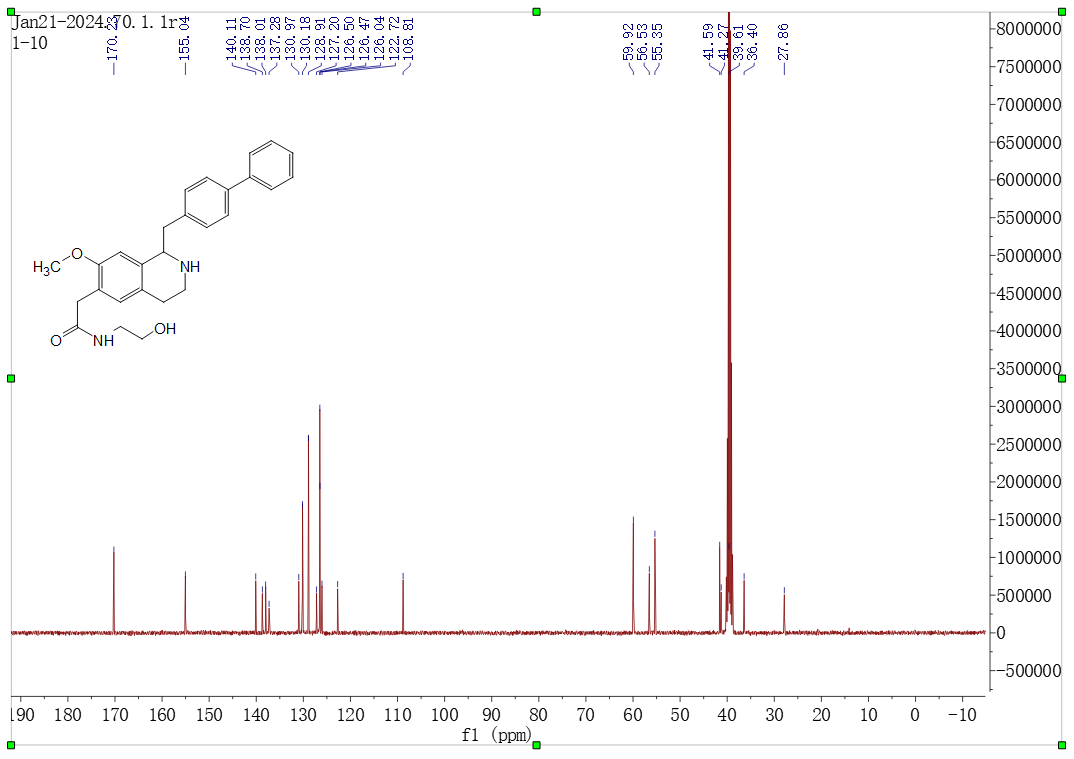
**

**Figure S32 ^13^C NMR spectrum of compound Y7i**

**
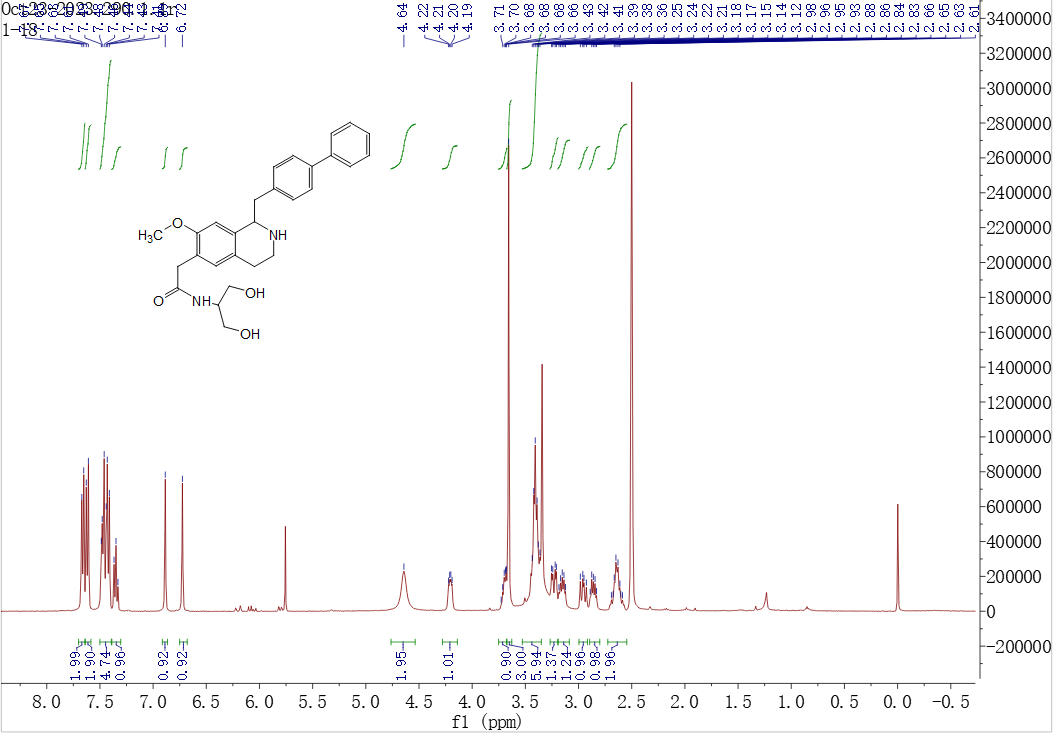
**

**Figure S33 ^1^H NMR spectrum of compound Y7j**

**
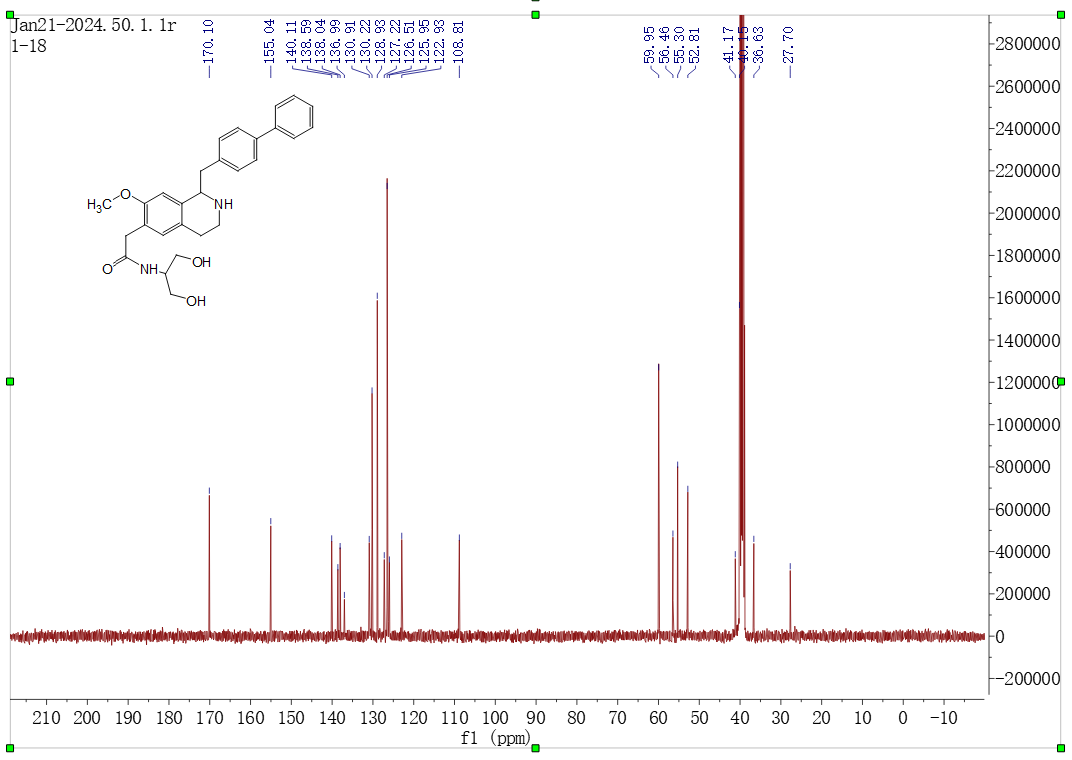
**

**Figure S34 ^13^C NMR spectrum of compound Y7j**

**
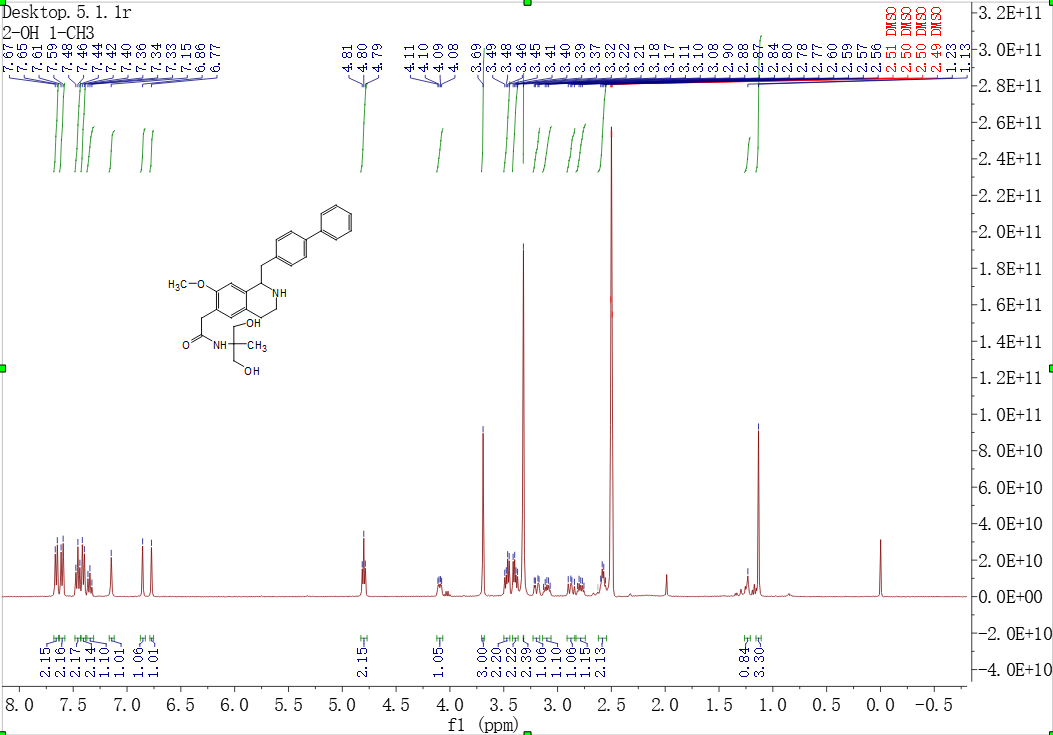
**

**Figure S35 ^1^H NMR spectrum of compound Y7k**

**
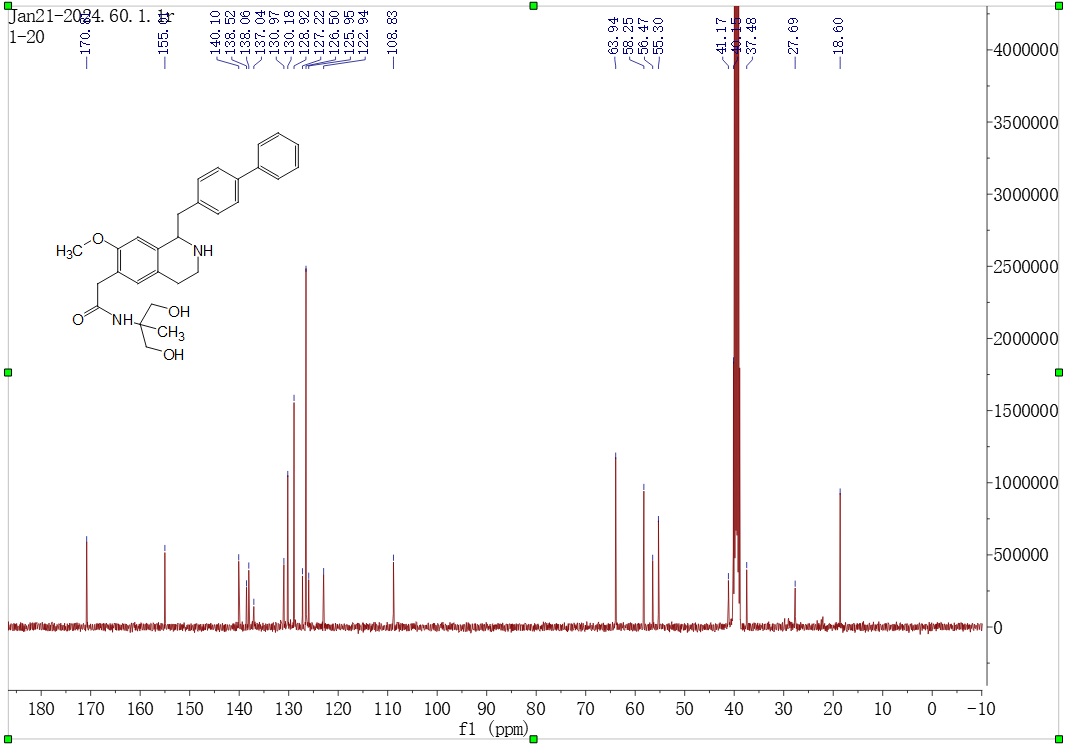
**

**Figure S36 ^13^C NMR spectrum of compound Y7k**

**
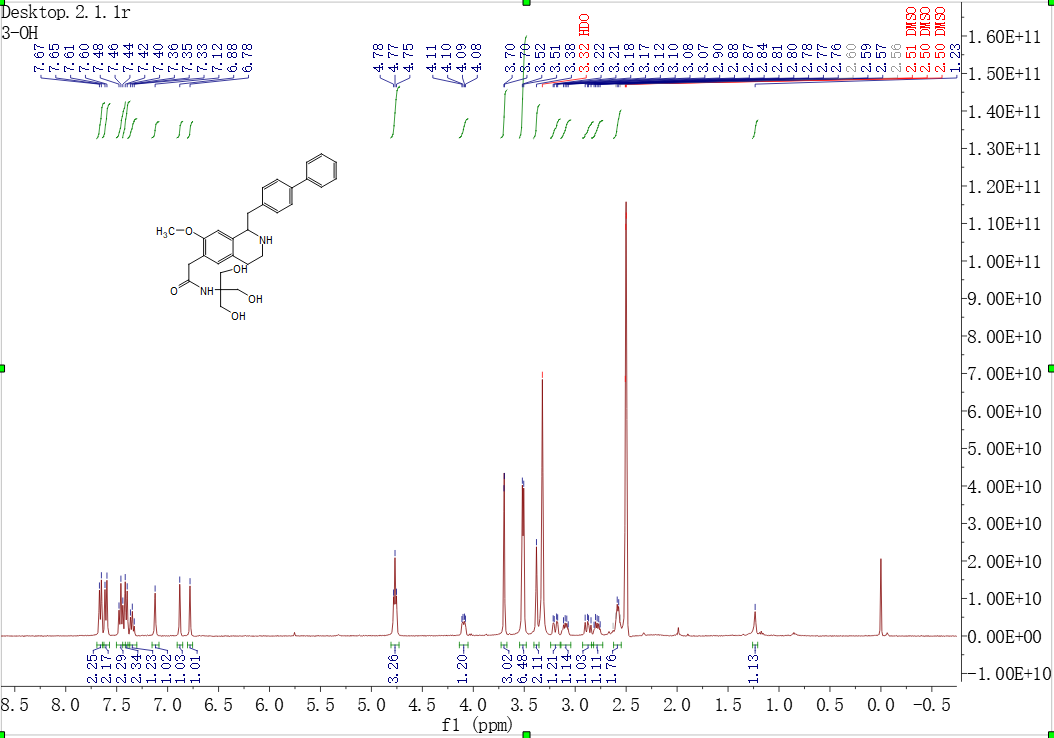
**

**Figure S37 ^1^H NMR spectrum of compound Y7l**

**
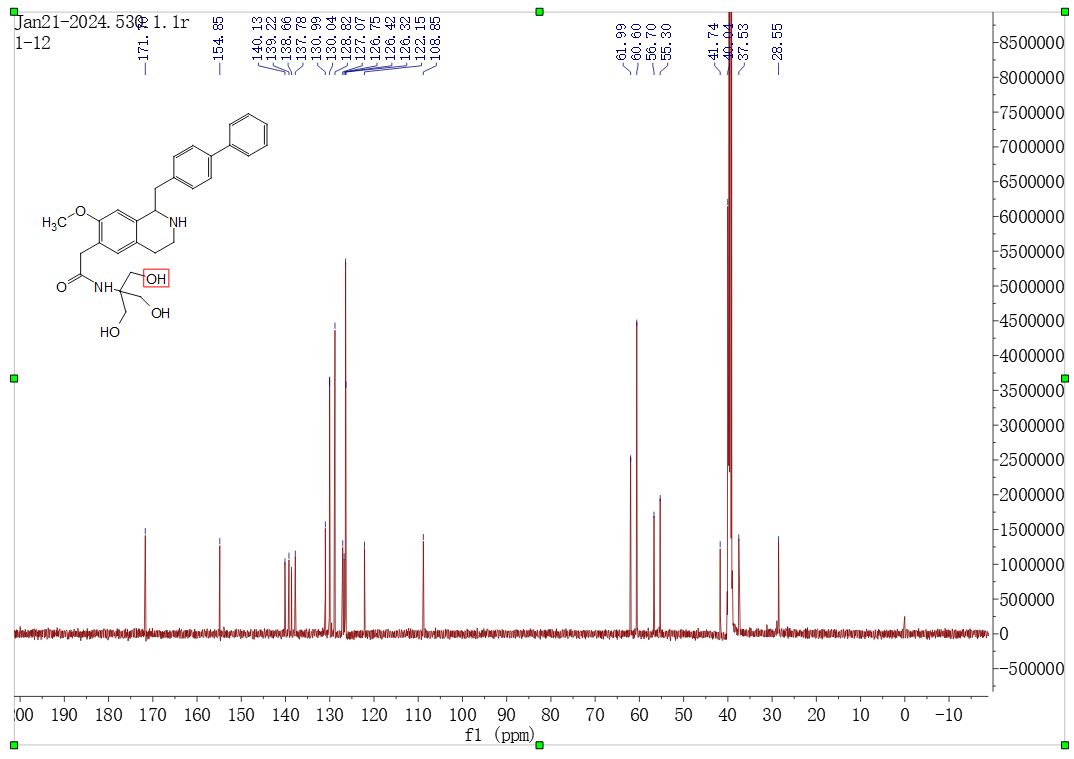
**

**Figure S38 ^13^C NMR spectrum of compound Y7l**

**
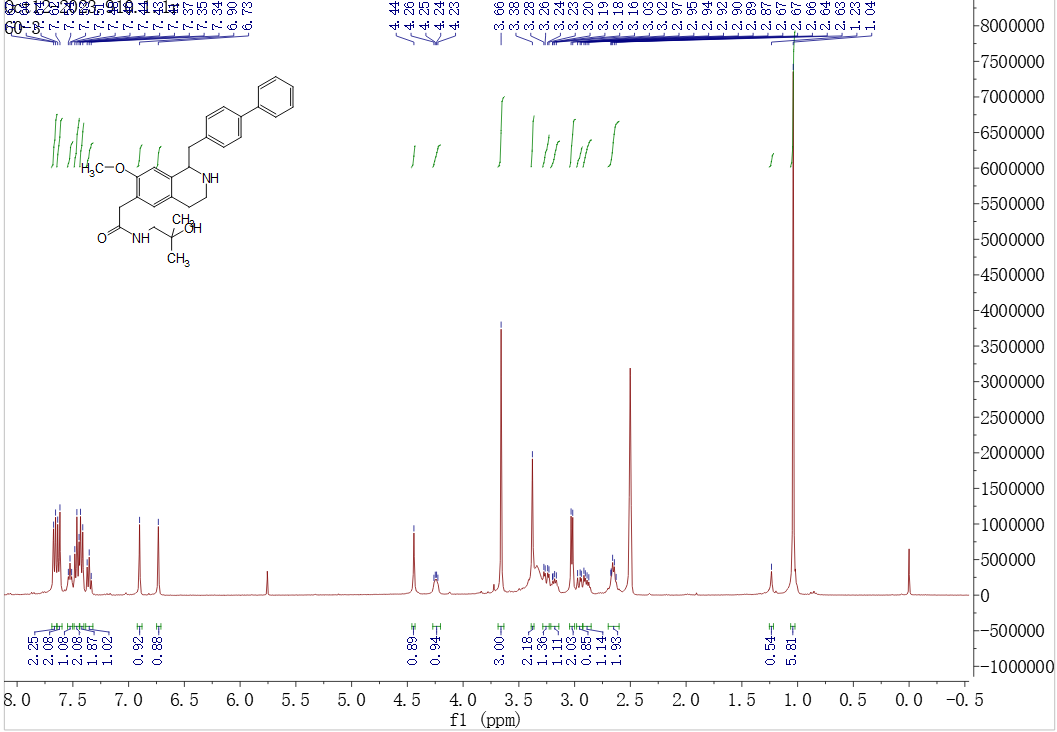
**

**Figure S39 ^1^H NMR spectrum of compound Y7m**

**
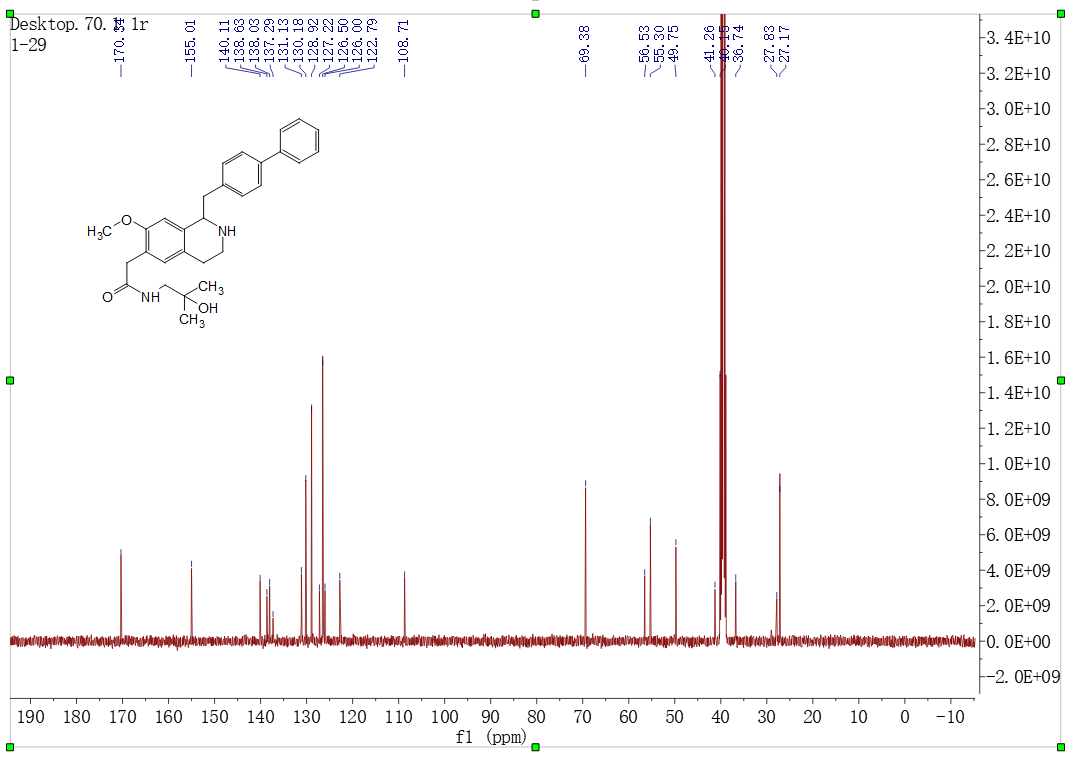
**

**Figure S40 ^13^C NMR spectrum of compound Y7m**

**
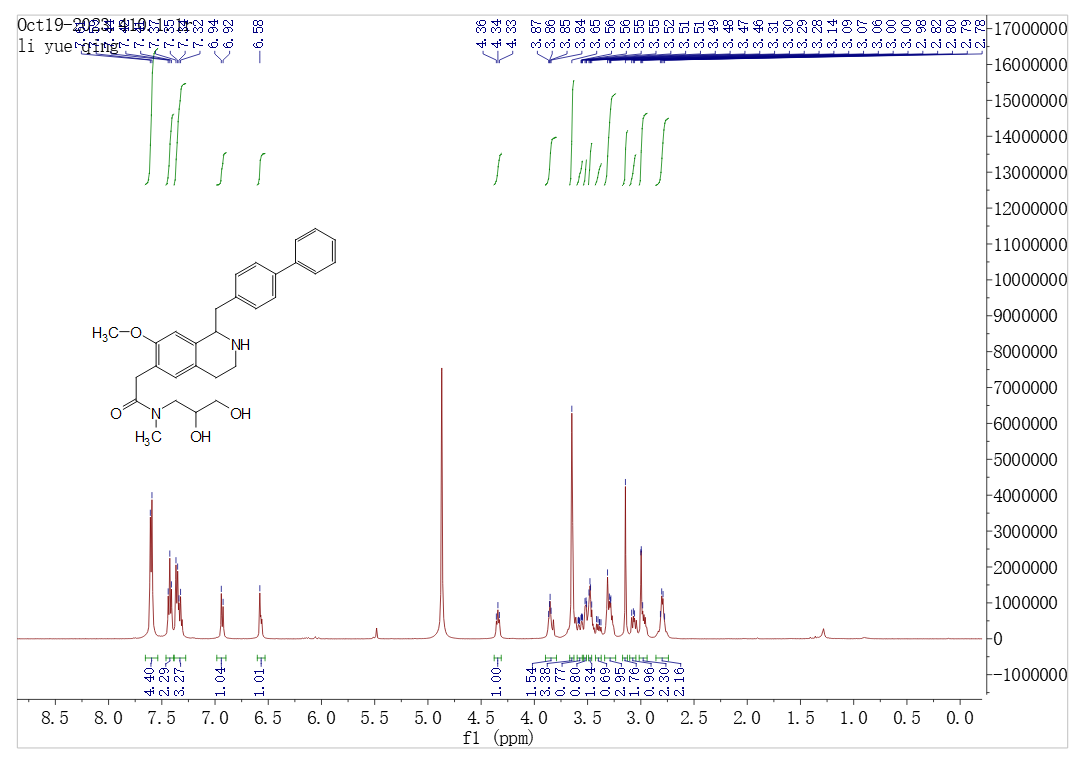
**

**Figure S41 ^1^H NMR spectrum of compound Y7n**

**
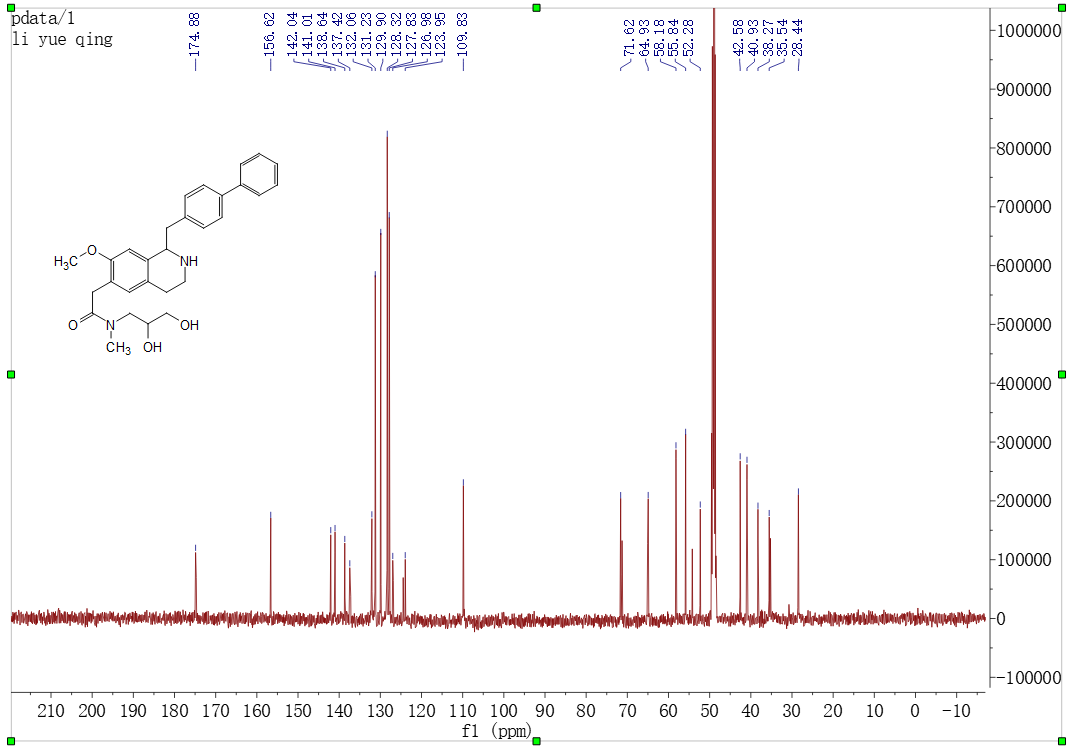
**

**Figure S42 ^13^C NMR spectrum of compound Y7n**

**
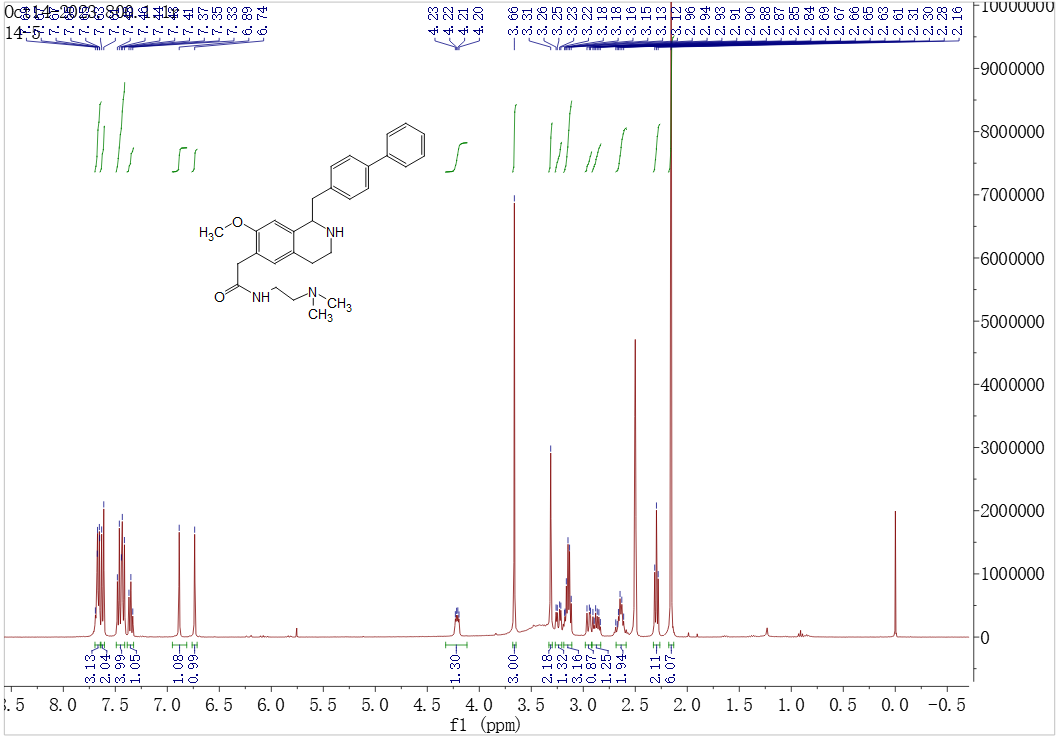
**

**Figure S43 ^1^H NMR spectrum of compound Y7o**

**
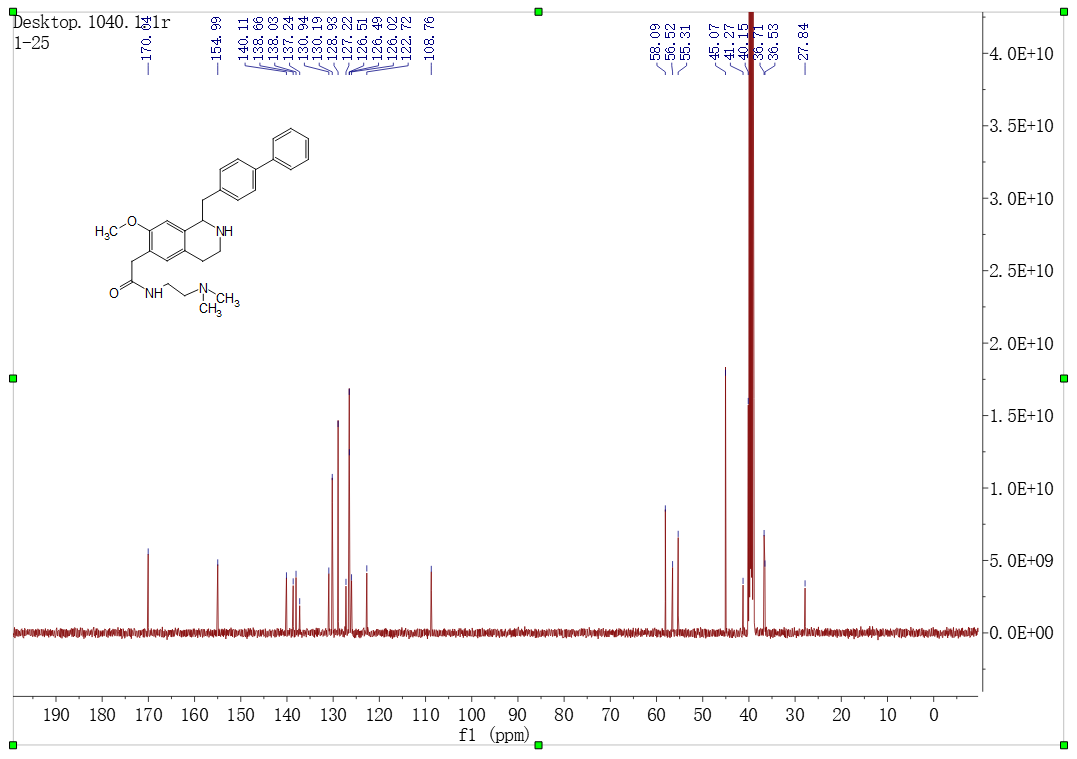
**

**Figure S44 ^13^C NMR spectrum of compound Y7o**

**
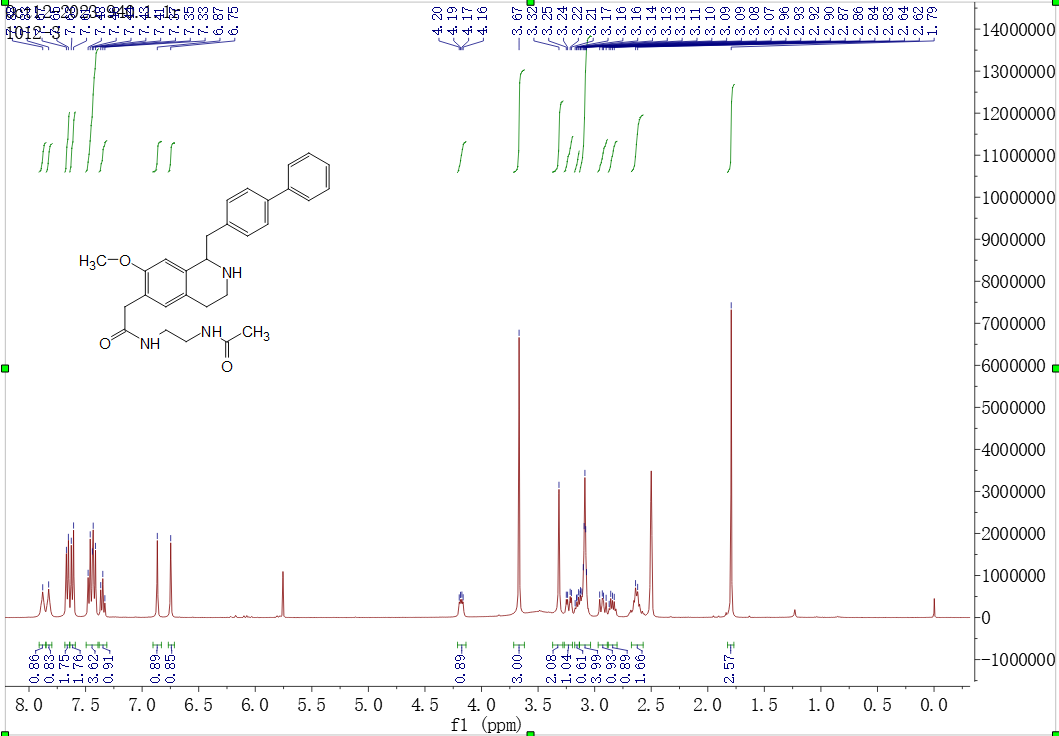
**

**Figure S45 ^1^H NMR spectrum of compound Y7p**

**
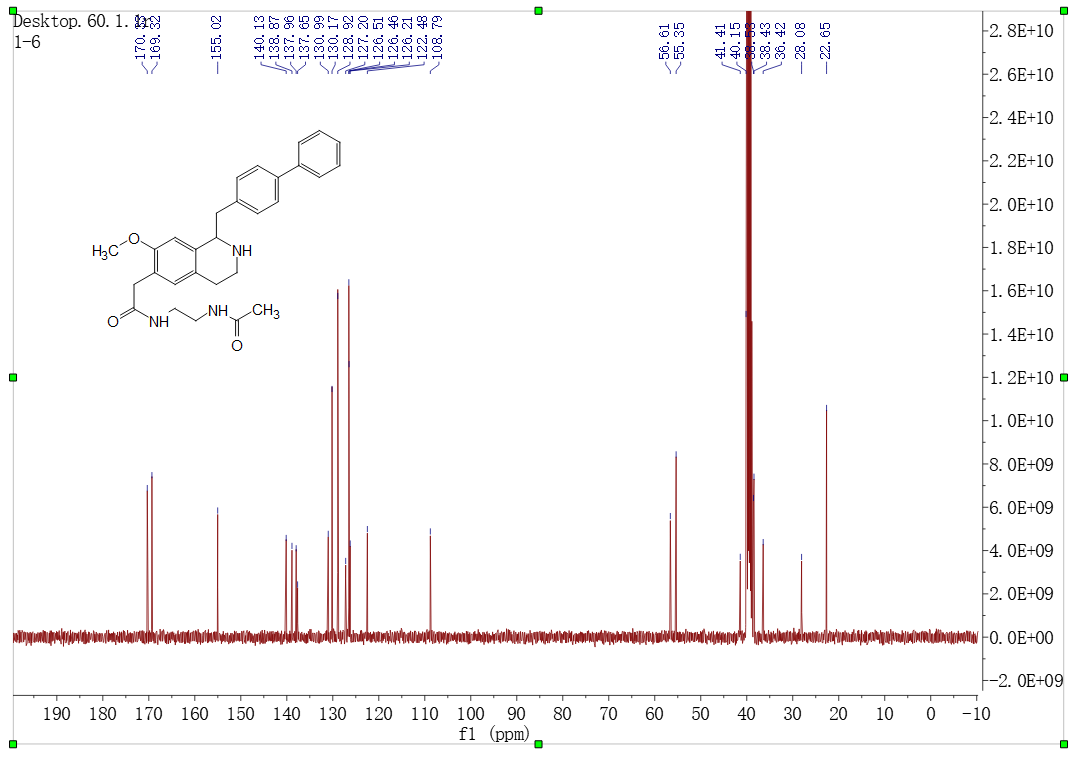
**

**Figure S46 ^13^C NMR spectrum of compound Y7p**

**
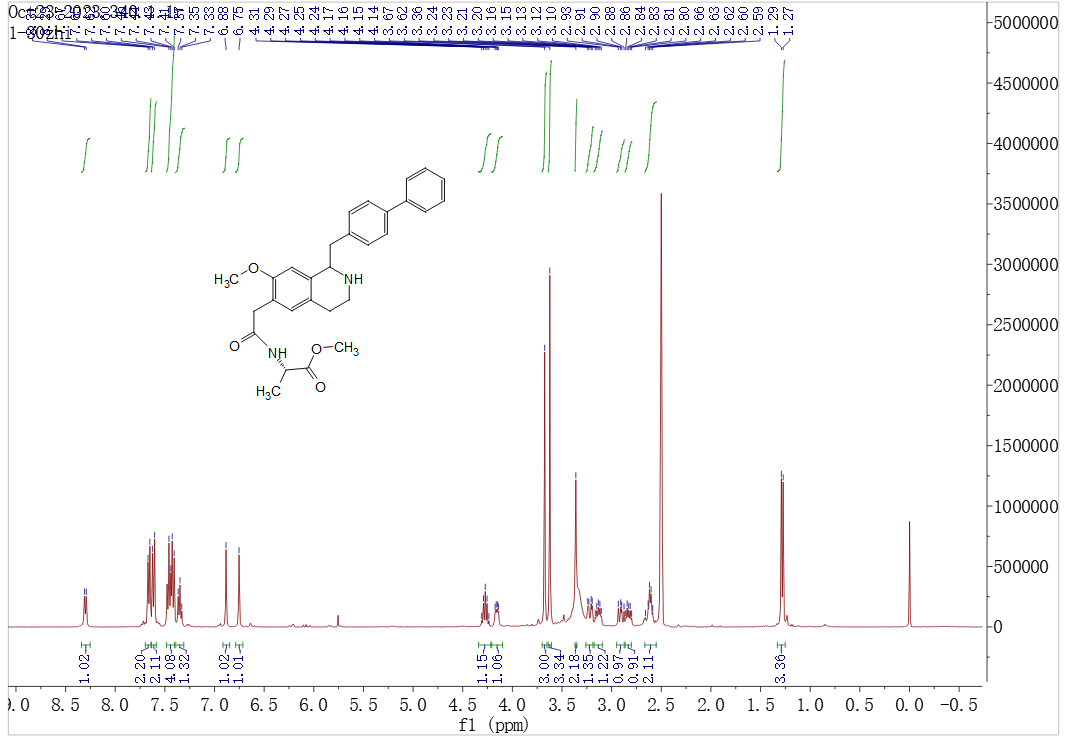
**

**Figure S47 ^1^H NMR spectrum of compound Y7q**

**
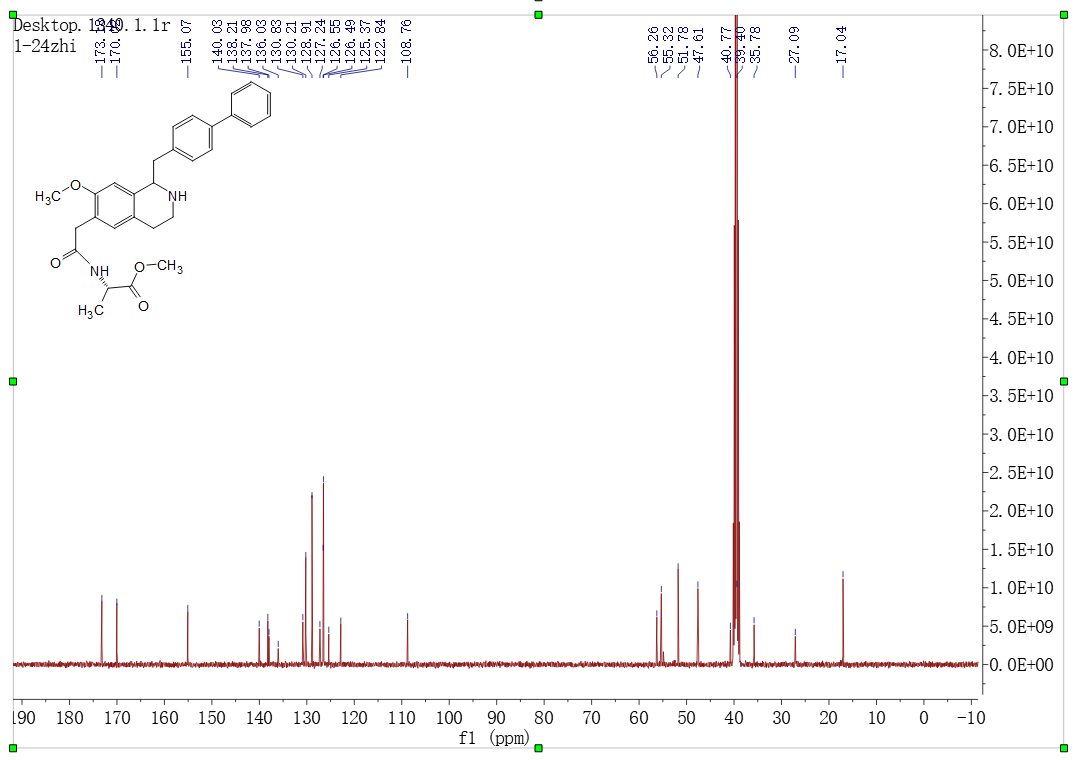
**

**Figure S48 ^13^C NMR spectrum of compound Y7q**

**
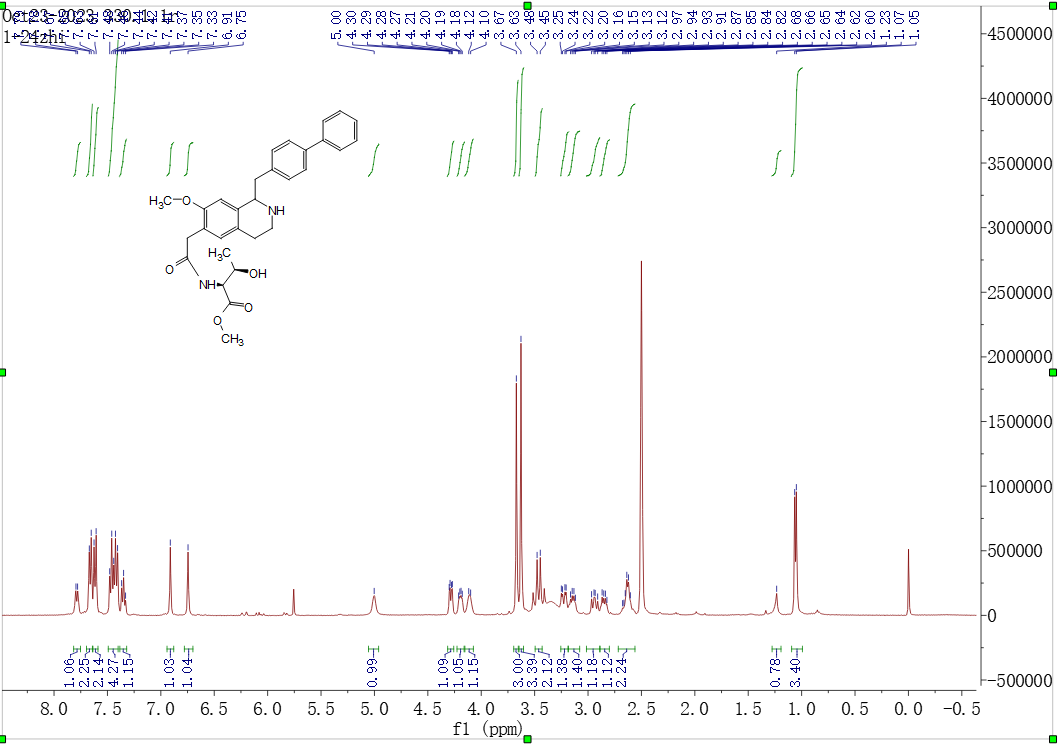
**

**Figure S49 ^1^H NMR spectrum of compound Y7r**

**
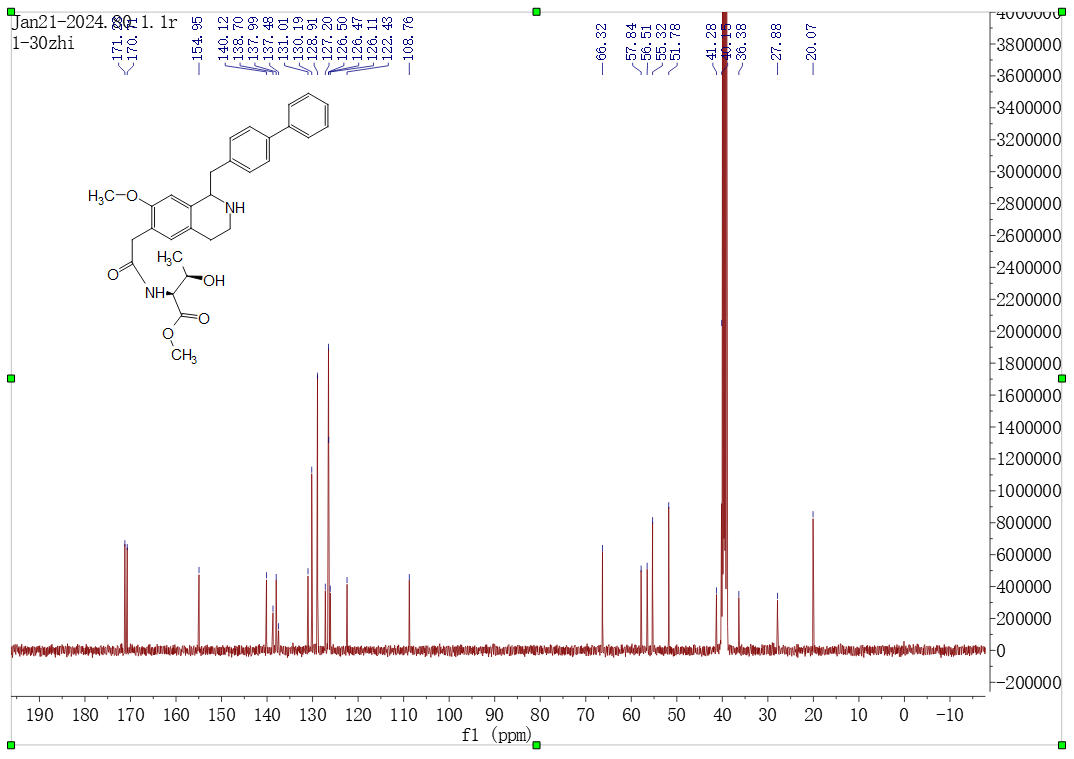
**

**Figure S50 ^13^C NMR spectrum of compound Y7r**

**
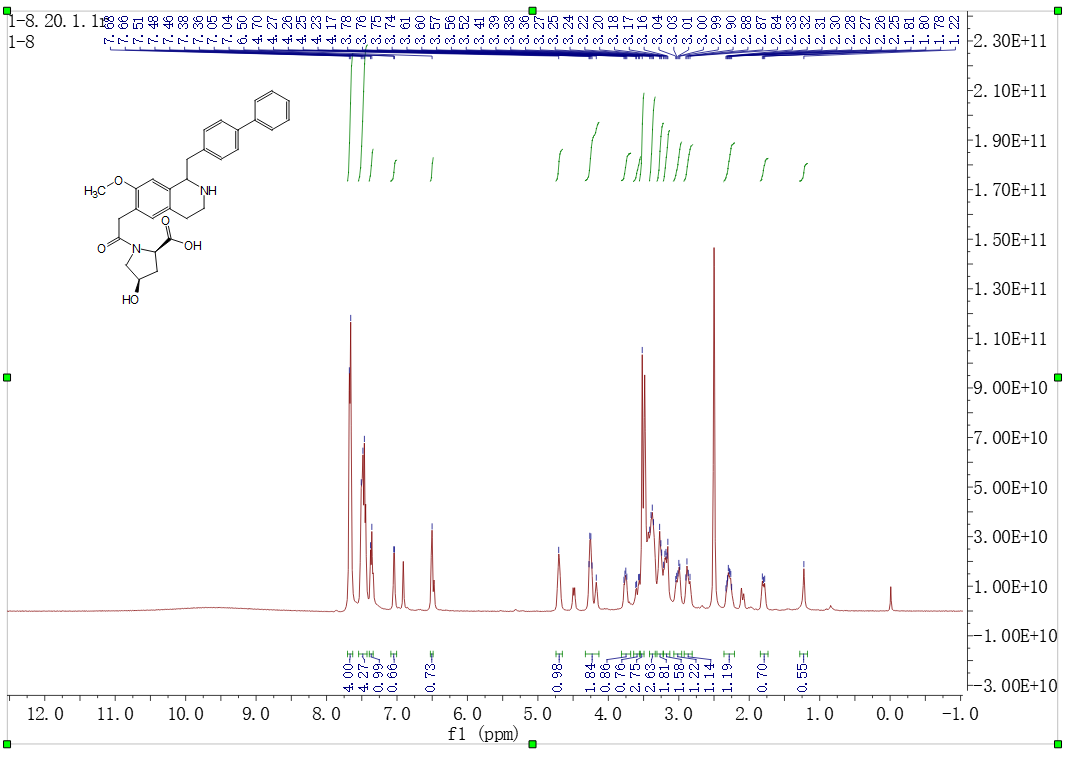
**

**Figure S51 ^1^H NMR spectrum of compound Y7s**

**
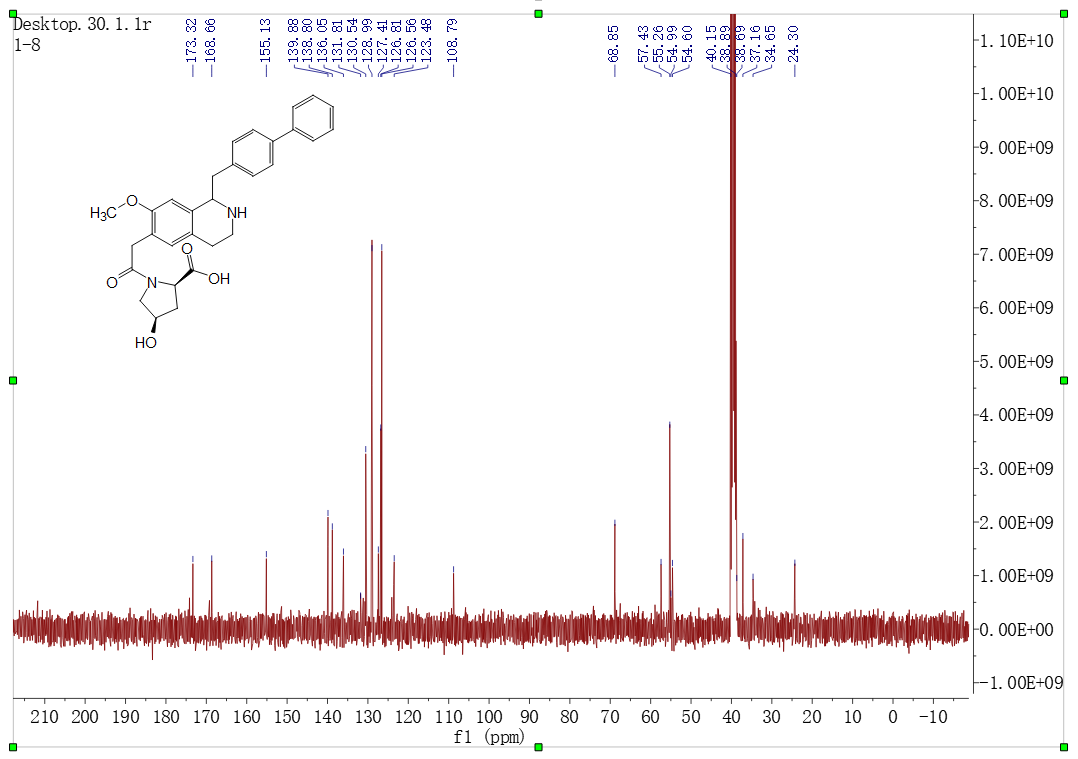
**

**Figure S52 ^13^C NMR spectrum of compound Y7s**

**
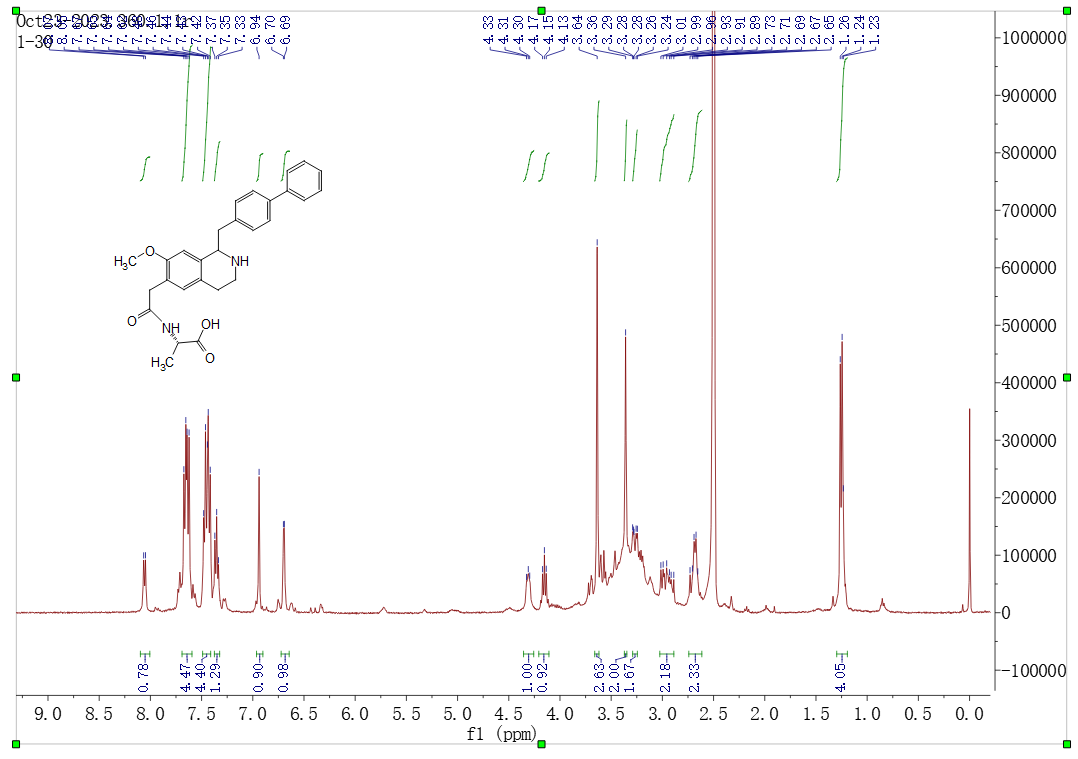
**

**Figure S53 ^1^H NMR spectrum of compound Y7t**

**
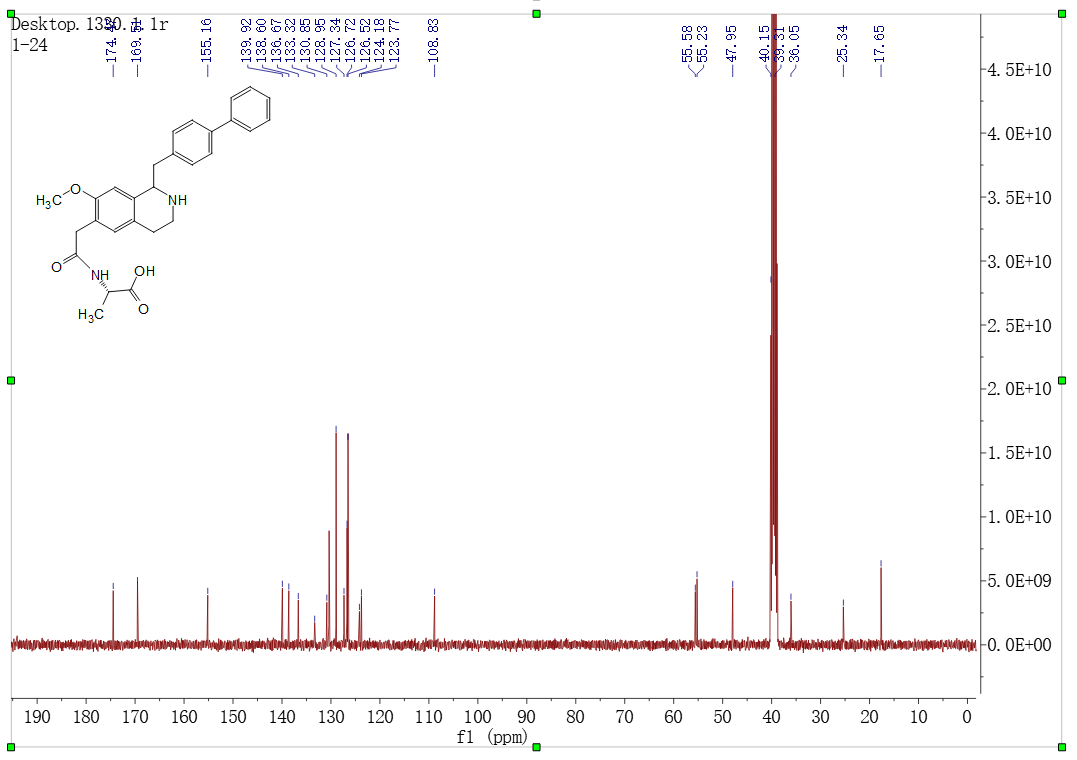
**

**Figure S54 ^13^C NMR spectrum of compound Y7t**
